# Circulating T cell status and molecular imaging may predict clinical benefit of neoadjuvant PD-1 blockade in oral cancer

**DOI:** 10.1136/jitc-2024-009278

**Published:** 2024-07-22

**Authors:** Niels E Wondergem, Iris H C Miedema, Rieneke van de Ven, Gerben J C Zwezerijnen, Pim de Graaf, K Hakki Karagozoglu, Jan-Jaap Hendrickx, Simone E J Eerenstein, Rolf J Bun, Dorien C Mulder, Jens Voortman, Ronald Boellaard, Albert D Windhorst, J Pascal Hagers, Laura A N Peferoen, Tanja D de Gruijl, Elisabeth Bloemena, Ruud H Brakenhoff, C René Leemans, C Willemien Menke-van der Houven van Oordt

**Affiliations:** 1 Amsterdam UMC location Vrije Universiteit Amsterdam, Otolaryngology/Head and Neck Surgery, De Boelelaan 1117, Amsterdam, The Netherlands; 2 Cancer Center Amsterdam, Cancer Biology and Immunology, Amsterdam, The Netherlands; 3 Amsterdam UMC location Vrije Universiteit Amsterdam, Medical Oncology, De Boelelaan 1117, Amsterdam, The Netherlands; 4 Cancer Center Amsterdam, Imaging and Biomarkers, Cancer Centre Amsterdam, Amsterdam, The Netherlands; 5 Amsterdam Institute for Infection and Immunity, Cancer Immunology, Amsterdam, Netherlands; 6 Amsterdam UMC location Vrije Universiteit Amsterdam, Radiology and Nuclear Medicine, De Boelelaan 1117, Amsterdam, The Netherlands; 7 Amsterdam UMC and Academic Centre for Dentistry Amsterdam (ACTA), Oral and Maxillofacial Surgery/Oral Pathology, De Boelelaan 1117, Amsterdam, The Netherlands; 8 Oral and Maxillofacial Surgery, Noordwest Ziekenhuisgroep, Alkmaar, The Netherlands; 9 Amsterdam UMC location Vrije Universiteit Amsterdam, Pathology, De Boelelaan 1117, Amsterdam, The Netherlands

**Keywords:** Head and Neck Cancer, Neoadjuvant, Immune Checkpoint Inhibitor

## Abstract

**Background:**

Addition of neoadjuvant immune checkpoint inhibition to standard-of-care interventions for locally advanced oral cancer could improve clinical outcome.

**Methods:**

In this study, 16 evaluable patients with stage III/IV oral cancer were treated with one dose of 480 mg nivolumab 3 weeks prior to surgery. Primary objectives were safety, feasibility, and suitability of programmed death receptor ligand-1 positron emission tomography (PD-L1 PET) as a biomarker for response. Imaging included ^18^F-BMS-986192 (PD-L1) PET and ^18^F-fluorodeoxyglucose (FDG) PET before and after nivolumab treatment. Secondary objectives included clinical and pathological response, and immune profiling of peripheral blood mononuclear cells (PBMCs) for response prediction. Baseline tumor biopsies and postnivolumab resection specimens were evaluated by histopathology.

**Results:**

Grade III or higher adverse events were not observed and treatment was not delayed in relation to nivolumab administration and other study procedures. Six patients (38%) had a pathological response, of whom three (19%) had a major (≥90%) pathological response (MPR). Tumor PD-L1 PET uptake (quantified using standard uptake value) was not statistically different in patients with or without MPR (median 5.3 vs 3.4). All major responders showed a significantly postnivolumab decreased signal on FDG PET. PBMC immune phenotyping showed higher levels of CD8^+^ T cell activation in MPR patients, evidenced by higher baseline expression levels of PD-1, TIGIT, IFNγ and lower levels of PD-L1.

**Conclusion:**

Together these data support that neoadjuvant treatment of advanced-stage oral cancers with nivolumab was safe and induced an MPR in a promising 19% of patients. Response was associated with decreased FDG PET uptake as well as activation status of peripheral T cell populations.

**WHAT IS ALREADY KNOWN ON THIS TOPIC**

- Immune checkpoint inhibition in head and neck cancer has found clinical implementation in the recurrent/metastatic setting and several recent studies have indicated it to be safe and feasible in the neoadjuvant setting. Response rates of 10%–20% are promising but warrant further understanding into which patients should be selected for treatment.

**WHAT THIS STUDY ADDS**

- We confirm the safety and effectivity of neoadjuvant nivolumab in oral cancer and show that pre-treatment peripheral CD8^+^ T cell activation levels and post-treatment changes in fluorodeoxyglucose-positron emission tomography imaging are potential indicators of response.

**HOW THIS STUDY MIGHT AFFECT RESEARCH, PRACTICE OR POLICY**

- This study underscores the feasibility of neoadjuvant treatment with nivolumab in patients with oral squamous cell carcinoma and identifies immunobiological parameters which could guide further investigations into response prediction.

## Introduction

 Oral squamous cell carcinoma (OSCC) originates in the mucosal lining of the mouth and affects more than 370,000 new patients worldwide annually.^1^ Typical risk factors for developing OSCC include tobacco use and alcohol consumption. The mainstay of treatment for OSCC is surgical excision. For locally advanced cases an extensive resection including cervical neck dissection is required, followed by reconstruction by, for example, a free vascularized flap, as well as postoperative radiotherapy or chemoradiotherapy when high-risk features are present.^2^ Even with these intensive treatment regimens, 40%–50% of these patients will develop local recurrence or distant metastases with minimal curative salvage treatment options. Immunotherapy seems highly promising to increase disease control in this difficult to manage patient group.

Trials in recurrent or metastatic head and neck squamous cell carcinoma (HNSCC) have shown that 10%–20% of patients have durable responses following treatment with immune checkpoint inhibitors (ICIs) targeting programmed death receptor-1 (PD-1).^3^,^6^ These are paradigm shifting results in head and neck oncology that have resulted in approval of the PD-1 inhibitors nivolumab and pembrolizumab as second-line treatment for recurrent/metastatic or inoperable disease after progression on platinum-based therapy. Pembrolizumab was also approved as first-line monotherapy or in combination with platinum based chemotherapy for patients with metastasized or inoperable disease. Immunotherapies have gained interest in the neoadjuvant setting in treatment-naïve patients with the potential advantages of reducing tumor volume before surgery and eliminating micrometastases, with the intention to reduce the extent of the resection, and increasing long-term survival. Over the past 2 years, several trials have been published on neoadjuvant ICIs in HNSCC showing highly encouraging results.^7^,^11^ A variety of regimens have been evaluated which included single agent ICI, dual agent ICI and ICI combined with conventional chemotherapy or radiotherapy. Responses are sometimes remarkable, but so far only a minority of patients respond. Whether patients are treated in the neoadjuvant setting or otherwise, it is imperative to find biomarkers to identify the patients who will benefit from PD-1 blockade. Both patient and tumor characteristics might play a role in response prediction. PD-L1 expression has been studied as an obvious biomarker candidate as it might correlate with an improved response rate in HNSCC as well as other tumor types. However, treatment selection based on PD-L1 expression in biopsies is suboptimal.^12^ Whole body imaging using positron emission tomography (PET) with a radiolabeled adnectin tracer targeting PD-L1 (ie, ^18^F-BMS-986192 PET) has been proposed as suitable non-invasive alternative.^13^ Additionally, PD-L1 PET can provide information on heterogeneity of PD-L1 expression and, if repeated at several time points, on dynamics of PD-L1 expression. Previous studies have shown promising results in predicting response in lung cancer or melanoma patients treated with ICI.^13 14^ Specifically when applied in a neoadjuvant setting, much knowledge can be gained by comparing pre- and post-treatment imaging and blood/tumor samples to study the effect of ICIs.

This single-center phase II trial was designed to assess the safety and feasibility of neoadjuvant nivolumab in a treatment-naïve cohort of patients with primary advanced stage OSCC. Moreover, we investigated PD-L1 and fluorodeoxyglucose (FDG) PET imaging as well as immunohistochemistry-based PD-L1 expression and peripheral blood immune profiling to identify predictors for neoadjuvant PD-1 immune checkpoint blockade in a well-defined OSCC patient cohort.

## Results

### Patient characteristics

Between March 1, 2019 and July 31, 2021, 17 patients were enrolled. One patient was excluded from the study as the tumor was considered inoperable during the diagnostic workup. The remaining 16 patients were predominantly male (69%) and had a median age of 72 years (range 49–86). The majority of patients had a history of tobacco and/or alcohol consumption. All patients had American Joint Committee on Cancer (AJCC, eighth edition) stage III/IV disease. Baseline patient characteristics are listed in table 1 and online supplemental table S1. All 16 patients received neoadjuvant nivolumab treatment according to study protocol. Prior to nivolumab administration the mucosa adjacent to the tumor was marked by tattooing for adequate post-nivolumab resection in case of tumor response.

**Table 1:** Patient characteristics

	Patients (n = 16)	
	Count (%)	Median (range)
Age at diagnosis		72 (49–86)
Sex		
Male	11 (69)	
Female	5 (31)	
Smoking		
Current	6 (38)	
Former	6 (38)	
Never	4 (25)	
Packyears		21 (0–104)
Alcohol		
Regular	10 (63)	
Seldom	1 (6)	
Former	3 (19)	
Never	2 (13)	
Tumor subsite		
Border of tongue	3 (19)	
Gingiva	8 (50)	
Floor of mouth	4 (25)	
Cheek	1 (6)	
T-stage*		
T3	5 (31)	
T4a	11 (69)	
N-stage*		
N0	10 (63)	
N1	3 (19)	
N2b	2 (13)	
N3b	1 (6)	
Stage*		
III	3 (19)	
IVa	12 (75)	
IVb	1 (6)	

* TNM 8theighth edition.

### Safety and feasibility

All 16 patients were assessed for adverse events (AEs) that were deemed possibly related to neoadjuvant nivolumab. Grade I and grade II AEs occurred in 12 (75%) and 4 (25%) patients, respectively. A total of 43 grade I/II AEs were reported; no AEs over grade II were observed. The most common AEs were blood laboratory abnormalities, which in most cases did not necessitate any treatment. An overview of all AEs is given in table 2. Six serious AEs (SAEs) were observed in four patients, which included pneumonia, left ventricular systolic dysfunction, stroke, malnutrition requiring hospitalization, and anemia requiring hospitalization. None of the SAEs were deemed related to neoadjuvant nivolumab but associated with the age and comorbidities of these patients. An overview of SAEs is given in table 3.

**Table 2:** Overview of adverse events

CTCAE v5.0 term	Grade I	Grade II	Grade≥III
Blood laboratory abnormality	14	3	0
Diarrhea	6	0	0
Constipation	4	0	0
Vomiting	1	2	0
Insomnia	3	0	0
Eczema	0	2	0
Edema limbs	0	1	0
Urine retention	0	1	0
Atrial flutter	1	0	0
Ear pain	1	0	0
Sinus bradycardia	1	0	0
Supraventricular tachycardia	1	0	0
Fatigue	1	0	0
Dermatitis	1	0	0

CTCAE, Common Terminology Criteria for Adverse Event

**Table 3:** Overview of serious adverse events (SAEs)

ID	SAE	Study phase	Post-nivolumab	Post-surgery	Outcome
009	Pneumonia	Postoperative	60 days	31 days	Patient deceased
011	Hospitilization due to malnutrition	Preoperative	15 days	NA	Resolved
011	Hospitilization due to anemia	Postoperative	33 days	13 days	Resolved
016	Left ventricular systolic dysfunction	Preoperative	6 days	NA	Resolved
017	Stroke	Postoperative	23 days	1 day	Severe neurological deficits
017	Pneumonia	Postoperative	31 days	9 days	Patient deceased

NAnot available

All 16 patients underwent surgery as planned. Importantly, we did not observe any unexpected delays in surgery due to study procedures or AEs. The median interval between neoadjuvant nivolumab treatment and surgery was 24 days (range 20–46 days). The patient with the longest interval between nivolumab and surgery (46 days) had experienced delay due to a de novo heart murmur detected during screening which necessitated a detailed cardiological evaluation, leading to rescheduling of the surgery. There were no unexpected surgical complications observed in any of the patients suspected to be related to nivolumab therapy.

**Figure 1:**
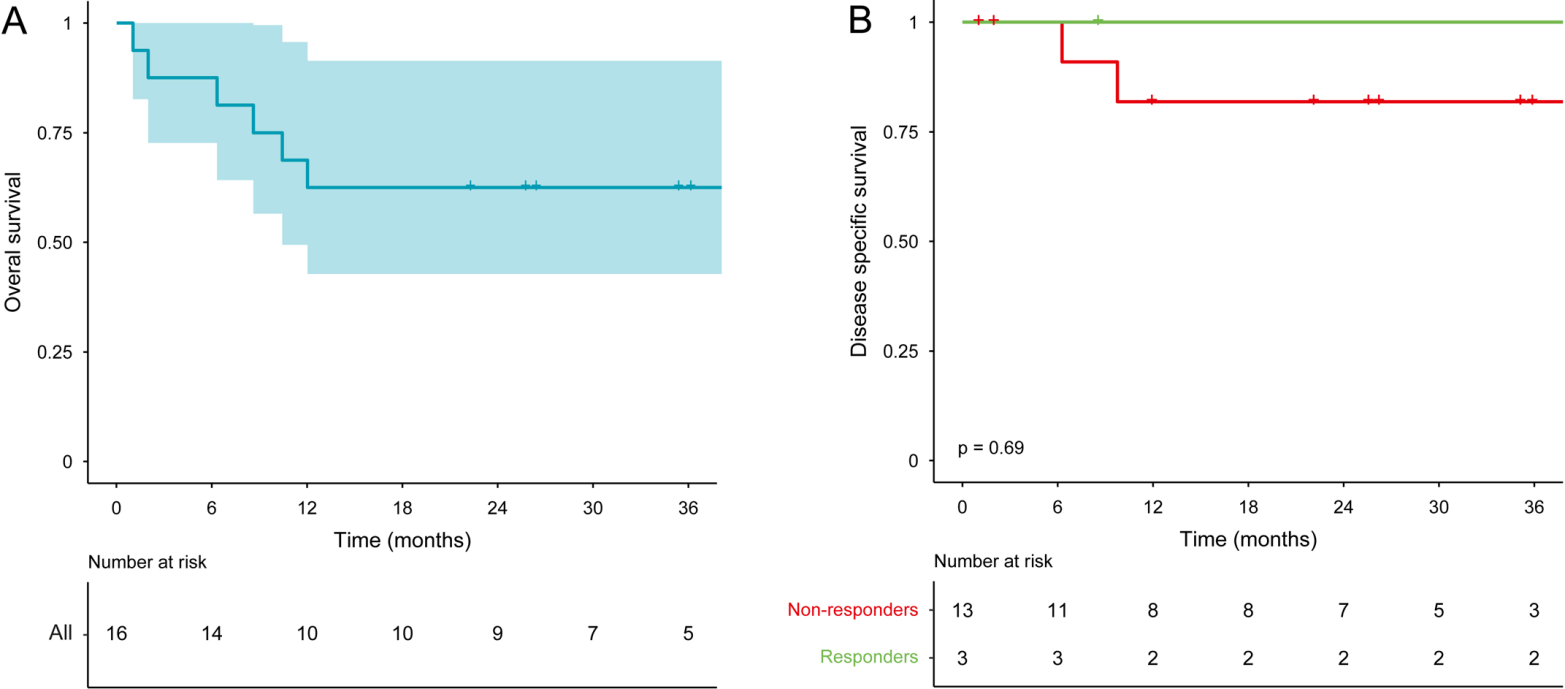
(A) 3-year overall survival of nivolumab treated patients (n = 16). Median survival was not reached. (B) 3-year disease-specific survival stratified by response.

Nine patients received adjuvant radiotherapy and four patients received adjuvant chemoradiotherapy, according to standard of care. One patient had an indication for adjuvant radiotherapy based on the initial tumor staging but opted out after a major response to nivolumab and fear of radiotherapy-induced toxicity (online supplemental table S1). Two patients deceased prior to adjuvant therapy due to pneumonia. The 3-year overall survival is shown in figure 1A. Median follow-up time was 35 months (range 21–49), median survival was not reached at the date of this report. During follow-up, four patients passed away, of whom two due to tumor recurrence and two due to non-tumor related causes (ie, one from a cardiac arrest and one from a traumatic head injury). The 3-year disease-specific survival with disease-related death and recurrent disease as events is shown in figure 1B.

### Radiographic response evaluation

**Figure 2:**
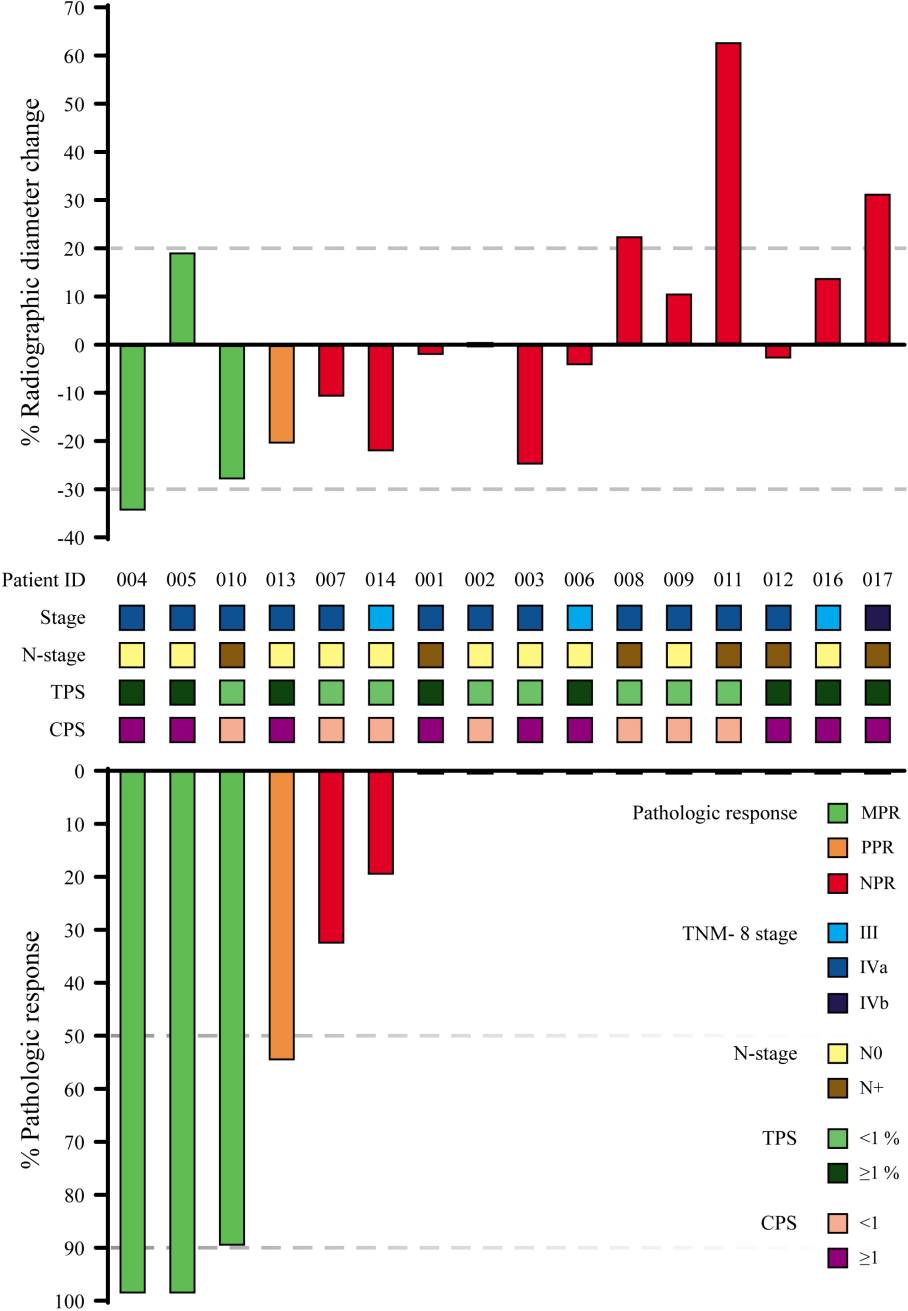
Top: Percentage radiographic diameter change on conventional imaging (MRI/CT). Dashed gray lines indicate RECIST v1.1 thresholds for partial response (≥30% diameter decrease) and progressive disease (≥20% increase). The range in between the dashed lines represents stable disease. Bottom: Overview of pathological response and clinicopathological parameters. Dashed gray line indicate thresholds for major pathological response (MPR; ≥90%), partial pathological response (PPR; ≥50%–90%) and no pathological response (NPR; <50%). CPS, combined positivity score; TPS, tumor proportion score.

All 16 patients underwent standard baseline imaging either by MRI (n = 15) or CT (n = 1), followed by postnivolumab imaging for response assessment just prior to surgery using the same imaging modality. Postnivolumab imaging was performed at a median of 19 days after nivolumab therapy (range 14–26). The median change in tumor diameter between prenivolumab and postnivolumab imaging was −2.6% (range −34.6% to +62.9%). As per the RECIST V.1.1 criteria, 1 patient was classified as responder, 12 patients were classified as having radiographic stable disease and 3 patients as having radiographic progressive disease (figure 2).

### Pathological response evaluation

**Figure 3:**
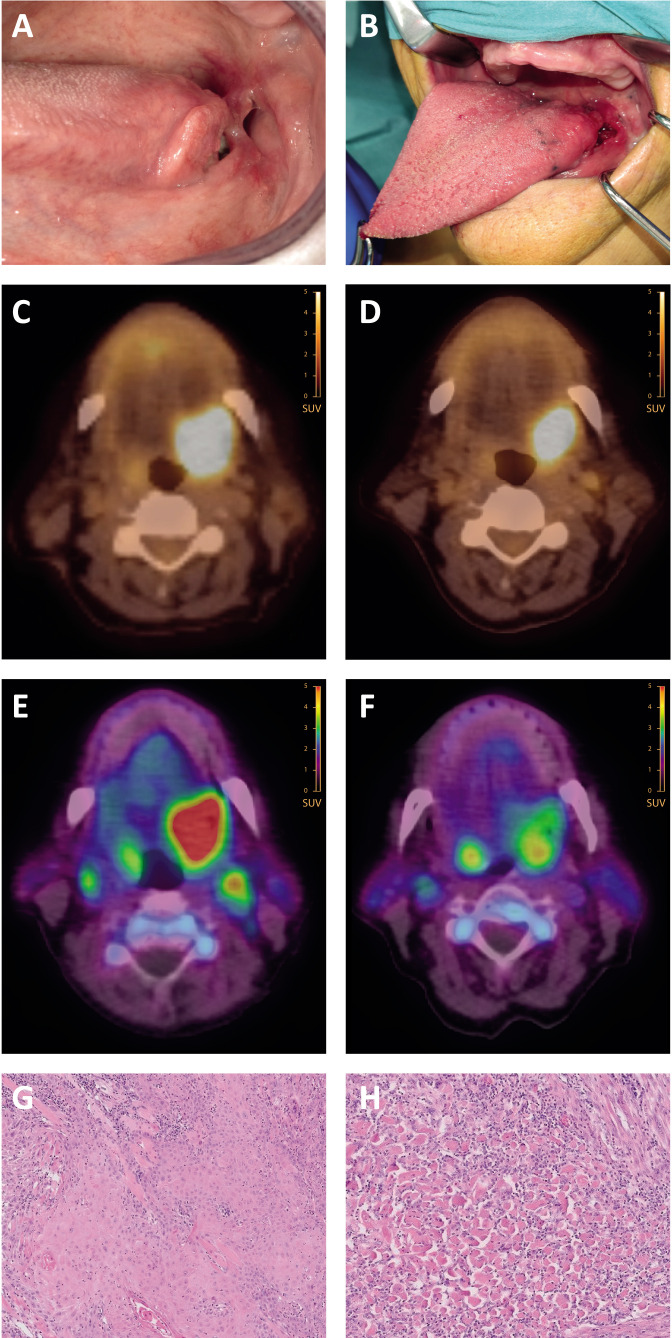
(A, B) Clinical presentation of patient 005 at baseline (**A**) and post-nivolumab (**B**). (C, D) ^18^F-FDG uptake at baseline (**C**) and post-nivolumab (**D**). (E, F) ^18^F-BMS-986192 uptake at baseline (**E**) and post-nivolumab (**F**). (G, H) HE staining (×5 zoom) at baseline (**G**) and post-nivolumab (**H**). Note the absence of viable tumor and increase of fibrosis and keratinous debris. ^18^F-FDG, 18F-fluorodeoxyglucose; HE, hematoxylin-eosin

The resection specimens of all 16 patients were evaluated for pathological response. Three patients (19%) had a major pathological response (MPR), 1 patient (6%) had a partial response, 2 patients (13%) had a minor response and 10 patients (63%) had no evidence of pathological response (figure 2). Neither the pre-treatment tumor proportion score (TPS) nor the combined positivity score (CPS) correlated with pathological response. An example of a major responder is shown in figure 3, an overview of all pathological assessments including PD-L1 scoring is shown in online supplemental table S2.

### PET imaging

#### Uptake of ^18^F-BMS-986192 in tumor lesions at baseline and after treatment with nivolumab

PD-L1 PET scans were performed at baseline (n=15) and follow-up (n=14) and included 14 matched baseline/follow-up pairs. For two patients, the PD-L1 PET imaging series were incomplete as the ^18^F-BMS-986192 batch did not pass quality control: that is, for patient 003 the pre-nivolumab and post-nivolumab PD-L1 PET and for patient 014 the post-nivolumab PD-L1 PET. These patients were excluded from the overall PD-L1 PET analysis.

**Figure 4:**
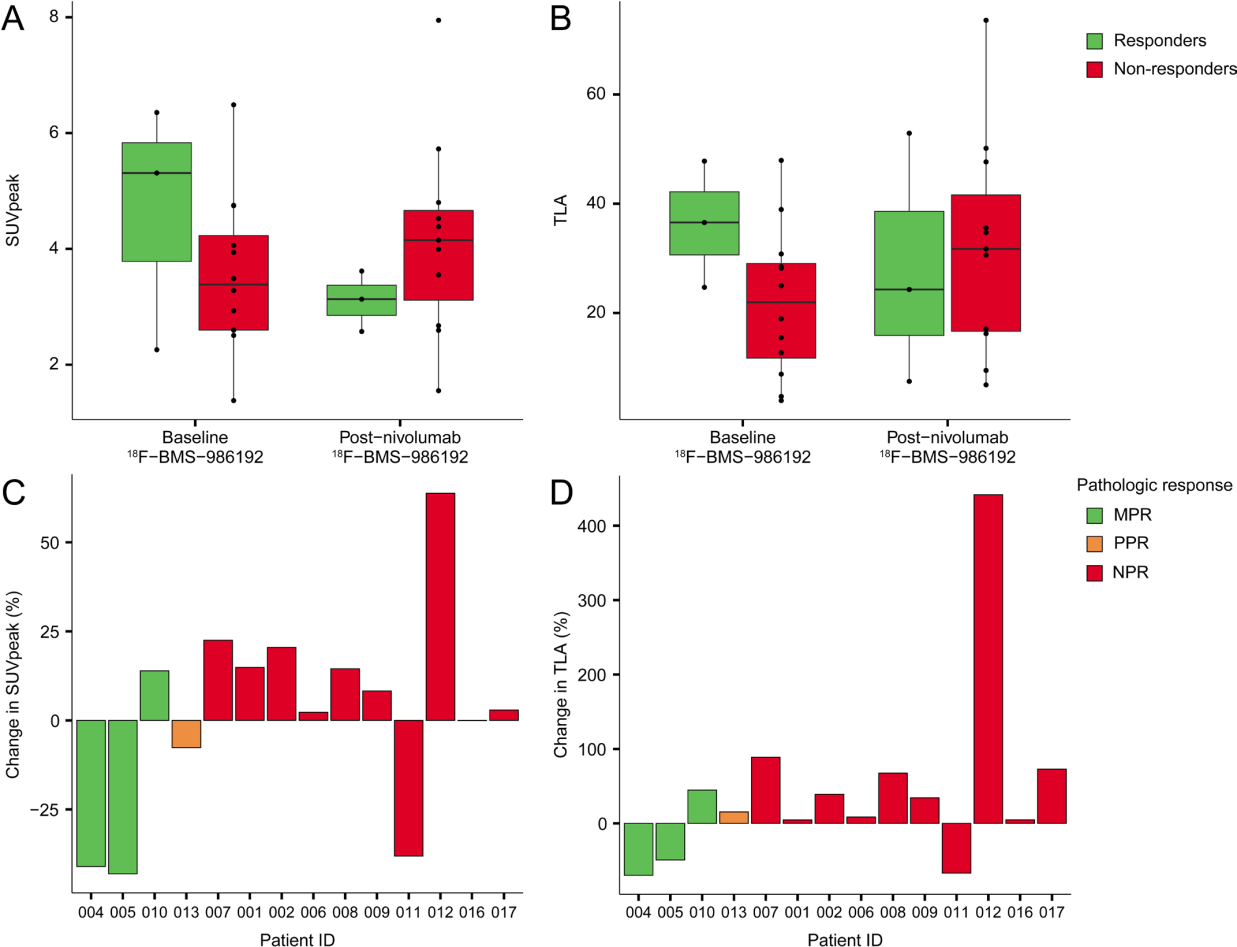
Baseline and post-nivolumab uptake of ^18^F-BMS-986192 in primary tumors stratified per response (responders vs non-responders). (A) SUV_peak_. (B) TLA. A decrease in SUV_peak_ (C) and TLA (D) identified two out of three patients with MPR. Additionally, one non-responder also showed marked decrease in both SUV_peak_ and TLA. MPR, major pathological response; NPR, no pathological response; PPR, partial pathological response; SUV, standard uptake value; TLA, total lesion activity.

Uptake as measured by standard uptake value (SUV)_peak_ of ^18^F-BMS-986192 in tumors at baseline was median 3.5 (IQR 2.6–4.7) and did not change significantly at follow-up with SUV_peak_ median 3.8 (IQR 2.8–4.5; paired Wilcoxon test, n=14, p=0.58). At baseline, responders had a numerically higher ^18^F-BMS-986192 SUV_peak_ (median 5.3, IQR 3.8–5.8) than non-responders (median 3.4, IQR 2.6–4.2), but this difference was not statistically significant (Wilcoxon test, n=15, p=0.54) (see figure 4A). A similar pattern was seen for ^18^F-BMS-986192 total lesion activity (TLA=SUV_mean_× volume) (figure 4B).

A decrease in uptake of ^18^F-BMS-986192 (as demonstrated in SUV_peak_ and TLA) after treatment with nivolumab was noted in two out of three responders (figure 4C). However, one non-responder (patient 011) also showed a decreased ^18^F-BMS-986192 uptake after treatment. Neither PD-L1 CPS nor PD-L1 TPS of baseline biopsies corresponded to baseline ^18^F-BMS-986192 uptake. A ^18^F-BMS-986192 SUV_peak_ cut-off of >4 identified three out of four patients with MPR or PPR (online supplemental figure S1). PD-L1 CPS with a cut-off ≥1 performed similarly (identified three out of four patients with MPR or PPR).

#### Uptake of ^18^F-FDG in tumor lesions at baseline and after treatment with nivolumab

**Figure 5:**
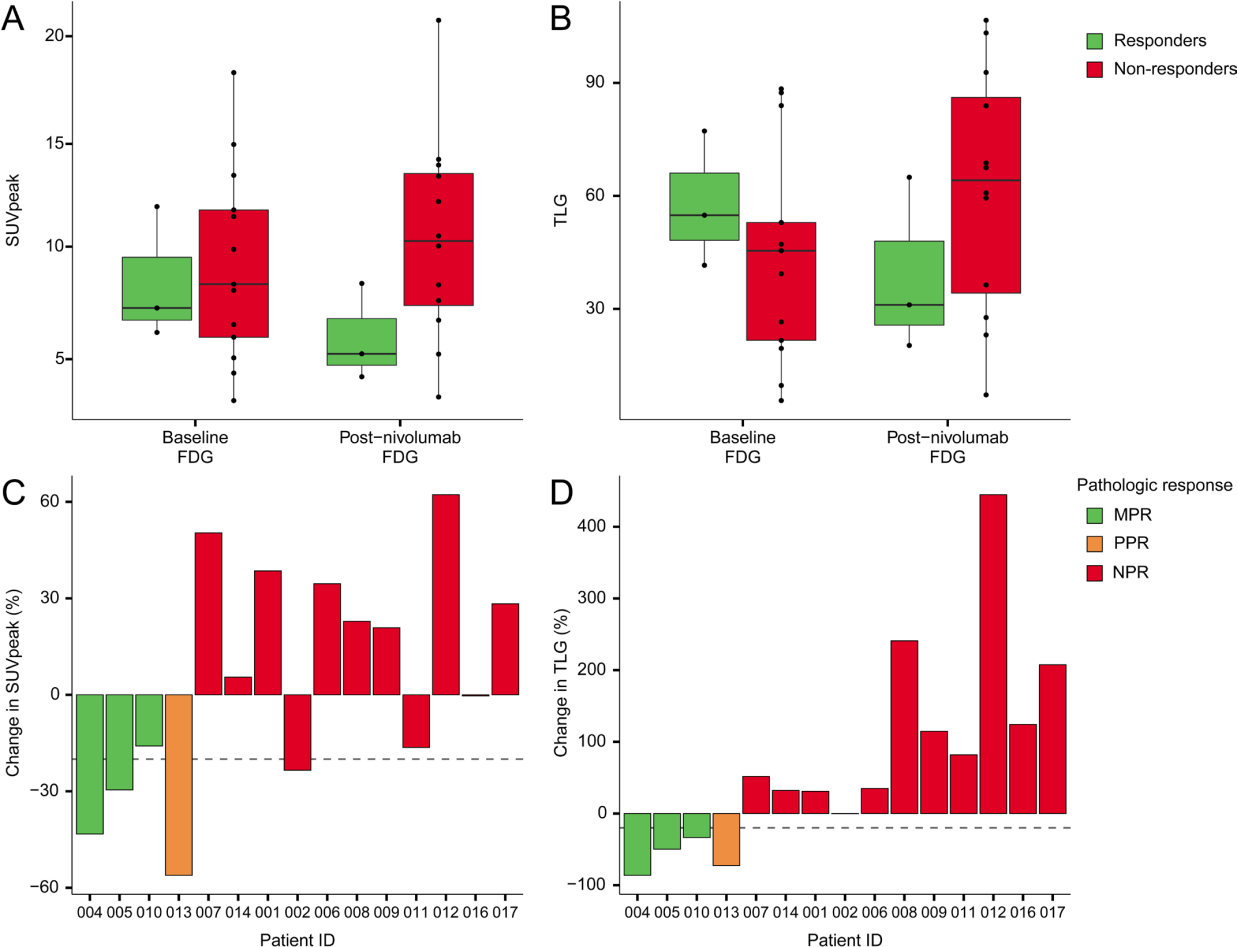
Baseline and post-nivolumab uptake of ^18^F-FDG in primary tumors stratified per response (responders vs non-responders). (A) SUV_peak_. (B) TLG. (C) ^18^F-FDG SUV_peak_ decreases in two out of three patients with MPR. The highest decrease is seen in patient 013 with a partial response, and a decrease was also seen in two patients with no response. (D) A decrease of more than 20% in ^18^F-FDG TLG identified four out of four partial or major responders while not seen in non-responders. ^8^F-FDG, 18F-fluorodeoxyglucose; MPR, major pathological respons; SUV, standard uptake value; TLG, total lesion glycolysis.

FDG PET scans were performed at baseline (n=16) and post-nivolumab (n=15) and included 15 matched pairs. Baseline ^18^F-FDG SUV_peak_ of tumors was median 8.3 (IQR 6.2–12.0) and did not change significantly with SUV_peak_ median 8.5 (IQR 6.0–13.0) postnivolumab (paired Wilcoxon test, n=15, p=0.80). At baseline, non-responders had a higher ^18^F-FDG SUV_peak_ than responders, but this difference was not statistically significant (Wilcoxon test, n=16, p=1, figure 5A). A similar pattern was observed for ^18^F-FDG total lesion glycolysis (TLG=SUV_mean_×volume, figure 5B). A decrease in ^18^F-FDG SUV_peak_ was observed in all patients with MPR or PPR (figure 5C). Moreover, a decrease in ^18^F-FDG TLG of 20% (dashed line, postulated as cut-off for response by Vos *et al*) was only seen in patients with MPR or PPR and was not present in non-responders (figure 5D).^11^

### Peripheral immunomonitoring

Flowcytometric immune profiling of peripheral blood mononuclear cells (PBMCs) was performed at all time points for all patients, except for patients 009 and 017 who deceased prior to T=3. PD-1 frequencies among lymphocytic subsets were expressed as total percentage of PD-1 positive cells, PD-1^int^ and PD-1^high^ (online supplemental figure S2B).^15 16^

#### Pre-nivolumab peripheral T cell and NK cell subset frequencies and activation state

**Figure 6:**
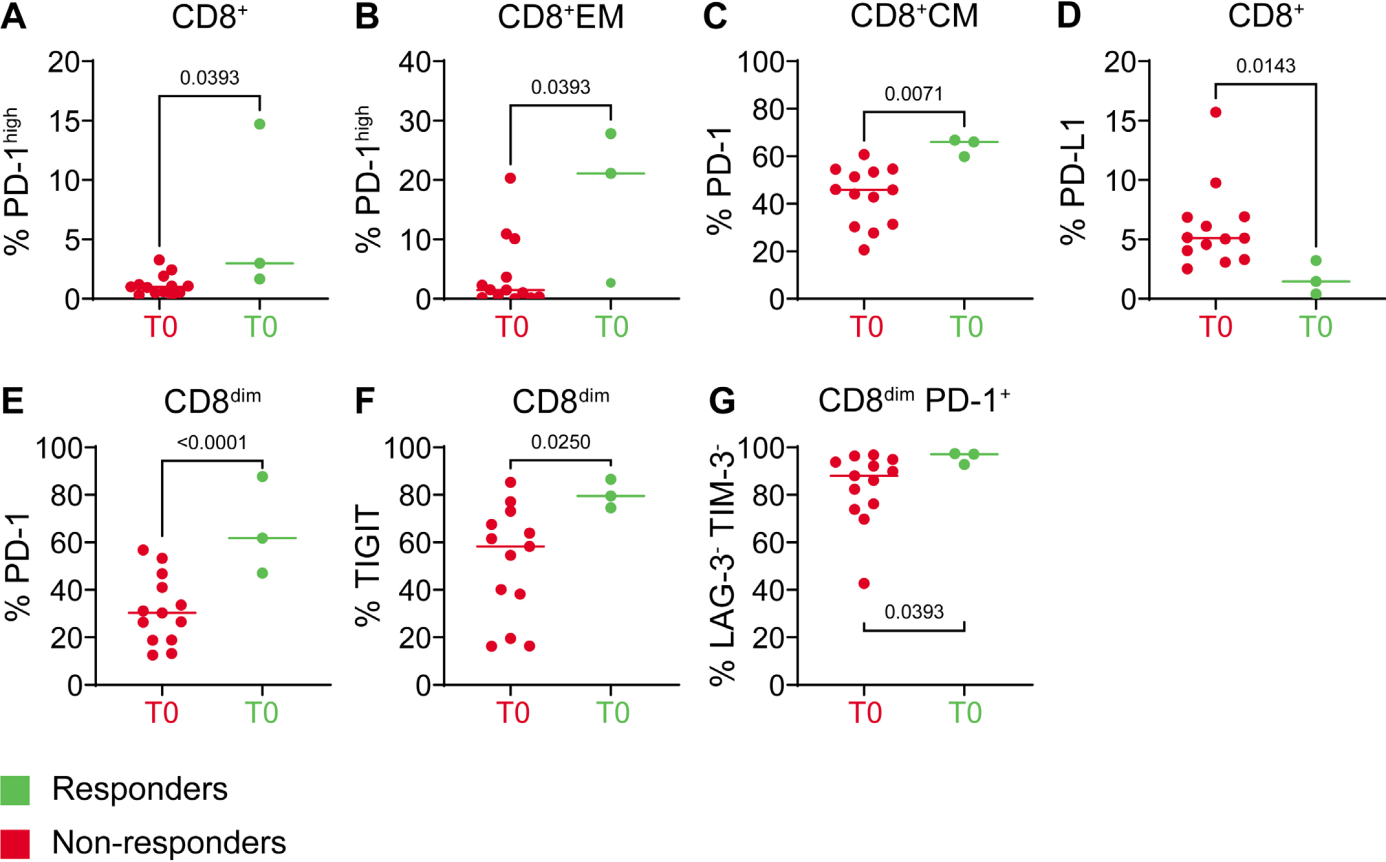
Baseline significant differences in CD8^+^ T cell checkpoint expression between responders and non-responders. Graph title depicts subset, y-axis depicts percentage of cells in subset expressing given checkpoint.

At first, we set out to assess baseline differences between responders (MPR) and non-pathological responders to identify putative predictive biomarkers. Of note, due to the low number of responders (n=3), observed differences were often not or borderline significant, warranting caution in overinterpretation. We did neither observe pre-treatment differences between responders and non-responders in the frequencies of CD4^+^ and CD8^+^ populations, nor in the distribution of the naïve, central memory (CM), effector or effector-memory (EM) subsets of these populations. Likewise, pre-treatment frequencies of NK cells and Tregs did not differ significantly (online supplemental figure S3). We did observe an overall higher activation state of CD8^+^ T cells at baseline in patients with MPR, evidenced by elevated expression levels of the immune checkpoints PD-1 and TIGIT; these differences reached significance for PD-1^high^ in the total CD8^+^ and CD8^+^ EM populations and for total PD-1^+^ in the CD8^+^ CM population, (figure 6A–C, online supplemental figure S4).^17^ Simultaneously lower expression levels of PD-L1 in the total CD8^+^ T cell population (figure 6D) indicated a decreased capacity for immune suppression, as CD8^+^ T cells with high PD-L1 expression have previously been reported to mediate T cell suppression in cancer and are related to a poor prognosis in ICI-treated patients.^18 19^

Of interest, these differences were more pronounced in a small subset of CD8^+^ T cells with low CD8 expression levels (CD8^dim^), with significant differences between responders and non-responders for total and intermediate expression levels of PD-1 and of TIGIT, suggestive of a progenitor exhaustion state that might be amenable to PD-1 blockade (figure 6E–G).^20^ Although not significant, this CD8^dim^ T cell population in responders appeared to comprise lower frequencies of naïve or senescent (EMRA) T cells and slightly elevated levels of CM cells.

**Figure 7:**
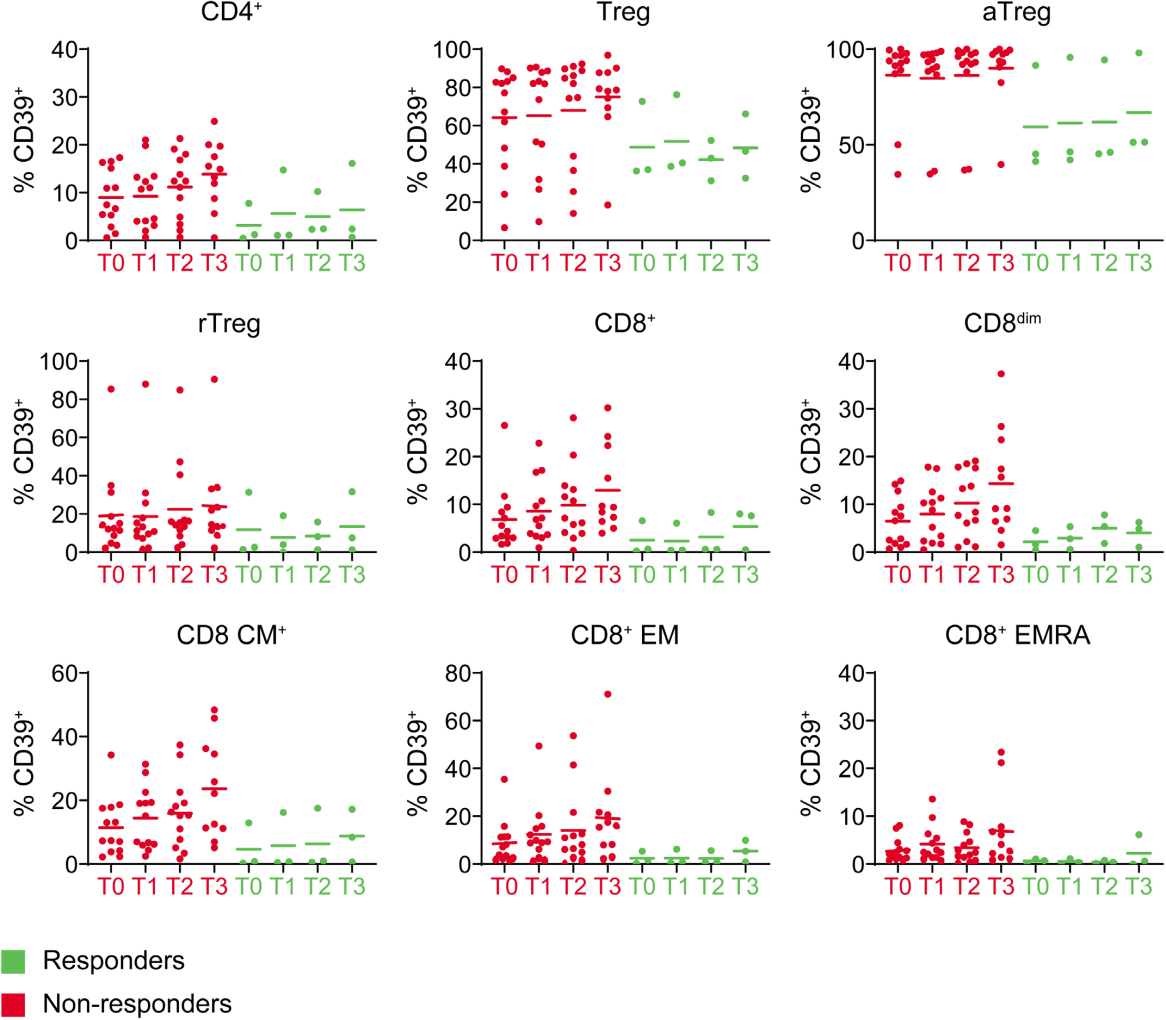
CD39 expression for all time points for the CD4^+^, Treg and CD8^+^ T cell subsets in responders versus non-responders. Graph title depicts T cell subset. CM, central memory; EM, effector memory; EMRA, effector memory cells re-expressing CD45RA.

Expression of activation markers LAG-3 and TIM-3 in any of the subsets did not differ significantly between responders and non-responders, though in responders frequencies of these markers were very low, whereas there was much more variation in the frequencies of TIM-3 and LAG-3 in the non-responders (online supplemental figures S5 and S6). For all CD8 populations, the majority of PD-1^+^ T cells lacked TIM-3 and LAG-3 expression. In responders, CD8^dim^ cells had significantly more LAG-3^−^ TIM-3^−^ PD-1^+^ cells compared with non-responders (figure 6G), with non-responders primarily having increased frequencies of PD-1^+^ TIM-3^+^ LAG-3^+/−^ cells (online supplemental figure S8). These data suggest a more profound state of exhaustion in the CD8^dim^ T cell population in non-responders. Higher baseline CD39 frequencies in non-responders on all T cell populations, including (activated) Tregs, clearly suggested a more immune suppressive state due to conversion of suppressive adenosine in these patients (figure 7).^21^ An overview of relative immune checkpoint expression levels between the other subsets according to response is shown in online supplemental figure S4 and detailed in online supplemental figures S5–S8.

To establish differences in effector T cell functionality between response groups, we determined ex vivo intracellular cytokine/granzyme expression levels and found elevated IFNγ levels in both CD4^+^ and CD8^+^ T cells in responders at baseline as well as lower IL-5 levels in the CD4/CD8 double positive (DP) subset (online supplemental figure S10). The latter fits with previous reports, identifying CD4/CD8 DP T cells as tumor-reactive effector cells with a propensity for Th2 skewing in cancer.^22 23^

#### Post-nivolumab peripheral T cell and NK cell subset frequencies and activation state

Expression of CD39, known for its immunosuppressive effect through the release of adenosine in the tumor microenvironment (TME), on CD4^+^, CD8^+^ and Treg subsets was consistently higher at all time points in non-responders compared with responders and appeared to further increase on treatment on CD8 populations, although this did not reach significance (figure 7). As expected due to the presence of nivolumab binding to the PD-1 epitope, PD-1 staining on both CD4^+^ and CD8^+^ T cells declined after nivolumab therapy in both responders and non-responders (online supplemental figures S4–S8).

A post-treatment increase in Ki-67 expression on both CD4^+^ and CD8^+^ T cells showed increased activation and proliferation of T cells by PD-1 blockade, without any relation to response (online supplemental figures S5–S7). In CD8^+^ and CD8^dim^ T cells, we observed a lower TIM-3 and higher TIGIT expression over all time points in the responders (online supplemental figures S6 and S7). Whereas Treg frequencies were quite similar in responders and non-responders over the time points, we observed a marked increase in LAG-3^+^ activated Treg on T=3 in the responders (online supplemental figure S9).

We did not observe any significant changes over time in cytokine/granzyme-B expression patterns per T cell subset, nor any differences in this respect between response groups (online supplemental figure S10). Finally, no significant post-treatment differences were seen in NK cell frequencies and activation markers between response groups.

#### Pre- and post-nivolumab myeloid subset frequencies and activation state

In non-responders, comparing baseline to T=3, there was a significant drop in peripheral B cell frequencies (p=0.010), and an increase in classical (p=0.033) and non-classical monocytes (p=0.019). Similar trends were observed in responders, though these did not reach significance due to smaller numbers (online supplemental figure S11). cDC1, often referred to as the dendritic cell subset with most potent cross-presenting abilities, had elevated frequencies of CD40 at all time points in responders, and in two out of three responders induction of cDC1 activation 1 week after nivolumab administration (T=1) was observed, as indicated by CD80 and PD-L1 upregulation (online supplemental figure S12). We did not observe baseline or post-nivolumab differences between response groups in the frequencies of classical, intermediate and non-classical monocytes, type 1 or 2 conventional dendritic cells (cDCs), plasmacytoid DCs (pDC), B cells, monocytic myeloid-derived suppressor cells (mMDSCs) or early MDSCs (eMDSCs) (online supplemental figure S3).

### RNA sequencing

**Figure 8:**
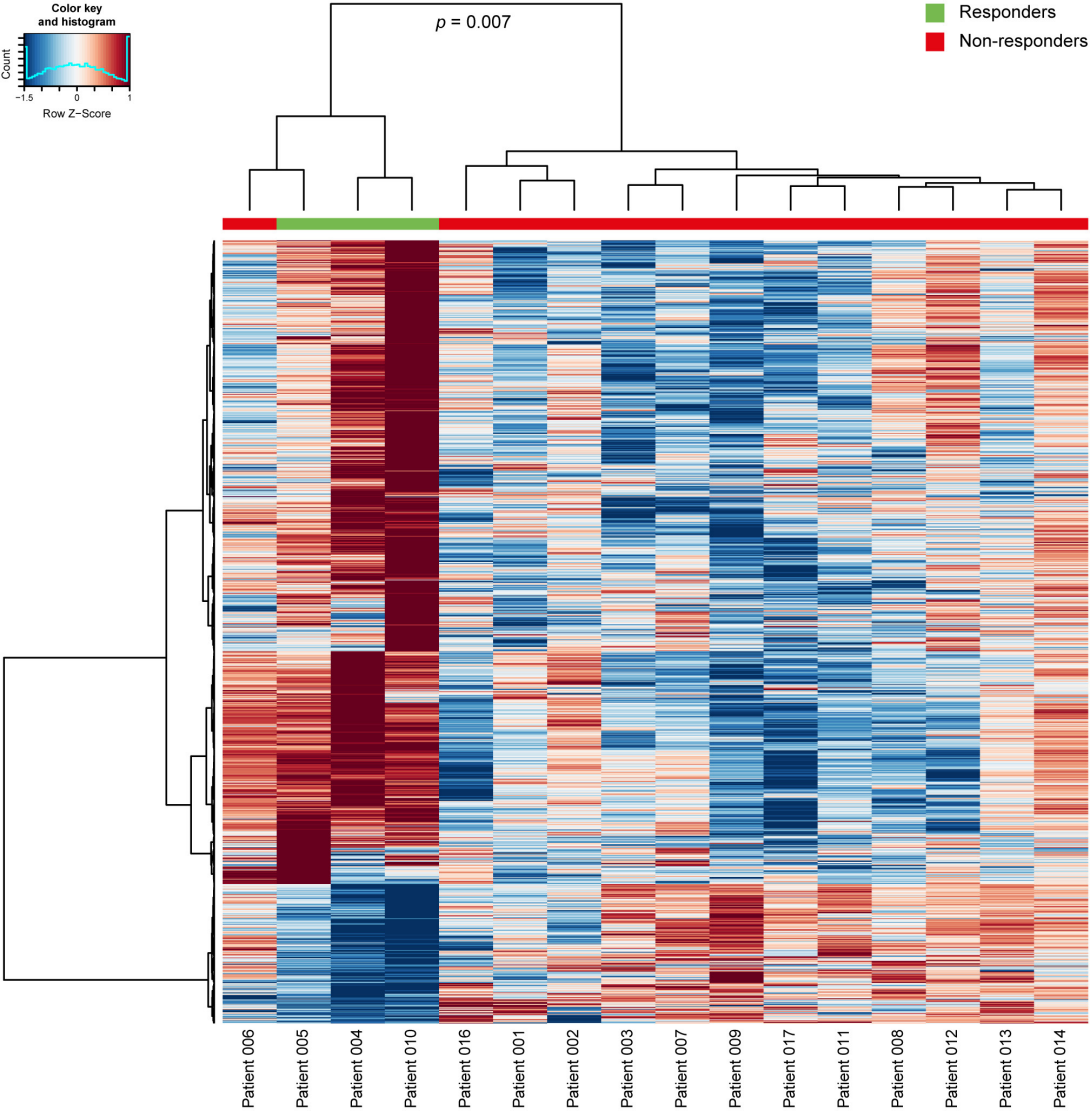
Heatmap of 1235 differentially expressed RNA transcripts (FDR <0.05) between responders (n=3) and non-responders (n=13) at baseline. The columns indicate samples, the rows indicate RNA transcripts. Z-score transformed expression values are represented by colors: red represents high and blue represents low expression. Unsupervised hierarchical clustering showed significant clustering of a subgroup of all responders and one non-responder vs 12 non-responders (p=0.007). FDR, false discovery rate.

From all 16 patients, a pretreatment tumor biopsy was available for RNA sequencing analyses, all of which were successfully RNA sequenced. Analysis of variance (ANOVA) differential expression analysis showed 1235 genes were differentially expressed (false discovery rate <0.05) between responders and non-responders, of which 1015 were downregulated and 220 were upregulated in responders relative to non-responders. Unsupervised hierarchical clustering of all differentially expressed genes distinguished all patients with MPR from those without MPR, with only non-responding patient 006 clustering to the responding patients (p=0.007; figure 8). STRING analysis of differentially expressed transcripts showed enrichment for a wide variety of 847 biological processes (39 and 808 among upregulated and downregulated transcripts in responders vs non-responders, respectively).

## Discussion/conclusion

Neoadjuvant nivolumab induced rapid pathological responses in patients with oral cancer given as a single flat dose of 480 mg ~3 weeks prior to extensive surgery. MPR (≤10% RVT) were seen in 3 of 16 (19%) patients, which is somewhat higher than previous reports on neoadjuvant anti-PD-1 treatment in HNSCC: mean 9.7% (range 2.9%–17%).^24^ Treatment was feasible and did not delay standard-of-care procedures.

Responders showed a favorable survival, with a 3-year DSS of 100% compared with 85% in non-responders. Rogers *et al* have investigated the survival of 541 patients with stage III/IV oral cancer treated with primary surgery and found a 3-year DSS of ~60%–80%.^25^ Although our findings were not statistically significant, it is encouraging that none of the responders experienced disease recurrence in this time frame.

The AE profile was remarkably mild, only grade I/II AEs were observed, most of which could possibly have been due to standard-of-care treatment, that is, surgery. Vos *et al* have shown that neoadjuvant ICI combination therapy induces higher MPR rates compared to single-agent neoadjuvant nivolumab (35% vs 17%), with a grade III–IV toxicity rate of 38%.^11^

PET imaging using the anti-PD-L1 adnectin tracer ^18^F-BMS-986192 has shown promise for producing potential predictive biomarkers in non-small cell lung cancer and melanoma.^13 14^ In this study, baseline SUV_peak_ of ^18^F-BMS-986192 showed a trend to be higher in patients with versus without a MPR (median SUV_peak_ 5.3 vs 3.4). Similarly, Niemeijer *et al* found a numeric difference in SUV_peak_ between responders and non-responders (median SUVpeak 6.5 vs 3.2) in patients with lung cancer. The interpatient variability in both studies is considerable, complicating the interpretation of its potential as a predictive biomarker in studies with limited power. An ongoing study in patients with lung cancer including up to 80 subjects (NCT03564197) may provide further insights in this matter.

Furthermore, uptake of ^18^F-BMS-986192 by tumor cells cannot be discerned from uptake by immune cells in the TME. PD-L1 expression on immune cells can be interpreted as either proinflammatory (such as PD-L1 expression on activated dendritic cells) or anti-inflammatory (such as PD-L1 expression on myeloid suppressor cells), potentially clarifying the ambiguous results on treatment: a notable decrease in ^18^F-BMS-986192 uptake was found in two out of three major responders on treatment, but the third major responder conversely demonstrated an increase in ^18^F-BMS-986192 uptake post-treatment. Presumably, the first two responders had such a drastic decrease in PD-L1 positive tumor cells (both less than 1% RVT in the resection specimen<1 week after the scan; TPS 50%–70%) that this resulted in a net decrease, whereas the latter patient had a PD-L1 negative tumor, and the ^18^F-BMS-986192 uptake might have been driven by an active immune infiltrate, resulting in a net increase. Further research is needed to clarify the exact source of ^18^F-BMS-986192 uptake to assess its value in response prediction.

Finally, based on our data, a decrease in TLG of ^18^F-FDG of >20% is a helpful cut-off for recognizing major and partial pathological responses, as was also previously shown by Vos *et al*.^26^ In future studies, this early response marker could be employed to identify patients benefiting from neoadjuvant anti-PD-1 treatment.

Flow-cytometric PBMC analyses revealed higher baseline PD-1 levels on CD8^+^ and CD8^dim^ T cells in responders. PD-1 levels on CD4^+^ T cells were not significantly different. Especially given the lack of robustness of pre-treatment PD-L1 CPS to predict response, also in this study, these data warrant further investigation into PBMC PD-1 expression levels as a potential biomarker for selecting patients who will likely respond to ICIs. In melanoma, circulating PD-1^+^ TIGIT^+^ CD8^+^ T cells present 1 month after anti-PD-1 therapy have been linked with response to anti-PD-1 therapy.^27^ In our study, we already observed elevated frequencies of these cells in responders at baseline, primarily detected as CD8^dim^ cells. At later time points our monitoring did not allow for PD-1 detection due to binding of nivolumab to the same epitope as the flow phenotyping antibody, but high levels of TIGIT remained stable over the course of treatment on the CD8^dim^ population. Our identification of a CD8^dim^ T cell population, with a phenotype consistent with a progenitor exhausted state and carrying baseline features with a potentially predictive value, is a novel finding. A similar CD8^dim^ effector T cell subset was previously identified in patients with OSCC undergoing nivolumab treatment.^28^ Further phenotypic and functional characterization of this population (eg, does it represent activated CD8^+^ T cells or is it an actual separate T cell subset?) is certainly warranted in follow-up studies.

While we did not observe a difference in Tregs between responders and non-responders, non-responders had higher CD39 frequencies on all T cell populations which might suggest a difference in conversion of suppressive adenosine in these patients.^21^ PD-L1 expressing CD8^+^ T cells, which were significantly higher at baseline in non-responding patients, have in lung cancer been reported to be suppressive and in melanoma were found to be related to a poor prognosis in ICI-treated patients.^18 19^ Counterintuitively, in our study, induction of LAG-3 was observed on Tregs in patients with a major response at the follow-up blood draw. Interestingly, in autoimmune diabetes, Zhang *et al* reported LAG-3 on Tregs to limit their proliferation and function.^29^ Induction of LAG-3 in this setting could, therefore, be beneficial. It also raises the question whether addition of a LAG-3 inhibitor to neoadjuvant anti-PD-1 in this setting might hamper rather than favor antitumor responses. Though limited by small numbers of patients, functional analysis of peripheral T cells after in vitro (antigen-independent) stimulation showed already at baseline higher intracellular expression levels of cytotoxic effector molecules like IFNγ and granzyme-B from CD8^+^ T cells of responders. It has been postulated that production of IFNγ by cytotoxic T cells improves their cytotoxic potential as well as their mobility, which could suggest that the cytotoxic fitness of the CD8^+^ T cells was better at baseline in responders.^30^ Together, the immune monitoring data in this trial suggest that non-responding patients had a more suppressed peripheral immune phenotype at baseline, whereas T cells in responders had less suppressive features and a more activated profile.

A 19% MPR rate on a single dose of nivolumab is highly promising and combined with other studies advocates for anti-PD-1 therapy in the neoadjuvant curative setting prior to surgery for locally advanced HNSCC.^11^ Prolonging nivolumab treatment with multiple doses might induce higher response rates but the narrow time frame from diagnosis to standard of care treatment does not allow for an extensive neoadjuvant treatment regimen. In future studies, facilitating extension of this time frame by delaying surgery could be considered in responders, once again underlining the need for robust biomarkers for response. One could also opt for prolonging immunotherapy until after surgery. The HNSCC field awaits the results from the KEYNOTE-689 randomized controlled trial using pembrolizumab in the neoadjuvant setting combined with surgery and adjuvant pembrolizumab.^31^ The question remains whether adjuvant treatment has additional effects. A third potential approach to improve response rates is to combine ICIs, which has already shown promising results.^8 9 11^

A limitation for our current study, trying to find indicators of response, is that due to the small trial set-up, only three patients achieved an MPR and this made the responder group rather small for achieving statistically significant differences for both the molecular imaging and peripheral immune monitoring. Still, several interesting observations were made that should be validated in other cohorts or new trials exploiting immunotherapy in the window-of-opportunity between diagnosis and surgery.

## Methods

### Patients

Patients with locally advanced stage III/IV OSCC scheduled for surgery with curative intent were eligible for this study. Patients were staged according to the AJCC eighth edition. Other relevant inclusion criteria were age ≥18 years, ECOG performance status of 0 or 1, adequate organ function as demonstrated by standard blood work-up (including white cell count, absolute neutrophil count, platelets, hemoglobin, creatinine, total bilirubin, aspartate transaminase and alanine transaminase). Exclusion criteria were a current additional malignancy that required active treatment (excluding basal cell carcinoma of the skin, squamous cell carcinoma of the skin or in situ cervical cancer treated for cure), inadequate recovery from any previous major surgery, requiring systemic treatment with corticosteroids (10 mg daily prednisone equivalent) or other immunosuppressive medication within 14 days of screening (excluding inhaled or topical steroids and adrenal replacement steroids in the absence of autoimmune disease), active autoimmune disease or a history of a clinically severe autoimmune disease, evidence of interstitial lung disease, evidence of active infection including SARS-CoV-2, previous treatment with antibodies targeting immune checkpoints or T cell costimulation, known hepatitis B or C infection, or known inadequately treated HIV infection (viral load >50 copies/mL).

### Study design

The study design is depicted schematically in online supplemental figure S10 and the study protocol is available in online supplemental file 2. Standard-of-care clinical workup at time of first presentation included a tumor biopsy for definitive histopathological diagnosis, baseline MRI or CT scan, baseline FDG PET scan, orthopantomogram, ultrasound assessment of cervical lymph nodes with fine needle aspiration and tumor examination under general anesthesia. In a multidisciplinary meeting the staging and definitive treatment plan was determined, after which patients could be enrolled in the current study. As part of study procedures a baseline ^18^F-BMS-986192 (PD-L1) PET scan was made and the primary tumor was tattooed with a 5 mm margin to secure adequate surgical margins in anticipation of any volume changes as a result of neoadjuvant treatment. Next, patients were administered a single flat dose of 480 mg nivolumab intravenously according to standard procedures. Blood samples for safety and immune monitoring were drawn at baseline (T=0), 7 days post-nivolumab (T=1), 2 weeks post-nivolumab (T=2) and 3 months post-surgery (T=3). Three weeks after treatment with nivolumab, imaging procedures were repeated including a post-nivolumab PD-L1 PET, FDG PET and MRI or CT scan. Patients underwent surgical resection of the primary tumor and cervical lymph nodes at approximately 3 weeks post-nivolumab. Patients received adjuvant treatment according to institutional guidelines and the Dutch Head and Neck Cancer Cooperative Group guidelines. In case of a pre-treatment indication for postoperative adjuvant treatment, this was not reconsidered as a result of pathological downstaging in patients with a response to neoadjuvant nivolumab. All patients were monitored for AEs until 100 days after nivolumab treatment. AEs were graded according to the Common Terminology Criteria for Adverse Events v5.0. Standard-of-care follow-up visits were conducted every 3 months until progression, death, or loss to follow-up.

### Pathological assessment

Pathological response classification was adapted from the immune-related pathological response criteria: MPR was defined as ≥90% response (≤10% residual volume of viable tumor (RVT)), partial pathological response (PPR) as ≥50%–90% and <50% response (>50% RVT) as no response.^32^ Patients with MPR were considered responders.

PD-L1 staining was performed using an optimized laboratory-developed test (Dako/Agilent 22C3 pharmDX) and scored using the TPS and CPS.^33 34^

### ^18^F-anti-PD-L1 PET/CT (PD-L1 PET)

The anti-PD-L1 PET tracer ^18^F-BMS-986192 was synthesized at the GMP lab of the Department of Radiology and Nuclear Medicine of Amsterdam UMC, location Vrije Universiteit according to GMP guidelines.^35^ To dried ^18^F-fluoride a solution of BMT-180478 in DMSO was added. The reaction vessel was heated for 10 min at 120°C. The reaction mixture was diluted with H_2_O and purified over a Phenomenex Luna C18(2) 5 µm 250×10 HPLC column. The collected ^18^F-BMT-187144 was trapped on a solid phase extraction (SPE) cartridge and eluted into the second reaction vessel with ethanol after which it was evaporated to dryness. At 45 °C ^18^F-BMT-187144 was reacted with BMT-192920 for 45 min in PBS to form the final compound. The crude reaction mixture was purified by size exclusion using a PD-10 desalting column and PBS. The collected fraction containing the ^18^F-BMS-986192 was sterile filtrated and dispensed using reduced pressure. Patients received an intravenous bolus injection of ^18^F-BMS-986192 of 216 MBq (range 167–246), followed by PET/CT scan 60–75 min postinjection (10–12 bed positions, each 3 min, head to mid-thighs). PET/CT scans were acquired on a Philips Ingenuity TF PET/CT scanner or a Philips Vereos PET/CT scanner (Philips Medical Systems, Best, The Netherlands).

### ^18^F-fluorodeoxyglucose positron emission tomography/CT

^18^F-fluorodeoxyglucose positron emission tomography/CT (^18^F-FDG PET/CT) scans were performed in compliance with European Association of Nuclear Medicine 2.0 guidelines.^36^ Patients underwent 6 hours of fasting prior to administration of 3 MBq/kg (±10%) ^18^F-FDG PET/CT scans were made approximately 60 min post injection (head to mid-thighs).

### PET image analysis

For the primary tumor lesions, volumes of interest were drawn semiautomatically on the PET images by applying a semiautomated majority vote approach—segmentation defined by consensus at voxel level of at least three of four standard threshold-based methods, which was adjusted manually only if visually misaligned using in-house-developed software (ACCURATE tool).^37 38^ All primary tumors had a diameter >10 mm. For analyses, SUV_peak_ values and TLG or TLA were used (TLG for ^18^F-FDG PET and TLA for ^18^F-BMS-986192 PET). Uptake in tumor lesions was non-parametric and therefore expressed as median with IQR.

### Flow cytometry

Peripheral blood for immune monitoring was drawn in four 10 mL heparinized collection tubes at time points T=0, 1, 2 and 3. Immediately after blood draw PBMCs were isolated by Lymphoprep (STEMCELL Technologies) density gradient centrifugation and cryopreserved for later analysis as previously described.^39^ All PBMC samples were subjected to multiparametric flow cytometry analyses to compare the frequencies and activation state of lymphocytic and myeloid subsets as described previously.^40 41^ Comparisons were made between responders and non-responders at T=0 to identify possible baseline biomarkers for response. To compare changes over time in the various PBMC subsets in relation to nivolumab therapy, we compared the frequencies and marker expression at all four time points for both responders and non-responders.

Additionally, intracellular cytokine staining was carried out on PBMC samples from all time points from three responders (patients 004, 005, 010) and three non-reponders (patients 003, 012, 013). For this, the cells were incubated with 50 ng/mL PMA and 1 µg/mL ionomycin for 1 hour, after which Golgistop was added and cells were incubated for an additional 4 hours. After this, cells were harvested and stained for surface T cells antibodies (CD3, CD4, CD8). Next, cells were permeabilized using the BD Fixation/Permeabilization Solution Kit (BD Biosciences), stained for IL-5, granzyme B and IFN-y and subsequently subjected to flow cytometry analysis. One sample (patient 004, T=1) was excluded due to low viability of the cells. For a list of all used monoclonal antibodies, see online supplemental table S2.

### RNAseq

Tumor RNA was isolated from snap-frozen pretreatment tumor biopsies acquired at baseline. Up to 20 cryosections were allocated per isolation depending on biopsy size and tumor percentage. The first and last cryosections were HE stained and reviewed by a pathologist to ensure a minimal tumor percentage of 20%. Subsequently, RNA was isolated using the Purelink RNA mini kit (Invitrogen). RNA sequencing libraries were prepared using the NEBNext Ultra II RNA Library Prep Kit for Illumina following manufacturer’s instructions (NEB, Ipswich, Massachusetts, USA). Briefly, mRNAs were first enriched with Oligo(dT) beads. Enriched mRNAs were fragmented for 15 min at 94°C. First-strand and second-strand cDNAs were subsequently synthesized. cDNA fragments were end-repaired and adenylated at 3’ends, and universal adapters were ligated to cDNA fragments, followed by index addition and library enrichment by limited-cycle PCR. Libraries were sequenced on a NovaSeq 6000 platform. The raw RNA sequencing data were cleaned-up using Trimmomatic and mapped using STAR.^42 43^ SAMtools was used for converting files from .sam to .bam and data sorting.^44^ Subsequently, data were normalized by trimmed mean of M values (TMM), and remove unwanted variation (RUV) correction was used to correct for library size.^45 46^ To identify enrichment for biological processes among responders versus non-responders, we employed STRING analysis (www.string-db.org).^47^

### Statistics

For imaging, paired Wilcoxon tests were performed to assess differences between baseline and post-treatment SUV_peak_ levels.

For peripheral immune monitoring, differences between subset frequencies at baseline between responders and non-responders were assessed using an independent t-test or two-sample Mann-Whitney U test depending on normality. Differences between subset frequencies before, during and after nivolumab treatment were assessed using an ordinary one-sided repeated measures ANOVA or Kruskal Wallis test, depending on normality.

ANOVA differential expression analysis was performed to compare baseline RNA expression levels between responders and non-responders.

Statistical analyses were performed with GraphPad Prism (v9.1.0) and R (v4.0.3). P values <0.05 were considered statistically significant.

## supplementary material

10.1136/jitc-2024-009278 online supplemental file 1

10.1136/jitc-2024-009278 online supplemental file 2

## Data Availability

Data are available on reasonable request. All data relevant to the study are included in the article or uploaded as supplementary information.
