# Supplementary material for: Circulating T cell status and molecular imaging may predict clinical benefit of neoadjuvant PD-1 blockade in oral cancer
Source: J Immunother Cancer. 2024 Jul 22;12(7):e009278. doi: 10.1136/jitc-2024-009278 (PMC11268040; doi:10.1136/jitc-2024-009278)
Supplement: online supplemental file 1 [file jitc-12-7-s001.docx]

# Supplemental Materials


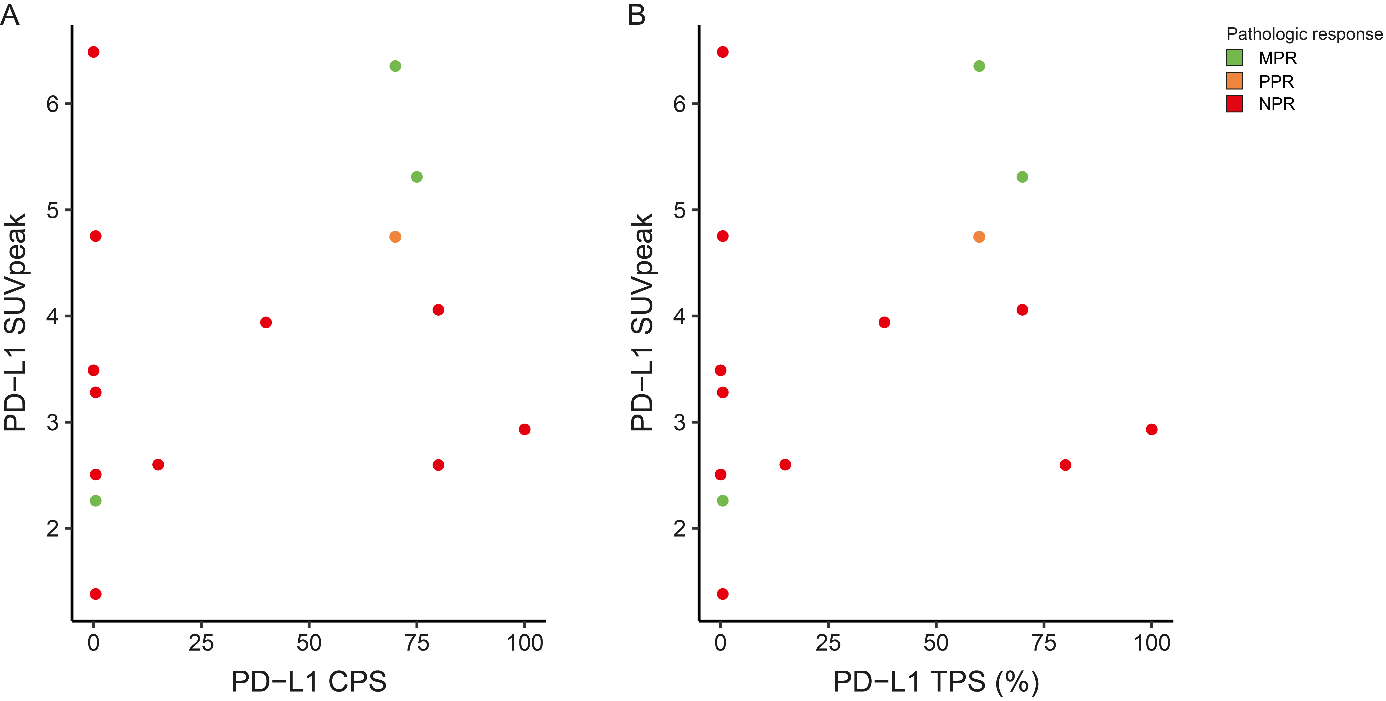


## Fig. S1 Correlation of PD-L1 CPS (A) and TPS (B) with uptake of ^18^F-BMS-986192. Uptake of ^18^F-BMS-986192 at baseline did not correspond to PD-L1 CPS or TPS in pre-treatment tumor biopsies. CPS = combined positivity score, TPS = tumor proportion score, SUV = standard uptake value, MPR = major pathologic response, PPR = partial pathologic response, NPR = no pathologic response.


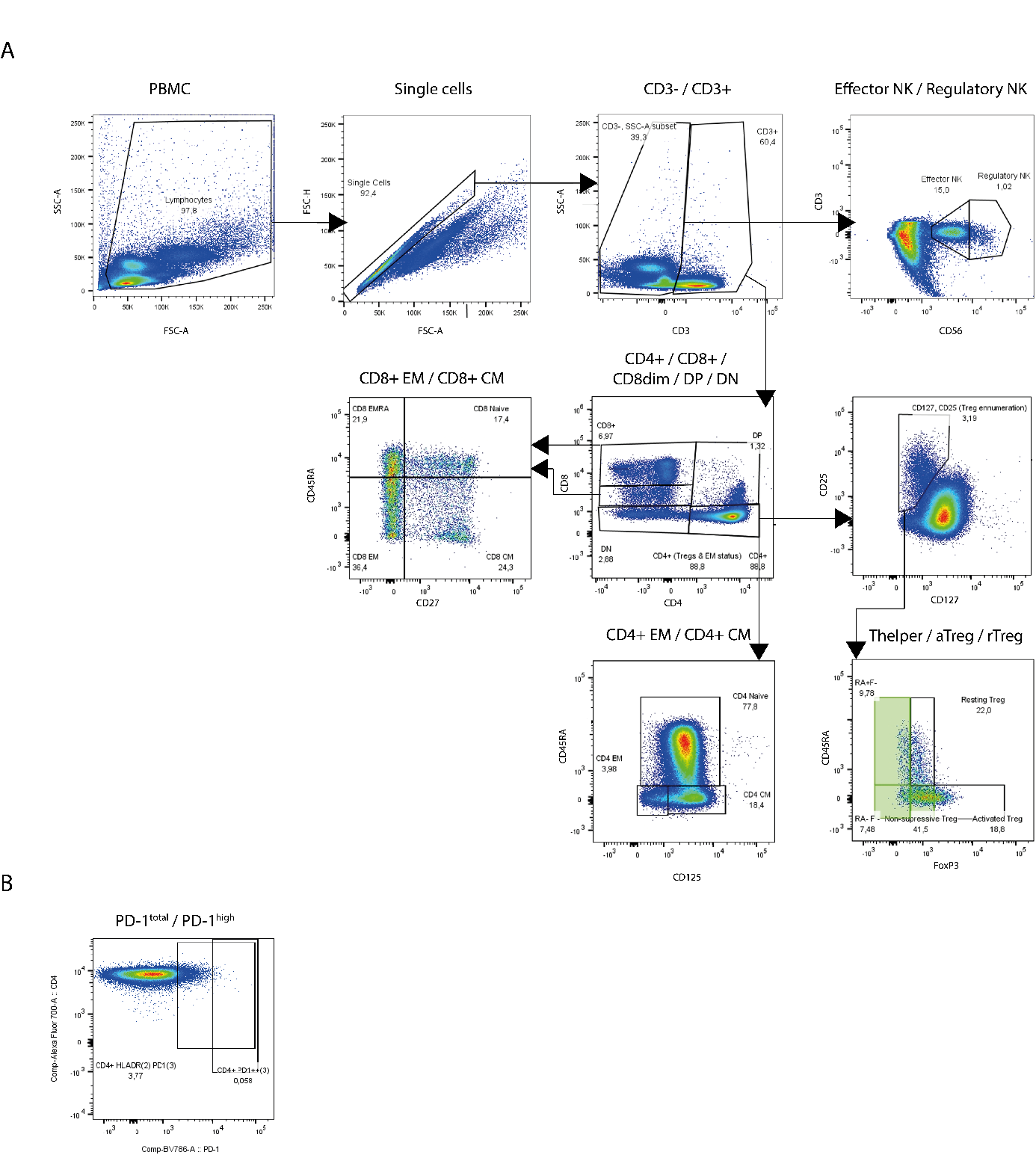


## Fig. S2 PBMC gating strategies.


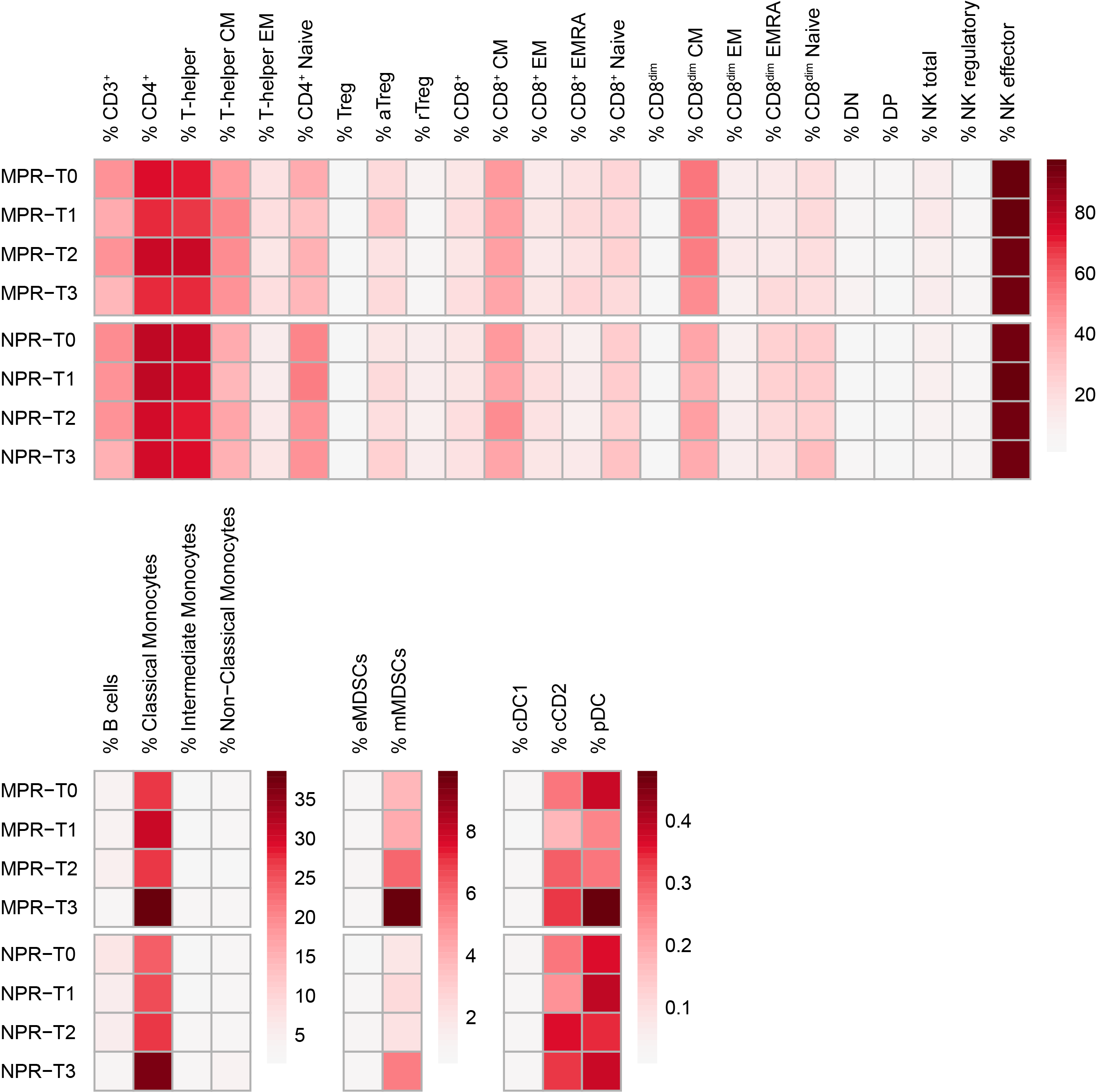


## Fig. S3 Overview of pre- and post-treatment PBMC subset frequencies in major pathological responders (MPR) and non-responders (NPR) at timepoints T0-T3. CM = central memory, EM = effector memory, EMRA = effector memory cells re-expressing CD45RA, Treg = regulatory T cel, aTreg = activated Treg, rTreg = resting Treg, DP = CD4/CD8 double positive, DN = CD4/CD8 double negative, NK = natural killer, eMDSC = early myeloid derived suppressor cells, mMDSC = monocytic MDSC, cDC1/2 = type 1/2 conventional dendritic cells, pDC = plasmacytoid DC.


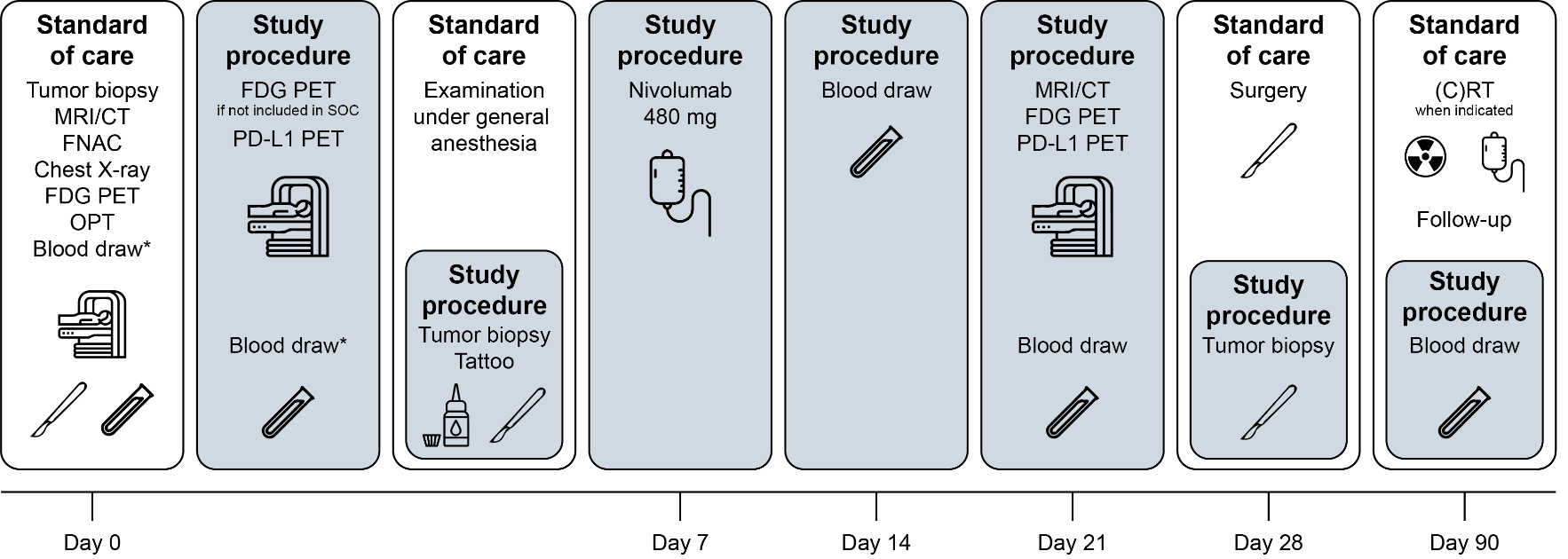

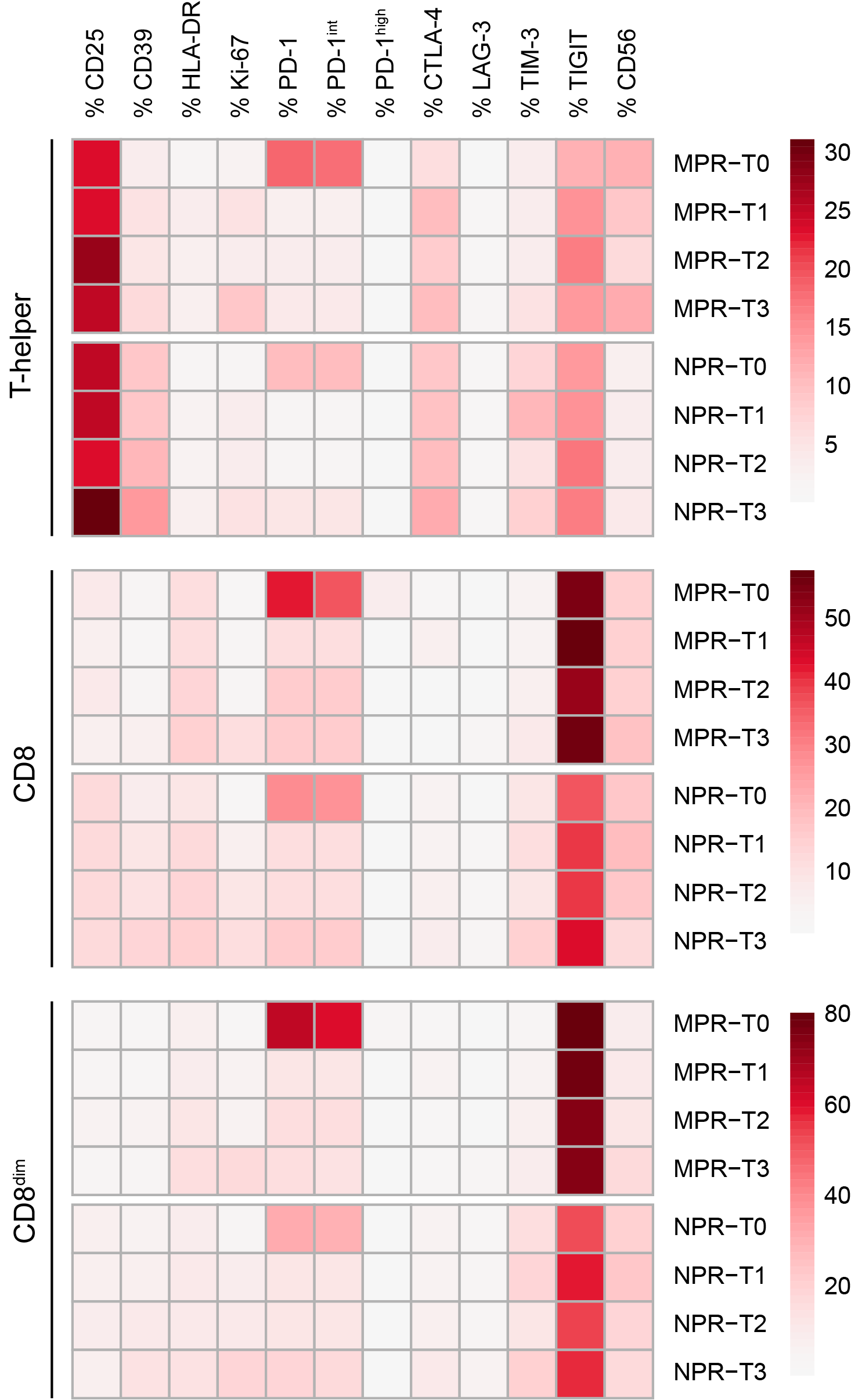


## Fig. S4 Pre- and post-treatment marker expression for CD4, CD8 and CD8^dim^ subsets in major pathological responders (MPR) vs. non-responders (NPR) at timepoints T0-T3.

**
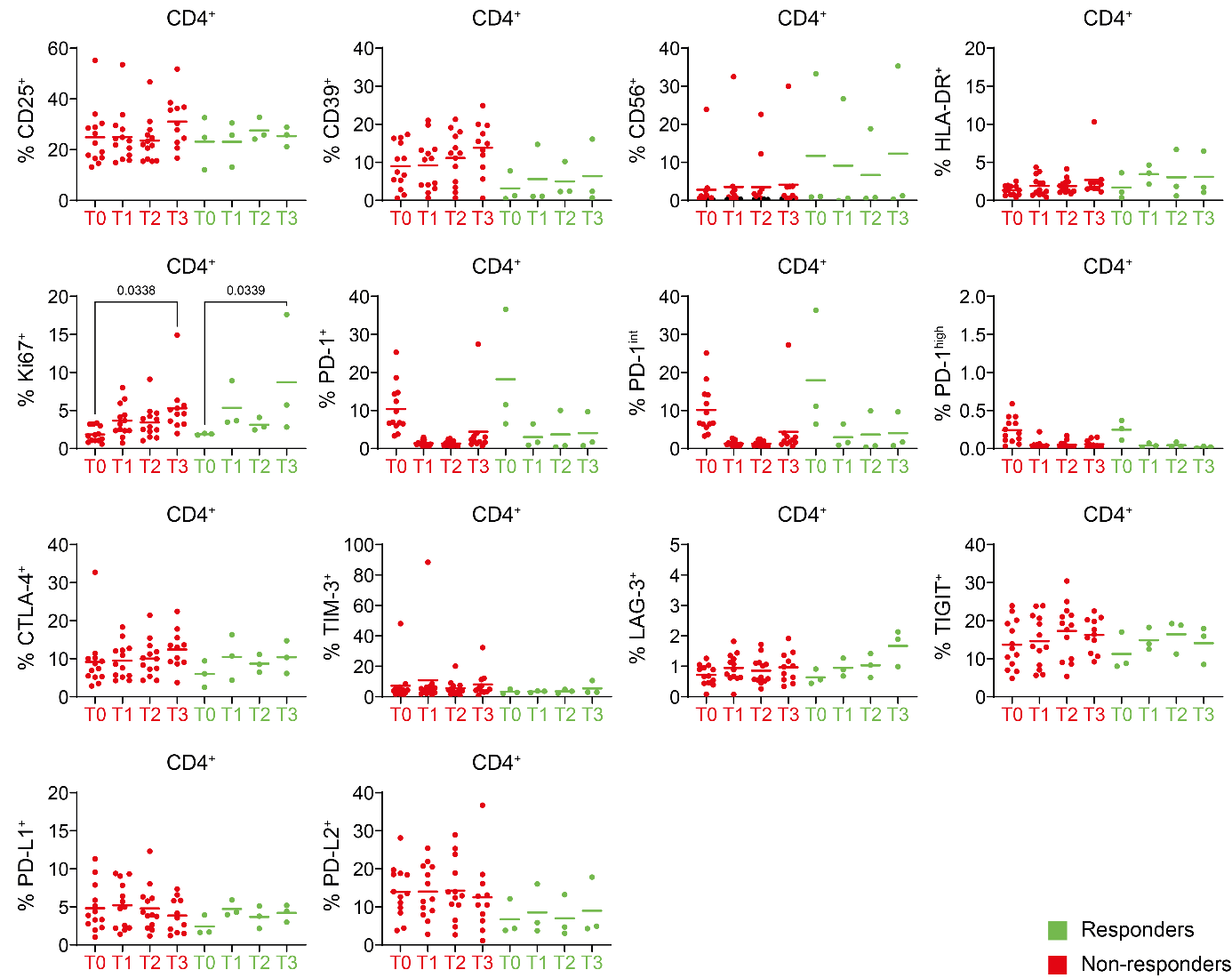
**

## Fig. S5 CD4+ T cell marker expression for all timepoints for responders vs. non-responders.

**
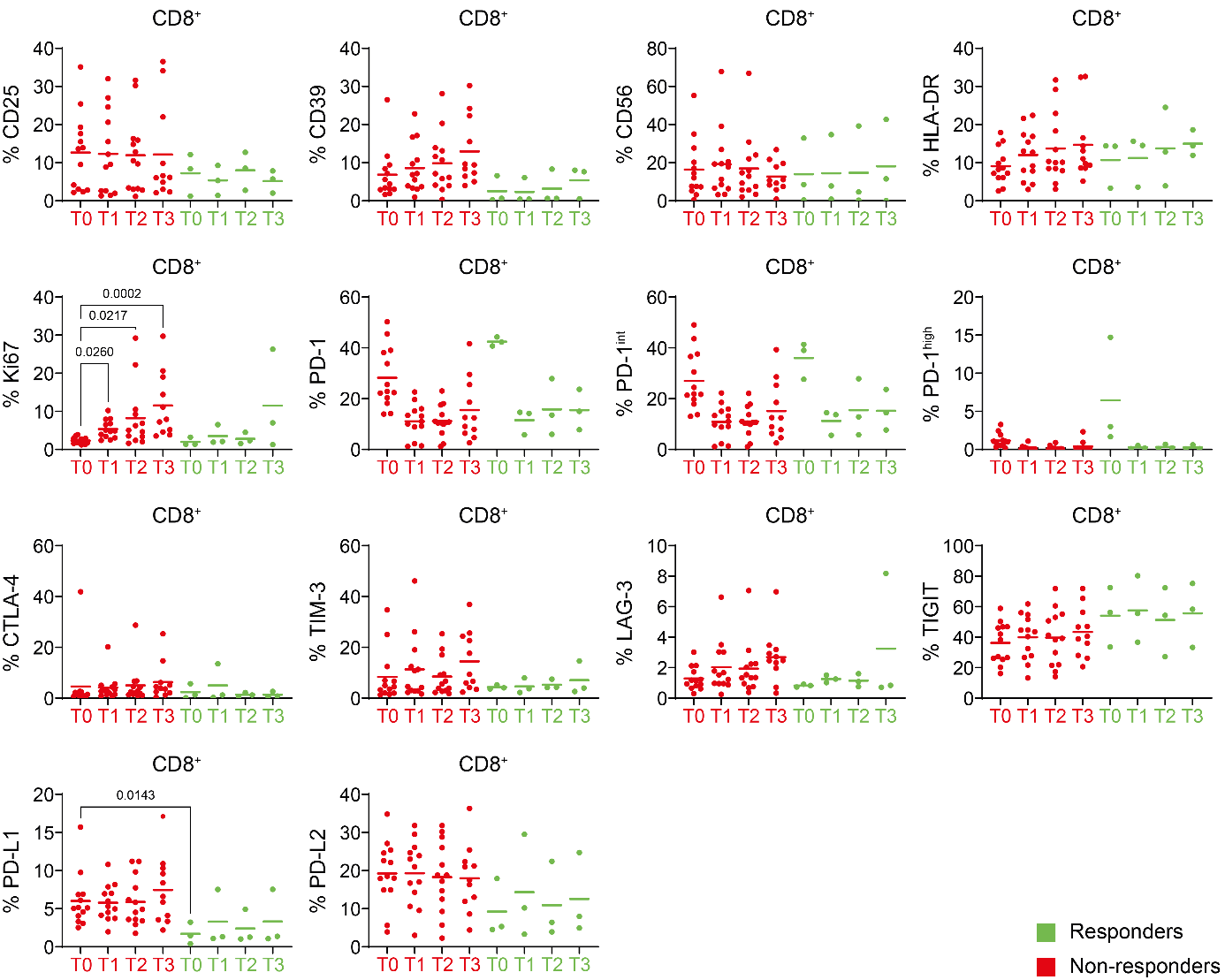
**

## Fig. S6 CD8^+^ T cell marker expression for all timepoints for responders vs. non-responders.

**
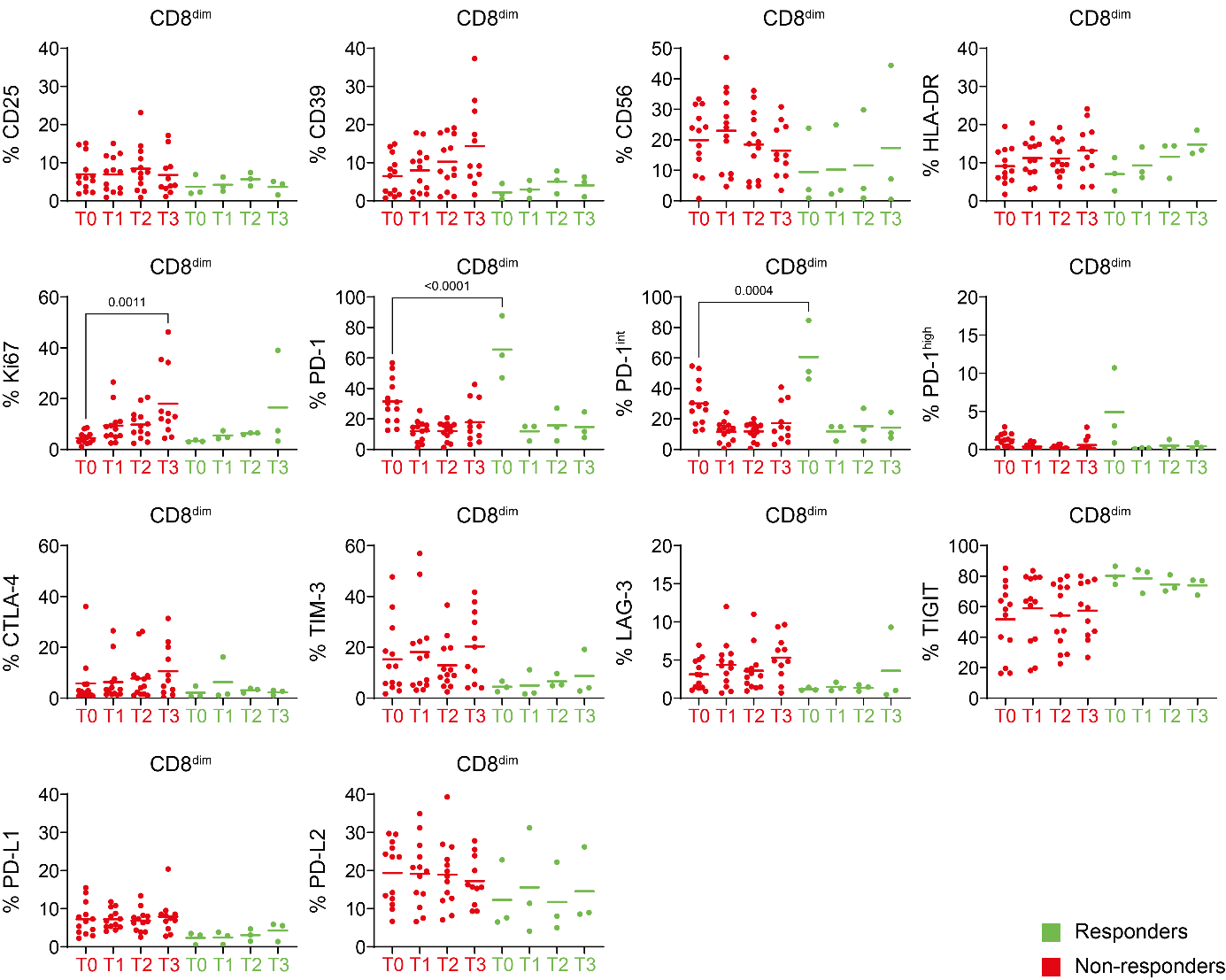
**

## Fig. S7 CD8^dim^ T cell marker expression for all timepoints for responders vs. non-responders.


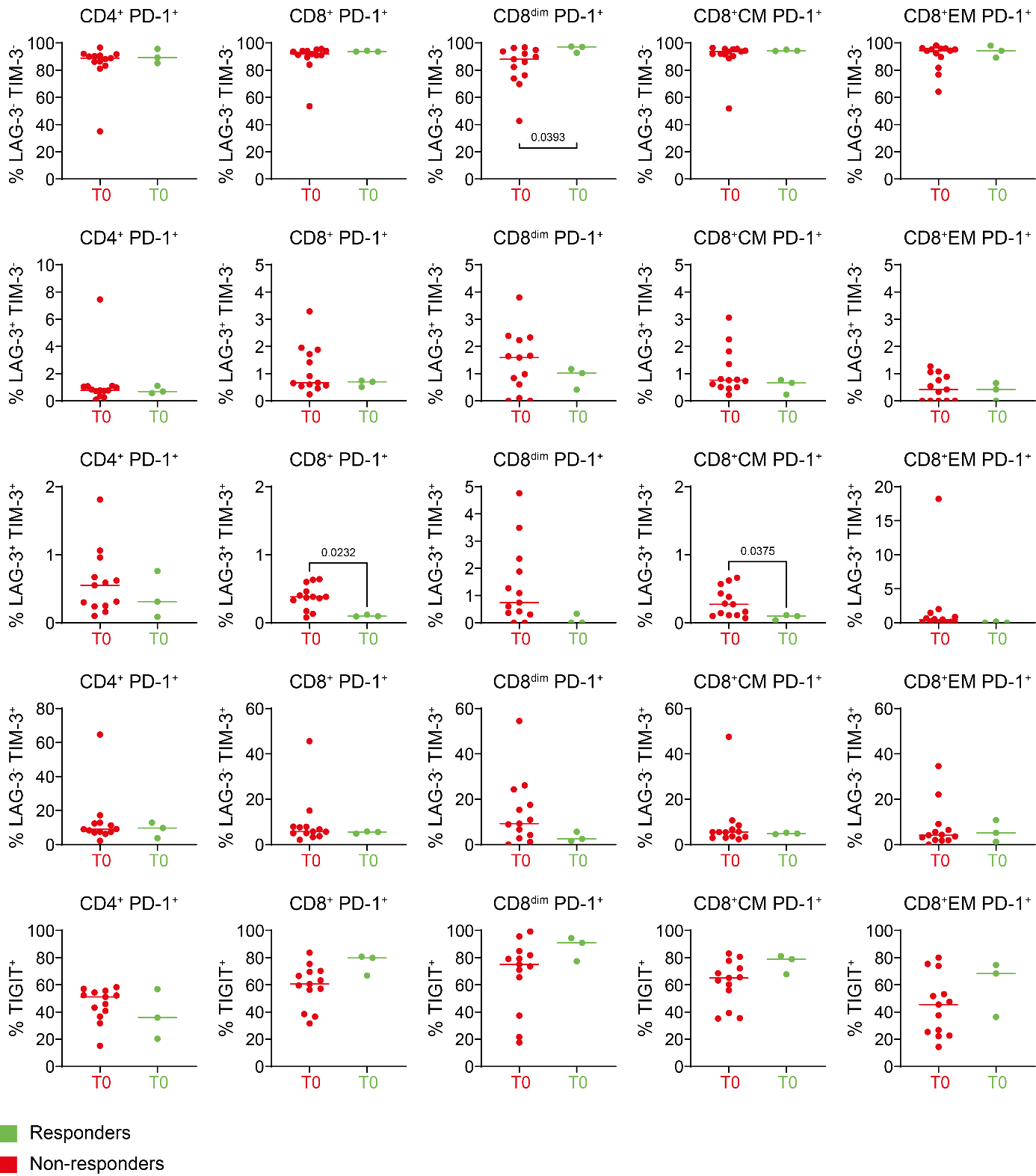


## Fig. S8 Baseline PD-1, LAG-3, TIM-3 and TIGIT co-expression for CD4^+^, CD8^+^, CD8^dim^, CD8^+^CM and CD8^+^EM T cell populations in responders vs. non-responders. CM = central memory, EM = effector memory.


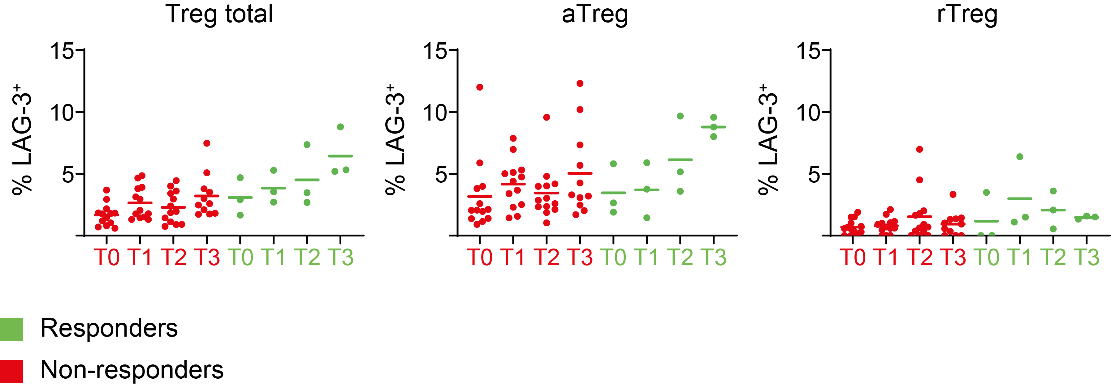


## Fig. S9 LAG-3 expression on Treg, aTreg and rTreg subsets for responders vs. non-responders. Treg = regulatory T cell, aTreg = activated Treg, rTreg = resting Treg.


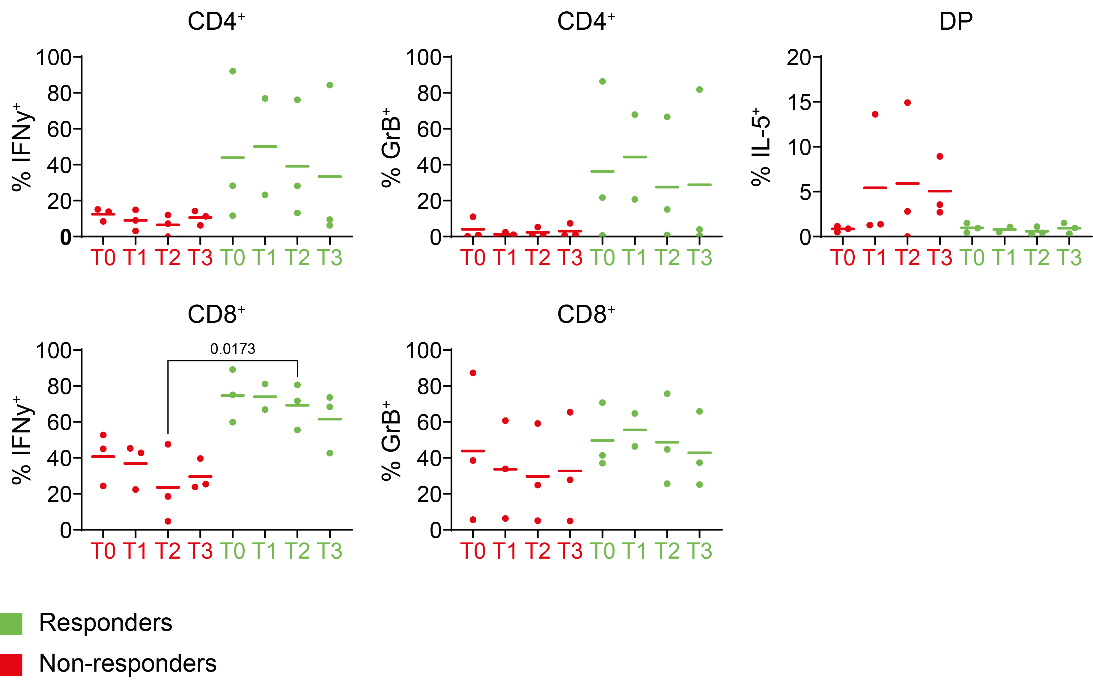


## Fig. S10 Intracellular IFNγ, granzyme B and IL-5 positivity for all timepoints for CD4^+^, CD8^+^ and double positive T cells in responders vs. non-responders. IFNγ = interferon gamma, GrB = granzyme B, IL-5 = interleukine 5, DP = double positive.


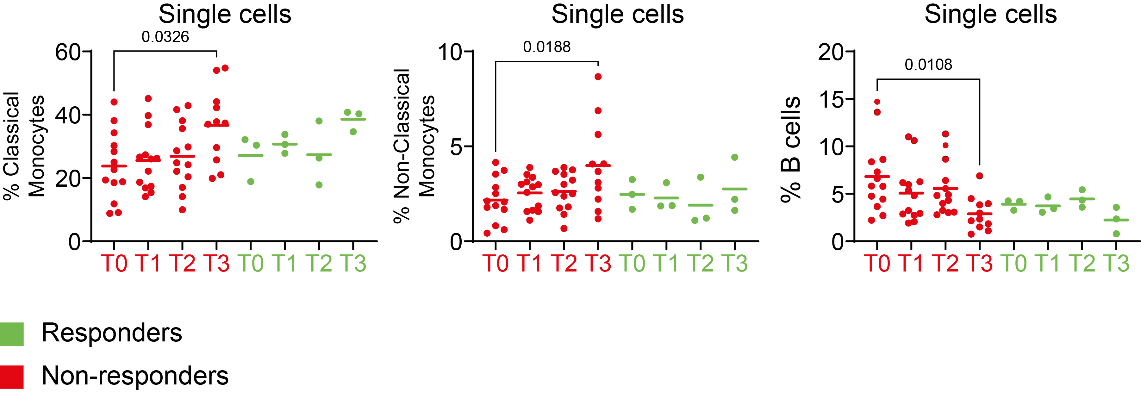


## Fig. S11 Classical monocytes, non-classical monocytes and B cell subset frequencies for all timepoints in responders vs. non-responders.


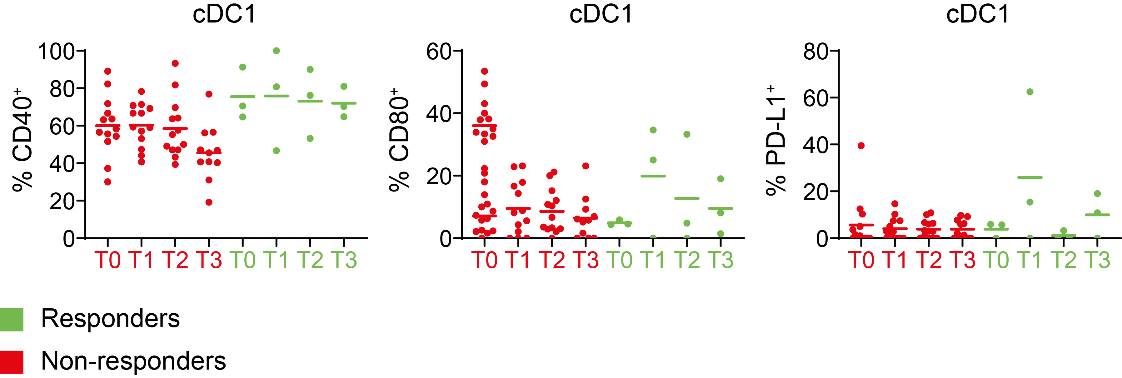


## Fig. S12 CD40, CD80 and PD-L1 expression on cDC1 cells for all timepoints in responders vs. non-responders.


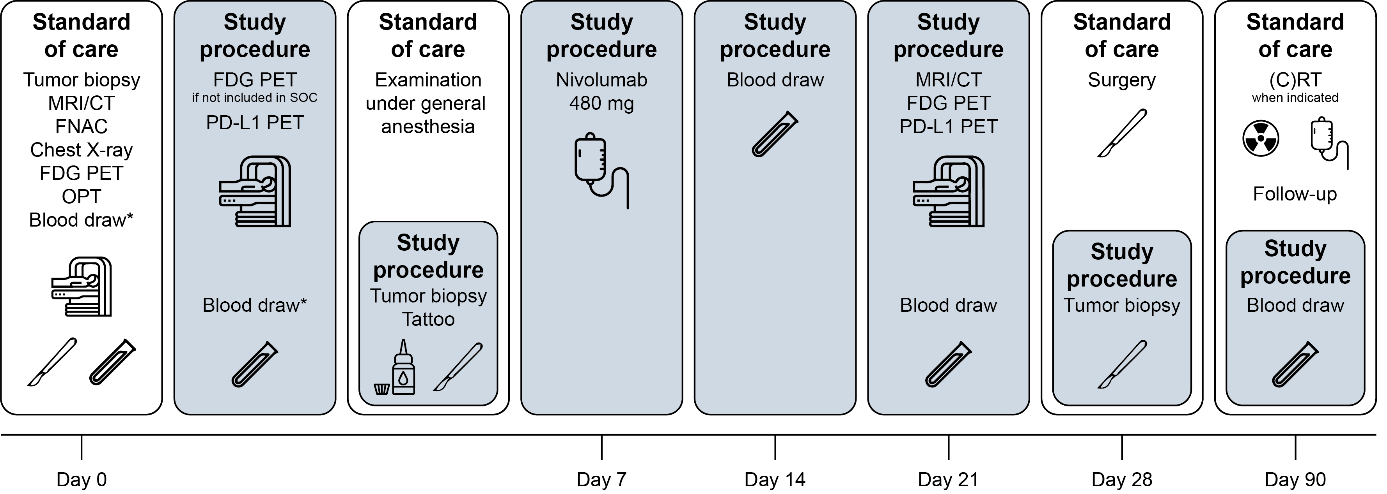


## Fig. S13 Schematic overview of study procedures in addition to standard of care procedures. MRI = magnetic resonance imaging, CT = computed tomography, FNAC = fine needle aspiration cytology, FDG = fluorodeoxyglucose, SOC = standard of care, PET = positron emission tomography, OPT = orthopantomogram, PD-L1 = programmed death receptor ligand-1, (C)RT = (chemo)radiotherapy. *Whenever possible SOC blood withdrawal was combined with baseline study blood withdrawal to minimize patient burden.

## Table S1

|  |  |  |  |  |  |  |  |  |  |  |  |  |  |  |  |  |  |
| --- | --- | --- | --- | --- | --- | --- | --- | --- | --- | --- | --- | --- | --- | --- | --- | --- | --- |
|  | **ID** | **Sex** | **Age** | **Performance Status ECOG** | **Smoking** | **Smoking packyears** | **Alcohol use** | **Tumor site (ICD-10)** | **Laterality** | **Clinical T-stage** | **Clinical N-stage** | **Clinical stage** | **Surgery** | **Neck dissection left** | **Neck dissection right** | **Adjuvant therapy** |  |
|  | 001 | Female | 71 | 1 | Former | 4 | Seldom | C04.1 Lateral floor mouth | Left | T4a | N2b | IVa | Segmental mandibulectomy | I - IV | I - III | CRT |  |
|  | 002 | Male | 69 | 1 | Current | 50 | Former | C04.1 Lateral floor mouth | Right | T4a | N0 | IVa | Segmental mandibulectomy | I - III | I - III | RT |  |
|  | 003 | Male | 65 | 0 | Current | 104 | Former | C03.1 Lower gum | Right | T4a | N0 | IVa | Segmental mandibulectomy | I - III | I - III | RT |  |
|  | 004 | Male | 86 | 0 | Former | 5 | Regular | C03.1 Lower gum | Left | T4a | N0 | IVa | Segmental mandibulectomy | None | I - III | RT |  |
|  | 005 | Female | 73 | 1 | Former | 21 | Regular | C02.1 Border of tongue | Left | T4a | N0 | IVa | Partial glossectomy | I - IV | None | None* |  |
|  | 006 | Male | 68 | 0 | Former | 20 | Regular | C06.0 Cheek mucosa | Left | T3 | N0 | III | Transoral excision | I - IV | None | None |  |
|  | 007 | Female | 76 | 1 | Current | 55 | Regular | C03.0 Upper gum | Right | T4a | N0 | IVa | Segmental mandibulectomy +  partial maxillectomy | I - IV | None | RT |  |
|  | 008 | Male | 73 | 0 | Former | 50 | Regular | C03.1 Lower gum | Left | T4a | N1 | IVa | Segmental mandibulectomy | I - IV | I - III | RT |  |
|  | 009 | Male | 82 | 1 | Never | 0 | Regular | C03.1 Lower gum | Midline | T4a | N0 | IVa | Segmental mandibulectomy | I - III | I - III | † |  |
|  | 010 | Male | 49 | 0 | Current | 30 | Regular | C03.1 Lower gum | Left | T4a | N1 | IVa | Segmental mandibulectomy | I - III | I - III | CRT |  |
|  | 011 | Male | 53 | 1 | Current | 40 | Regular | C04.1 Lateral floor mouth | Left | T3 | N1 | IVa | Marginal mandibulectomy | I - V | I - III | CRT |  |
|  | 012 | Male | 76 | 1 | Never | 0 | Regular | C03.1 Lower gum | Right | T4a | N2b | IVa | Segmental mandibulectomy | None | I - IV | RT |  |
|  | 013 | Female | 59 | 0 | Former | 20 | Regular | C03.1 Lower gum | Left | T4a | N0 | IVa | Segmental mandibulectomy | I - IV | None | RT |  |
|  | 014 | Male | 66 | 0 | Current | 35 | Former | C04.0 Anterior floor of mouth | Left | T3 | N0 | III | Marginal mandibulectomy | I - III | I - III | CRT |  |
|  | 016 | Female | 72 | 1 | Never | 0 | Never | C02.1 Border of tongue | Right | T3 | N0 | III | Partial glossectomy | None | I - IV | RT |  |
|  | 017 | Male | 82 | 1 | Never | 0 | Never | C02.1 Border of tongue | Left | T3 | N3b | IVb | Partial glossectomy | I - IV | None | † |  |
|  |  |  |  |  |  |  |  |  |  |  |  |  |  |  |  |  |  |

Detailed patient characteristics. * Post-operative radiotherapy was indicated but patient opted out. † Patient deceased before start of adjuvant therapy.

## Table S2

|  |  |  |  |  |  |  |  |
| --- | --- | --- | --- | --- | --- | --- | --- |
|  | **ID** | **Timepoint** | **RVT (%)** | **Pathologic response** | **PD-L1 TPS (%)** | **PD-L1 CPS** |  |
|  | 001 | Biopsy | - | - | 38 | 40 |  |
|  |  | Resection | 100 | none | 80 | 90 |  |
|  | 002 | Biopsy | - | - | <1 | <1 |  |
|  |  | Resection | 100 | none | 0 | 10 |  |
|  | 003 | Biopsy | - | - | 5 | 5 |  |
|  |  | Resection | 100 | none | 0 | 0 |  |
|  | 004 | Biopsy | - | - | 70 | 75 |  |
|  |  | Resection | 1 | major | NA | NA |  |
|  | 005 | Biopsy | - | - | 60 | 70 |  |
|  |  | Resection | 1 | major | NA | NA |  |
|  | 006 | Biopsy | - | - | 70 | 80 |  |
|  |  | Resection | 100 | none | 70 | 75 |  |
|  | 007 | Biopsy | - | - | <1 | 0 |  |
|  |  | Resection | 67 | none | 10 | 25 |  |
|  | 008 | Biopsy | - | - | 0 | 0 |  |
|  |  | Resection | 100 | none | 0 | <1 |  |
|  | 009 | Biopsy | - | - | <1 | <1 |  |
|  |  | Resection | 100 | none | 0 | <1 |  |
|  | 010 | Biopsy | - | - | <1 | <1 |  |
|  |  | Resection | 10 | major | <1 | <1 |  |
|  | 011 | Biopsy | - | - | 0 | <1 |  |
|  |  | Resection | 100 | none | 0 | 5 |  |
|  | 012 | Biopsy | - | - | 100 | 100 |  |
|  |  | Resection | 100 | none | 70 | 100 |  |
|  | 013 | Biopsy | - | - | 60 | 70 |  |
|  |  | Resection | 45 | partial | 0 | 0 |  |
|  | 014 | Biopsy | - | - | <1 | <1 |  |
|  |  | Resection | 80 | none | 0 | 0 |  |
|  | 016 | Biopsy | - | - | 80 | 80 |  |
|  |  | Resection | 100 | none | 80 | 90 |  |
|  | 017 | Biopsy | - | - | 15 | 15 |  |
|  |  | Resection | 100 | none | 70 | 80 |  |
|  |  |  |  |  |  |  |  |

Pathologic assessments. Data was collected from baseline tumor biopsy and one representative slide from the tumor resection material (~3 weeks after nivolumab treatment). RVT = residual volume of tumor. TPS = tumor proportion score, CPS = combined positive score.

## Table S3

|  |  |  |  |  |  |  |
| --- | --- | --- | --- | --- | --- | --- |
|  | **Flow cytometry specifications** | **Clone** | **Isotype** | **Catalog no.** | **Manufacturer** |  |
|  | CD1c-AF700 | L161 | Mouse IgG1 | 331530 | Biolegend |  |
|  | CD3-BV421 | SK7 | Mouse IgG1 | 563798 | BD Horizon |  |
|  | CD3-PerCP-Cy5.5 | SK7 | Mouse IgG1 | 332771 | BD Biosciences |  |
|  | CD4-AF700 | RPA-T4 | Mouse IgG1 | 557922 | BD Pharmingen |  |
|  | CD8-V500 | SK1 | Mouse IgG1 | 561618 | BD Horizon |  |
|  | CD11b-PE-Cy7 | ICRF44 | Mouse IgG1 | 557743 | BD Pharmingen |  |
|  | CD11c-APC | SHCL-3 | Mouse IgG2b | 333144 | BD |  |
|  | CD14-PerCP-Cy5.5 | MφP9 | Mouse IgG2b | 562692 | BD Pharmingen |  |
|  | CD16-BV650 | 3G8 | Mouse IgG1 | 563692 | BD Biosciences |  |
|  | CD19-PE-CF594 | HIB19 | Mouse IgG1 | 562294 | BD Horizon |  |
|  | CD25-APC | 2A3 | Mouse IgG1 | 340907 | BD |  |
|  | CD27-BV711 | L128 | Mouse IgG1 | 563167 | BD Horizon |  |
|  | CD33-PE | D3HL60.251 | Mouse IgG1 | A07775 | Beckman Coulter |  |
|  | CD39-BV711 | TU66 | Mouse IgG2b | 563680 | BD |  |
|  | CD40-PE | MAB89 | Mouse IgG1 | PN IM1936U | Beckman Coulter |  |
|  | CD45RA-APC-H7 | HI100 | Mouse IgG2b | 560674 | BD Pharmingen |  |
|  | CD56-BV650 | HCD56 | Mouse IgG1 | 318343 | Biolegend |  |
|  | CD56-FITC | NCAM16.2 | Mouse IgG2b | 345811 | BD |  |
|  | CD80-Pe-Cy7 | L307.4 | Mouse IgG1 | 561135 | BD Pharmingen |  |
|  | CD86-BV650 | 2331 | Mouse IgG1 | 562999 | BD Horizon |  |
|  | CD127-BV421 | HIL-7R-M21 | Mouse IgG1 | 562436 | BD Horizon |  |
|  | BDCA-2-FITC | AC144 | Mouse IgG1 | 130-113-192 | Miltenyi Biotec |  |
|  | BDCA-3-BV421 | M80 | Mouse IgG1 | 344114 | Biolegend |  |
|  | CTLA-4-PE-CF594 | BNI3 | Mouse IgG2a | 562742 | BD |  |
|  | FoxP3-PE | PCH101 | rat IgG2a | 12-4776-42 | eBioscience |  |
|  | HLA-DR-APC | L243 | Mouse IgG2a | 347403 | BD |  |
|  | HLA-DR-BV786 | L243 | Mouse IgG2a | 307642 | Biolegend |  |
|  | Ki-67-FITC | (RUO) | Mouse IgG1 | 556026 | BD Pharmingen |  |
|  | LAG-3-PE-Cy7 | 3DS223H | Mouse IgG1 | 25-2239-42 | eBioscience |  |
|  | PD-1-BV786 | EH12.1 | Mouse IgG1 | 563789 | BD Horizon |  |
|  | PD-1-PE-Cy7 | EH12.1 | Mouse IgG1 | 561272 | BD Pharmingen |  |
|  | PD-L1-BV786 | MIH1 | Mouse IgG1 | 563739 | BD Horizon |  |
|  | PD-L2-BV711 | MIH18 | Mouse IgG1 | 564258 | BD Horizon |  |
|  | Tigit-BV650 | 1G9 | Mouse IgG1 | 744213 | BD OptiBuild |  |
|  | TIM-3-BV421 | F38-2E2 | Mouse IgG1 | 345008 | Biolegend |  |
|  | FVD-eFluor780 | - | - | 65-0865-14 | Thermofisher |  |
|  | TNF-α FITC | MAb11 | Mouse IgG1 | 502906 | Biolegend |  |
|  | IL-5 PE | JES1-39D10 | Rat IgG2a | 554489 | BD Pharmingen |  |
|  | GrB PE-CF594 | GB11 | Mouse BALB/c IgG1 | 562462 | BD Horizon |  |
|  | IFN-y APC | B27 | Mouse IgG1 | 554702 | BD Pharmingen |  |
|  |  |  |  |  |  |  |

List of antibodies used for flow cytometry analyses.
