# Supplementary material for: Circulating T cell status and molecular imaging may predict clinical benefit of neoadjuvant PD-1 blockade in oral cancer
Source: J Immunother Cancer. 2024 Jul 22;12(7):e009278. doi: 10.1136/jitc-2024-009278 (PMC11268040; doi:10.1136/jitc-2024-009278)
Supplement: online supplemental file 2 [file jitc-12-7-s002.docx]

**Safety and tolerability of neoadjuvant nivolumab for locally advanced resectable oral cancer, combined with [18F]BMS-986192 / [18F]-FDG PET imaging and immunomonitoring for response prediction**

| **Protocol ID** | BMS Study ID: CA209-8JD |
| --- | --- |
| **Short title** | NeoNivo |
| **EudraCT number** | 2018-002643-28 |
| **Version** | 9 |
| **Date** | 11-12-2019 |
| **Principal investigators** | **C. Willemien Menke-van der Houven van Oordt, MD PhD**  Department of Medical Oncology, VUmc, Amsterdam  **C. R. Leemans, MD PhD**  Department of Otolaryngology/Head and Neck Surgery, VUmc, Amsterdam |
| **Co-investigators** | *Department of Otolaryngology/Head and Neck Surgery, VUmc, Amsterdam*:  R.H. Brakenhoff, PhD  R. van de Ven, PhD  *Department of Medical Oncology, VUmc, Amsterdam:*  J. Voortman, MD PhD  T.D. de Gruijl, PhD  *Department of Radiology and Nuclear Medicine,* *VUmc, Amsterdam:*  O.S. Hoekstra, MD PhD  R. Boellaard, PhD  B. Windhorst, PhD  *Department of Pathology, VUmc, Amsterdam:*  E. Bloemena, MD PhD  *Department of Oro- and Maxillofacial Surgery, VUMC, Amsterdam:* T. Forouzanfar, MD PhD |
| **Study coordinators** | I.H.C. Miedema, MD  N. E. Wondergem, MD |
| **Independent expert** | N.W.C.J. van de Donk, MD, PhD |
| **Pharmacy** | Pharmacy visiting address:  Klara Bruyn, Anneke Admiraal, Marlies Hutters Apotheek VUmc  De Boelelaan 1117  1081 HV Amsterdam  tel.:020-4443534  fax:020-4444627  e-mail:onderzoeksmedicatie@vumc.nl  Pharmacy delivery address:  Apotheek VUmc, onderzoeksmedicatie  ruimte PK 1 X 070  De Boelelaan 1118  1081 HZ Amsterdam  [tel: 020-4443435](tel:020-4443435)  fax: 020-4444627  email: onderzoeksmedicatie@vumc.nl |
|  |  |

**PROTOCOL SIGNATURE SHEET**

| **Name** | **Signature** | **Date** |
| --- | --- | --- |
| **Head of Departments:**  M. Labots  C.R. Leemans |  |  |
| **Principal Investigators:**  C.W. Menke-van der Houven van Oordt  C.R. Leemans |  |  |
| **Co-investigator:**  R. Boellaard |  |  |

**TABLE OF CONTENTS**

[SUMMARY 11](#_Toc771258)

[1. BACKGROUND AND RATIONALE 13](#_Toc771259)

[1.1 Background 13](#_Toc771260)

[1.1.1 Pharmaceutical and therapeutic background 13](#_Toc771261)

[1.1.2 Oral cancer and immunotherapy 14](#_Toc771262)

[1.1.3 Predictive biomarkers for nivolumab treatment 15](#_Toc771263)

[1.1.4 PET imaging 16](#_Toc771264)

[1.2 Rationale 17](#_Toc771265)

[1.2.1 [18F]BMS-986192 PET 17](#_Toc771266)

[1.2.2 [18F]-FDG PET 17](#_Toc771267)

[1.2.3 Immuno-monitoring 18](#_Toc771268)

[1.2.4 Genomic profiling 19](#_Toc771269)

[1.2.5 Plasma vesicle microRNAs 19](#_Toc771270)

[1.2.6 Rationale for dose selection 20](#_Toc771271)

[1.2.7 Rationale for endpoints 20](#_Toc771272)

[2. OBJECTIVES AND HYPOTHESES 21](#_Toc771273)

[2.1 Primary objectives & hypotheses 21](#_Toc771274)

[2.2 Secondary objectives & hypotheses 21](#_Toc771275)

[3. STUDY DESIGN 22](#_Toc771276)

[3.1 Diagnostic work up 22](#_Toc771277)

[3.2 [^18^F]BMS-986192 and [^18^F]-FDG PET imaging acquisition 22](#_Toc771278)

[3.3 Treatment with nivolumab 23](#_Toc771279)

[3.4 PET guided additional biopsies 23](#_Toc771280)

[3.5 Response evaluation 23](#_Toc771281)

[3.6 Surgery 23](#_Toc771282)

[3.7 Post-surgery follow-up 23](#_Toc771283)

[3.8 Blood measurements: immunomonitoring and cytokine analysis 23](#_Toc771284)

[3.9 Plasma vesicle miRNAs 24](#_Toc771285)

[3.10 Tumor biopsy analysis 24](#_Toc771286)

[3.10.1 Immunomonitoring: IHC 24](#_Toc771287)

[3.10.2 Genomic profiling 24](#_Toc771288)

[4. STUDY POPULATION 26](#_Toc771289)

[4.1 Inclusion criteria 26](#_Toc771290)

[4.2 Exclusion criteria 27](#_Toc771291)

[4.3 Sample size calculation 27](#_Toc771292)

[5. PET IMAGING WITH [18F]BMS-986192 and [18F]-FDG AND NIVOLUMAB TREATMENT 28](#_Toc771293)

[5.1 Radiolabeling of [18F]BMS-986192 28](#_Toc771294)

[5.2 [^18^F]BMS-986192 injection procedure 28](#_Toc771295)

[5.3 [^18^F]BMS-986192/[^18^F]-FDG PET procedure 28](#_Toc771296)

[5.4 Radiation exposure 28](#_Toc771297)

[5.5 Analysis of PET data 29](#_Toc771298)

[5.5.1 [18F]BMS-986192 PET 29](#_Toc771299)

[5.5.2 [^18^F]-FDG PET 29](#_Toc771300)

[5.6 Nivolumab treatment 29](#_Toc771301)

[5.6.1 Nivolumab preparation, handling and dispensing 29](#_Toc771302)

[5.6.2 Dose modifications 30](#_Toc771303)

[5.6.3 Toxicity management algorithms 30](#_Toc771304)

[5.6.4 Discontinuation criteria 30](#_Toc771305)

[5.6.5 Treatment of nivolumab related infusion reactions 30](#_Toc771306)

[6. ENDPOINTS 32](#_Toc771307)

[6.1 Main study parameters/endpoints 32](#_Toc771308)

[6.2 Secondary study parameters/endpoints 32](#_Toc771309)

[7. STUDY PROCEDURES 33](#_Toc771310)

[7.1 Informed Consent 33](#_Toc771311)

[7.2 Inclusion/exclusion criteria 33](#_Toc771312)

[7.3 Medical history 33](#_Toc771313)

[7.4 Prior medications 33](#_Toc771314)

[7.5 Concomitant medications 33](#_Toc771315)

[7.6 Disease details 34](#_Toc771316)

[7.7 Assignment of screening number 34](#_Toc771317)

[7.8 Adverse event monitoring 34](#_Toc771318)

[7.9 (Full) physical exam 34](#_Toc771319)

[7.10 Vital signs 34](#_Toc771320)

[7.11 Laboratory procedures/assessments and blood collection 34](#_Toc771321)

[7.12 Tumor imaging and assessment of disease 35](#_Toc771322)

[7.13 Surgery 35](#_Toc771323)

[7.14 Adjuvant treatment 36](#_Toc771324)

[7.15 Follow-up visits 36](#_Toc771325)

[7.16 Tissue sampling 36](#_Toc771326)

[7.17 Screening period 36](#_Toc771327)

[7.18 Withdrawal/discontinuation 36](#_Toc771328)

[7.18.1 Withdrawal of individual subjects 37](#_Toc771329)

[7.18.2 Replacement of individual subjects after withdrawal 37](#_Toc771330)

[7.18.3 Premature termination of the study 37](#_Toc771331)

[7.19 Schedule of assessments 38](#_Toc771332)

[8. SAFETY REPORTING 40](#_Toc771333)

[8.1 AEs, SAEs and SUSARs 40](#_Toc771334)

[8.1.1 Adverse events (AEs) 40](#_Toc771335)

[8.1.2 Serious adverse events (SAEs) 40](#_Toc771336)

[8.1.3 Suspected Unexpected Serious Adverse Reactions (SUSARs) 41](#_Toc771337)

[8.1.4 Potential Drug Induced Liver Injury (DILI) 41](#_Toc771338)

[8.1.5 Laboratory Test Abnormalities 42](#_Toc771339)

[8.1.6 Complications during surgery 42](#_Toc771340)

[8.2 Serious Adverse Event Collection and Reporting 42](#_Toc771341)

[8.3 Pregnancy 43](#_Toc771342)

[8.4 Overdose 43](#_Toc771343)

[8.5 Other safety considerations 43](#_Toc771344)

[8.6 Annual safety report 44](#_Toc771345)

[8.7 Follow-up of adverse events 44](#_Toc771346)

[9. STATISTICAL ANALYSIS 45](#_Toc771347)

[9.1 Analysis of PET data 45](#_Toc771348)

[9.2.1 Interpatient heterogeneity 45](#_Toc771349)

[9.2.2 Intrapatient heterogeneity 45](#_Toc771350)

[9.2.3 Intratumor heterogeneity 45](#_Toc771351)

[9.3 Correlation between PET data and response 45](#_Toc771352)

[9.4 Correlation between PET data and Blood and Tissue markers 46](#_Toc771353)

[10. ETHICAL CONSIDERATIONS 47](#_Toc771354)

[10.0 Regulation statement 47](#_Toc771355)

[10.1 Recruitment and consent 47](#_Toc771356)

[10.2 Benefits and risks assessment, group relatedness 47](#_Toc771357)

[10.3 Compensation for injury 48](#_Toc771358)

[10.4 Incentives 48](#_Toc771359)

[11. ADMINISTRATIVE ASPECTS, MONITORING AND PUBLICATION 49](#_Toc771360)

[11.1 Handling and storage of data and documents 49](#_Toc771361)

[11.2 Handling and storage of body materials 49](#_Toc771362)

[11.3 Monitoring and Quality Assurance 49](#_Toc771363)

[11.4 Amendments 49](#_Toc771364)

[11.5 Start of study report 49](#_Toc771365)

[11.6 Annual progress report 49](#_Toc771366)

[11.7 End of study report 49](#_Toc771367)

[11.8 Public disclosure and publication policy 50](#_Toc771368)

[REFERENCES 51](#_Toc771369)

[APPENDIX 1: Response Evaluation Criteria in Solid Tumors (RECIST) 1.1 Criteria for evaluating Response in Solid Tumors 55](#_Toc771370)

[APPENDIX 2: Eastern Cooperative Oncology Group (ECOG) Performance Scale. 55](#_Toc771371)

[APPENDIX 3: Management of toxicity due to nivolumab 55](#_Toc771372)

[APPENDIX 4 Women of childbearing potential 63](#_Toc771373)

LIST OF ABBREVIATIONS AND RELEVANT DEFINITIONS

| ABR | ABR form, General Assessment and Registration form, is the application form that is required for submission to the accredited Ethics Committee (In Dutch, ABR = Algemene Beoordeling en Registratie) |
| --- | --- |
| AE | Adverse Event |
| AR | Adverse Reaction |
| ASCO | American Society of Clinical Oncology |
| BMS | Bristol-Myers Squibb |
| CA | Competent Authority |
| CCMO | Central Committee on Research Involving Human Subjects; in Dutch:Centrale Commissie Mensgebonden Onderzoek |
| CI | Confidence Interval |
| CMR | Complete Metabolic Response |
| CR | Complete Response |
| CT | Computer Tomography |
| CTCAE | Common Terminology Criteria for Adverse Events |
| CTLA-4 | Cytotoxic T-Lymphocyte–associated Antigen 4 |
| CV | Curriculum Vitae |
| DC | Dendritic Cell |
| DILI | Drug Induced Liver Injury |
| DNA | Deoxyribonucleic Acid |
| DSMB | Data Safety Monitoring Board |
| EANM | European Association of Nuclear Medicine |
| ECOG | Eastern Cooperative Oncology Group |
| EMA | European Medicines Agency |
| ESMO | European Society for Medical Oncology |
| EU | European Union |
| EudraCT | European drug regulatory affairs Clinical Trials |
| FDG | Fludeoxyglucose (^18^F) |
| FFPE | Formalin-Fixed Paraffin-Embedded |
| GCP | Good Clinical Practice |
| GMP | Good Manufacturing Practice |
| h | hour |
| HLA | Human Leukocyte Antigens |
| HPV | Human Papilloma Virus |
| HR | Hazard Ratio |
| IB | Investigator Brochure |
| IC | Informed Consent |
| IHC | Immunohistochemistry |
| IMP | Investigational Medicinal Product |
| IMPD | Investigational Medicinal Product Dossier |
| ITIM | Immunoreceptor Tyrosine-based Inhibition Motif |
| ITSM | Immunoreceptor Tyrosine-based Switch Motif |
| IV | Intravenous |
| PET | Positron Emission Tomography |
| mAb | Monoclonal Antibody |
| METC | Medical research ethics committee (MREC); in Dutch: medisch ethische toetsing commissie (METC) |
| miRNA | Micro Ribonucleic Acid |
| min | minutes |
| MRI | Magnetic Resonance Imaging |
| NCI | National Cancer Institute |
| NSCLC | Non Small Cell Lung Cancer |
| OS | Overall Survival |
| PD | Progressive Disease |
| PD-1 | Programmed Cell Death 1 |
| PD-L1 | Programmed Cell Death Ligand 1 |
| PD-L2 | Programmed Cell Death Ligand 2 |
| PERCIST | Positron Emission Tomography Response Criteria in Solid Tumors |
| PR | Partial Response |
| PMR | Partial Metabolic Response |
| RECIST | Response Evaluation Criteria In Solid Tumors |
| r/m HNSCC | Recurrent/metastasized Head and Neck Squamous Cell Carcinoma |
| SAE | Serious Adverse Event |
| SD | Stable Disease |
| SNP | Single Nucleotide Polymorphism |
| SPC | Summary of Product Characteristics (in Dutch: officiële productinfomatie IB1-tekst) |
| Sponsor | The sponsor is the party that commissions the organisation or performance of the research, for example a pharmaceutical company, academic hospital, scientific organisation or investigator. A party that provides funding for a study but does not commission it is not regarded as the sponsor, but referred to as a subsidising party. |
| SMR | Stable Metabolic Disease |
| SUSAR | Suspected Unexpected Serious Adverse Reaction |
| SUV | Standardized Uptake Value |
| TDLN | Tumor Draining Lymph Node |
| Th | T helper |
| TILs | Tumor Infiltrating Lymphocytes |
| Treg | Regulatory T-cell |
| ULN | Upper Limit of Normal |
| VOI | Volume of Interest |
| V-type | Variable Type |
| Wbp | Personal Data Protection Act (in Dutch: Wet Bescherming Persoonsgevens) |
| WMO | Medical Research Involving Human Subjects Act (in Dutch: Wet Medisch-wetenschappelijk Onderzoek met Mensen |
| WOCBP | Women of Childbearing Potential |

# SUMMARY

Rationale: Intensive treatment regimens with surgical resection and adjuvant (chemo)radiotherapy of patients with locally advanced oral cancer still result in only 50-60% cure rate, leaving a substantial group of patients who will develop a local recurrence or distant metastases with minimal curative salvage treatment options. Treatment with anti-PD-1 monoclonal antibodies (mAbs) has shown promise in patients with recurrent/metastatic head and neck squamous cell carcinoma (r/m HNSCC). This supports the hypothesis that including treatment with anti PD-1 mAb nivolumab could improve the outcome for patients with locally advanced oral cancer resulting in a higher cure rate. Response rate with nivolumab was below 20% in unselected patients with r/m HNSCC. Therefore, biomarkers for response are urgently needed. Tumor PD-L1 immunohistochemistry (IHC) was shown to be related to nivolumab response but cannot be reliably used for patient selection. Temporal and spatial heterogeneity of tumor PD-L1 expression (within and between tumor lesions) might be responsible for its suboptimal predictive value as biomarker of response. Therefore there is a need to further evaluate tumor PD-L1 expression as predictive biomarker, as well as exploring alternatives. Serial PET imaging with [^18^F]BMS-986192 (anti-PD-L1 tracer) and [^18^F]-FDG has the potential to provide whole body information of the patient over time, at baseline as well as on treatment and represents a biomarker for toxicity and efficacy. In addition, we will investigate the immunophenotype of the patient and tumor, as well as the presence of neoantigens and other potential other biomarkers such as plasma vesicle miRNAs.

Objectives: 1) To assess uptake of [^18^F]BMS-986192 in tumor lesions before and after treatment with nivolumab, in relation to [^18^F]-FDG uptake as potential whole body biomarker for response. 2) To evaluate safety and tolerability of neoadjuvant nivolumab 3) To evaluate tumor and blood immunoprofiling, presence of neoantigens, and other potential biomarkers of response such as plasma vesicle miRNAs.

Study population: Patients with locally advanced oral cancer amenable for curative treatment including surgery.

Intervention: After screening, eligible subjects will undergo a standard [^18^F]-FDG PET scan and an experimental [^18^F]BMS-986192 PET scan. Patients will be treated with a single dose of 480 mg of nivolumab followed by an experimental [^18^F]BMS-986192 and [^18^F]-FDG PET scan 21 days later to monitor changes in biodistribution and early therapeutic effects. Patients are planned to undergo surgery within 30 days after diagnosis. Tissue samples will be collected at diagnostic panendoscopy and at surgery. Blood samples will be collected at multiple time points. Up to two additional biopsies can be performed depending on the baseline imaging results and whether the location can be reached safely. Treatment and follow-up (12 months) after surgery will be according to standard procedures.

Main study parameters/endpoints: Visual and quantitative [^18^F]BMS-986192 and [^18^F]-FDG uptake measurements in tumor before and after nivolumab treatment. Adverse events of neoadjuvant nivolumab. Correlation of imaging results with blood and tissue immune parameters, presence of neoantigens, plasma vesicle miRNA’s, as well as (pathological) response.

Nature and extent of the burden and risks associated with participation, benefit and group relatedness: Nivolumab is an active drug for the treatment of r/m HNSCC. It is therefore likely that patients derive benefit from this study. Toxicity has been demonstrated to be manageable and the safety profile acceptable. No toxicity is expected from the PET scans itself and the total amount of radiation exposure is moderate. Immediate effects are not anticipated. Up to two additional biopsies are allowed in this study. Although this is demanding for patients, biopsies in cancer patients are considered safe with a low and acceptable complication rate. No side effects are expected from blood withdrawal.

|  |  |  |  |  |  |  |  |  |  |  |  |  |  |  |  |  |  |  |  |  |  |
| --- | --- | --- | --- | --- | --- | --- | --- | --- | --- | --- | --- | --- | --- | --- | --- | --- | --- | --- | --- | --- | --- |

# BACKGROUND AND RATIONALE

## Background

### Pharmaceutical and therapeutic background

The importance of intact immune surveillance in controlling outgrowth of cancer has been surmised for decades [1]. Mounting evidence shows a correlation between tumor-infiltrating lymphocytes (TILs) in cancer tissue and favourable prognosis in various malignancies [2-6]. In particular, the presence of CD8+ T-cells and the ratio of CD8+ effector T-cells / FoxP3+ regulatory T-cells seems to correlate with improved prognosis and long-term survival in many solid tumors.

The PD-1 receptor-ligand interaction is a major pathway hijacked by tumors to suppress immune control. The normal function of PD-1, expressed on the cell surface of activated T-cells under steady state conditions, is to down-modulate unwanted or excessive immune responses, including autoimmune reactions. PD-1 (encoded by the gene *PDCD1*) is an Ig superfamily member which has been shown to negatively regulate antigen receptor signalling upon engagement of its ligands (PD-L1 and/or PD‑L2) [7, 8]. PD-1 and family members are type I transmembrane glycoproteins containing an Ig Variable-type (V-type) domain responsible for ligand binding and a cytoplasmic tail which is responsible for the binding of signalling molecules. The cytoplasmic tail of PD-1 contains 2 tyrosine-based signalling motifs, an immunoreceptor tyrosine-based inhibition motif (ITIM) and an immunoreceptor tyrosine-based switch motif (ITSM). Following T-cell stimulation, PD‑1 recruits the tyrosine phosphatases SHP-1 and SHP-2 to the ITSM motif within its cytoplasmic tail, leading to the dephosphorylation of effector molecules such as CD3ζ, PKCθ and ZAP70 which are involved in the CD3 T-cell signalling cascade [7, 9-11]. The mechanism by which PD-1 down modulates T-cell responses is similar to, but distinct from that of CTLA-4 as both molecules regulate an overlapping set of signalling proteins [12, 13]. PD-1 was shown to be expressed on activated lymphocytes including peripheral CD4+ and CD8+ T-cells, B-cells, regulatory T-cells (Tregs) and Natural Killer cells [14, 15]. Expression has also been shown during thymic development on CD4-CD8- (double negative) T-cells as well as subsets of macrophages and dendritic cells [16]. The ligands for PD-1 (PD-L1 and PD-L2) are constitutively expressed or can be induced in a variety of cell types, including non-hematopoietic tissues as well as in various tumors [12, 17-19]. Both ligands are type I transmembrane receptors containing both IgV- and IgC-like domains in the extracellular region and contain short cytoplasmic regions with no known signalling motifs. Binding of either PD-1 ligand to PD-1 inhibits T-cell activation triggered through the T-cell receptor. PD-L1 is expressed at low levels on various non-hematopoietic tissues, most notably on vascular endothelium, whereas PD-L2 protein is mostly expressed on antigen-presenting cells found in lymphoid tissue or chronic inflammatory environments. PD-L2 was recently described to be present within the tumor microenvironment of multiple tumor types, with about 60% of HNSCC expressing PD-L2 at some level by IHC [20]. Expression was detectable on stromal cells, immune infiltrate, epithelial cells as well as tumor cells. Initially PD-L2 was thought to control immune T-cell activation in lymphoid organs, whereas PD-L1 would serve to dampen unwarranted T-cell function in peripheral tissues [12]. Although healthy organs express little (if any) PD-L1, a variety of cancers were demonstrated to express abundant levels of this T-cell inhibitor, with about 80% of HNSCC expressing PD-L1 at some level by IHC [21]. PD-1 has been suggested to regulate tumor-specific T-cell expansion in subjects with melanoma [22]. For HNSSC, the PD-1/PD-L2 interaction might be clinically relevant, since it was recently described that in 18 of 21 HNSCC tested by IHC staining, PD-L2 was actually expressed more abundantly than PD-L1 [20]. This suggests that the PD-1/PD-L1/PDL2 pathway plays a critical role in tumor immune evasion for head and neck cancer patients and should be considered as an attractive target for therapeutic intervention.

Nivolumab (also referred to as BMS-936558 or MDX1106) is a fully human monoclonal antibody (HuMAb; immunoglobulin G4 [IgG4]-S228P) that targets the programmed death-1 (PD-1) cluster of differentiation 279 (CD279) cell surface membrane receptor. PD-1 is a negative regulatory molecule expressed by activated T and B lymphocytes [23]. Binding of PD-1 to its ligands, programmed death–ligands 1 (PD-L1) and 2 (PD-L2), results in the down-regulation of lymphocyte activation. Inhibition of the interaction between PD-1 and its ligands promotes immune responses and antigen-specific T-cell responses to both foreign antigens as well as self-antigens.

### Oral cancer and immunotherapy

Current trials in r/m HNSCC show that 10-20% of patients have durable responses following treatment with checkpoint inhibitors targeting PD-1 [24-26]. These are paradigm shifting results in head and neck oncology that have resulted in EMA approval of the PD-1 inhibitor nivolumab in patients who experience progression of disease after treatment with platinum-based treatment. Nonetheless, many urgent questions remain unsolved. Importantly, not all patients respond to nivolumab, so what characterizes the patients who do respond to anti-PD1 immunotherapy? Are these patient characteristics, tumor characteristics or immunesystem responses? PD-L1 expression has been studied as an obvious candidate and although PD-L1 expression in tumor biopsies is correlated with an improved response rate in head and neck cancer as well as other tumor types [27], treatment selection based on PD-L1 expression in biopsies is not conclusive as responses to therapy are also observed in PD-L1 negative tumors. Hence, more reliable biomarkers are needed to predict response and to improve efficacy of immunotherapy approaches.

In addition, there is also less experience with immunotherapy in the neoadjuvant setting, particularly in head and neck cancer. Many head and neck cancer patients are treated by upfront surgery, and (neo)adjuvant treatment for advance stage patients may well improve outcome. Many trials with PD-1 inhibitors are currently focusing on combinations with curative radiotherapy or chemoradiation in the locally advanced setting, but neoadjuvant treatment might be effective as well. Combinations in the perioperative setting are much less studied, while surgical resection is standard of care for patients with locally advanced oral cancer. Surgical treatment of advanced stage oral cancer often includes reconstruction using a free flap, as well as adjuvant radiotherapy or chemoradiation when high-risk features are present [28]. Even with these intensive treatment regimens, 40-50% of these patients will develop a local recurrence or distant metastases with minimal curative salvage treatment options. Neoadjuvant treatment has the advantage of potentially reducing tumor volume and eliminating micrometastases, thus potentially increasing the rate of long term survival. Promising data from small studies with neoadjuvant PD-1 inhibitor nivolumab in resectable non-small lung cancer (NSCLC) and pembrolizumab in human papillomavirus (HPV)-negative head and neck cancer have been presented (data on NSCLC have just been published) at the ESMO and ASCO meetings, respectively, showing 25-40% response rate after 1-2 cycles of treatment [29, 30]. On the most recent ESMO meeting similar results were reported for neoadjuvant nivolumab in a small group of resectable head and neck cancer (HPV positive and negative) with tumor shrinkage observed in 40-50% of patients [31]. Hence, promising initial results, but again only subgroups of patients seem to respond and critical information to better select patients who might benefit and to improve treatment in those who will not benefit with anti-PD-1 alone, is missing.

We hypothesize that neoadjuvant treatment with PD-1 inhibitor nivolumab will lead to antitumor immune activation and improved clinical outcome in advanced stage oral cancer patients scheduled for curative surgery. To prove these hypotheses a randomized controlled trial (RCT) should be designed, but the combination of neoadjuvant immunotherapy, extensive surgery and (when indicated) postoperative combination treatment with chemoradiation may lead to potentially unforeseen complications. Therefore, this study was designed to evaluate safety and tolerability of neoadjuvant nivolumab and investigate potential biomarkers for response that could be evaluated in a larger RCT. Based on the studies and efficacy of nivolumab in the recurrent/ metastatic setting, we anticipate in a study of 15 patients at least 3-4 patients (~25%) with a (histopathological) response in the resection material, likely coinciding with immune activation in the tumor.

Please see the nivolumab Investigator Brochure [32] for the full preclinical and clinical data.

### Predictive biomarkers for nivolumab treatment

Selecting those patients that benefit the most from nivolumab treatment is challenging. Although other biomarkers may also be of value, companion biomarker development has focused on the level of PD-L1 expression by IHC [33]. Among 361 HNSCC patients with recurrent/metastatic platinum resistant disease treated in the phase III trial which randomized nivolumab vs investigator’s choice of therapy, the recently updated findings with a minimum of 2 years of follow-up show a median overall survival with nivolumab of 7.7 months (95% confidence interval [CI], 5.7 to 8.8) versus 5.1 months (95% CI, 4.0 to 6.2) compared to the patients receiving standard therapy. The overall survival gain was limited although significantly longer with nivolumab than with standard therapy (hazard ratio for death, 0.68; 95 CI, 0.54 to 0.86). The response rate was 13.3% in the nivolumab group versus 5.8% in the standard-therapy group and 2 year OS rate was 16.6% with nivolumab (95% CI 12.4-22.0) vs. 6% in the control arm (95% CI 2.7-11.3) [25, 34]. Although it was only a modest benefit when considering the numbers, the results are a paradigm shift for the individual patients with a long lasting response as the treatment options for recurrent/metastatic head and neck cancer were very limited up to then.

A total of 72% of the patients who underwent randomization (225 of 272 patients) had quantifiable PD-L1 expression, of which 57.3% had ≥ 1% PD-L1 expression. PD-L1 IHC appeared to have limited value as biomarker predicting improved OS in the phase III HNSCC study. Across the pre-specified expression levels (1%, 5%, and 10%), in patients with PD-L1 expression ≥ 1 % there was a 45% reduction of risk of death (HR 0.55 (95% CI 0.36-0.83)) compared to a 27% reduction in risk of death for patients with expression of < 1%, the interaction was however not significant [25]. As the overall survival was similar in patients treated with nivolumab who were PD-L1 positive or negative, this did not justify IHC-based PD-L1 expression as a selection biomarker for nivolumab therapy.

In comparison, the recently published phase II paper with 171 patients with r/m HNSCC, resistant to platinum and cetuximab, who were treated with pembrolizumab, another PD-1 directed antibody, showed comparable results. Overall response rate was 16% (95% CI, 11% to 23%, with a median duration of response of 8 months, range, 2+ to 12+ months). Median progression-free survival was 2.1 months, and median overall survival was 8 months. 82% of the patients were PD-L1 positive and response rates were similar in all PD-L1 subgroups [26].

In conclusion, immunotherapy in head and neck cancer showed for the first time durable responses in patients with untill now incurable disease. However, efficacy was noted only in subgroups leading to questions on patient selection and improvement of therapy. Tumor PD-L1 expression seems to be related to response but the signal is not straightforward since responses are seen across the spectrum of PD-L1 expression and even in patients that are PD-L1 IHC negative. Importantly, companion diagnostics for PD-L1 IHC expression measurement greatly differ between the various drug companies, which complicates interpretation of results [35]. Adding to this complexity, the level of tumor PD-L1 expression is variable over time and influenced by several host and environmental factors such as TNM stage, chemotherapy and cytokines like IFN-α. PD-L1-negative tumors as defined by IHC can become positive and vice versa [36]. Temporal and spatial variation of tumor PD-L1 expression (within and between tumor lesions) might be responsible for its suboptimal predictive value for treatment benefit. Even more so, for head and neck cancer Yearly et al. showed that expression of PD-L2 on tumor, stroma and infiltrate combined, independently of PD-L1 expression correlated with response to anti-PD-1 therapy with the anti-PD-1 agent pembrolizumab [20], suggesting that promising biomarkers may be identified. Nevertheless, and despite these conflicting results, some immunotherapy trials do already require positive tumor PD-L1 staining for inclusion. In view of the above there is an urgent need to define an alternative and more optimal biomarker for nivolumab therapy response.

### PET imaging

PET imaging using biological active tracers could represent a valuable method to identify suitable biomarkers for personalized (immuno)therapy. The advantage of PET over IHC is the possibility to visualize tracer distribution in the entire body to appreciate heterogeneity, and to repeat imaging during the course of treatment/disease to investigate changes over time [37].

Recently, PD-1 and PD-L1 expression has been imaged at VUmc in patients with NSCLC with ^89^Zr labeled nivolumab (PD-1) and [^18^F]BMS-986192 (PD-L1 ) PET, respectively [38]. In all 7 patients tumor uptake was visualized for both tracers in all patients. For lesions with < 50% PD-L1 IHC, [^18^F]BMS-986192 PET showed moderate uptake (SUV 3.4 (±2.9)) compared to lesions with ≥50% PD-L1 IHC (SUV 7.1 (±6.0); p = 0.22). These data demonstrated the feasibility to show inter- and intra-patient heterogeneity with [^18^F]BMS-986192, supporting its further development as a potential biomarker for response to nivolumab.

## Rationale

Immunotherapy with checkpoint inhibitors is moving to the (neo)adjuvant setting, after having shown paradigm changing results in the recurrent/metastatic setting. Advanced stage oral cancers are primarily treated by a combination of resection and adjuvant postoperative radiotherapy or chemoradiation. Despite these invasive treatment protocols that may cause toxicity and have impact on quality of life, 5-year overall survival rates remain disappointing at 50-60%. Neoadjuvant immunotherapy could facilitate resection and induce elimination of micrometastatic disease, but experience is limited. Safety and tolerability are still an issue before phase III trials can be designed. Also choices should be made for additional translational biomarker research. Feasibility trials as the current one provide the safety and tolerability data and the additional opportunity to investigate potential biomarkers that might be interesting to integrate in subsequent studies such as molecular imaging, information on immune status and neoantigen expression.

### [18F]BMS-986192 PET

Selecting those patients that benefit the most from nivolumab treatment is challenging. As discussed above, until now companion biomarker development has focused on the level of PD-L1 expression by IHC. [18F]BMS-986192 PET in NSCLC has shown non-invasive quantification of the PD-L1 receptor in tumors [38], so far the most frequently used (ex-vivo) tissue biomarker for patient selection in current trials with anti-PD-(L)1 mAbs. With [18F]BMS-986192 PET it will be possible to visualize and quantify in vivo PD-L1 IHC and provide unique comparative insight in the relative performance of tumor tissue PD-L1 IHC. A tumor biopsy providing tissue for PD-L1 IHC shows the level of expression for a small part of one tumor lesion. Whether this is representative for other parts of that lesion or for other tumor lesions is not known and might influence the predictive value of PD-L1 IHC. Because the PET technique is non-invasive and whole body, it allows to quantify whole tumor uptake as well as looking at heterogeneity within and between tumor lesions, changes over time during treatment and uptake at immunomodulatory locations such as tumor-draining lymph nodes (TDLN).

We hypothesize that [^18^F]BMS-986192 tumor uptake will differ between tumor lesions (primary tumor and affected lymph nodes) and patients and that the uptake is correlated with PD-L1 expression and response to nivolumab treatment. In addition, we hypothesize that expression level of PD-L1 will change during treatment with nivolumab. Quantitative [^18^F]BMS-986192 PET might elucidate spatial and temporal heterogeneity and changes in expression in patients with locally advanced oral cancer before and on treatment with nivolumab.

### [18F]-FDG PET

As the treatment of HNSCC patients often involves multimodality treatment with surgery, radiotherapy and concomitant systemic therapy which causes extensive morbidity, early response evaluation could significantly improve quality of life and potentially survival as treatment can be adapted accordingly. A study with induction chemotherapy indeed showed promising results for predicting response in patients with significant reduction in the [18F]-FDG uptake in all lesions expressed as total lesion glycolysis (responders -85.9% ± 18.6 vs non-responders 36.4±64.1) [39]. In a meta-analysis the same conclusion was reached, early [^18^F]-FDG shows promise for response predication, however, standardized evaluation is required for further conclusions [40].

Regarding the usefulness of early [^18^F]-FDG imaging for patients treated with immunotherapy there is only limited evidence in literature so far. For patients with melanoma treated with a variety of immunotherapies including nivolumab [^18^F]-FDG changes showed mainly increase in [^18^F]-FDG uptake at 3 weeks after start of treatment in patients who responded at the later evaluation time points. These findings are consistent with immune activation in the tumor leading to increased glucose consumption [41].

Based on these results, early increase in [^18^F]-FDG uptake in patients with locally advanced oral cancer treated neoadjuvantly with nivolumab are expected to predict histopathological response in the resection material.

### Immuno-monitoring

It is known that the immune system can protect against the development of cancer. This follows the normal immune response involving uptake of tumor antigens by dendritic cells (DC), presentation of these antigens in TDLN to naive CD4+ and CD8+ T cells, which will subsequently result in T cell priming, skewing and activation, expansion of CD8+ cytotoxic T cells and CD4+ T helper (Th) cells and the induction of a tumor specific response. Unfortunately the immune system is not always capable of killing all the tumor cells, or the tumor cells have successfully adapted such that the immune system does not recognize them anymore, leading to tumor escape, outgrowth and metastases.

Multiple mechanisms can be involved in this immune escape and several of these have been described to occur in HNSCC development, such as downregulation of HLA molecules preventing T cell recognition, secretion of tumor-derived immune suppressive factors (cytokines like TGF-beta or IL-10), inability of T-cells to infiltrate the tumor, attraction of immature, suppressive myeloid cells from the bone-marrow, loss of expression of co-stimulatory molecules and increased expression of inhibitory receptors and ligands or suppression of immune response by promoting the development and expansion of Tregs [42-44].

The composition of the tumor immune infiltrate can affect and may predict response to therapy, both to conventional therapies like chemoradiation and to immunotherapy with checkpoint inhibitors. It can also provide leads as to why certain patients do and others do not respond to agents targeting the PD-1/PD-L1 pathway [45]. What type of immune infiltrate is present in HNSCC can also be determined by the site of the tumor (oral cavity, oropharynx, larynx) and has not been studied in great detail yet. Similarly to other tumor types, enhanced CD8+ T cell infiltration in the tumor is associated with a better prognosis in HNSCC [46]. Interestingly, unlike in most other cancer types, there have been reports that increased Treg frequencies correlate with a good prognosis in HNSCC (reviewed in Fridman et al 2012 [47]). This could reflect a general high immune infiltration, including CD8 T cells, suggestive of an active anti-tumor immune reaction at play with collateral Treg infiltration. Tsujikawa et al. recently published an IHC-based study in a small group of 38 HNSCC patients, showing that a T cell inflamed immune signature, as well as a hypo-inflamed immune signature (little to no T cell or myeloid cell activation) were associated with good prognoses, while tumors with a myeloid inflamed profile displayed poor survival (independent of HPV status) [48]. In addition, there are several indications that head and neck cancers impact the general immune system. For example the absence of specific immune cell subsets is associated with clinical outcome [49]. In addition, the white blood cells in head and neck cancer patients have undergone markedly molecular changes [50].

We will perform extensive immune monitoring to assess baseline immune status as well as changes in immune cell infiltrate in tumor specimen prior to and after neo-adjuvant nivolumab using flow cytometry on single cell suspensions as well as six-parameter IHC staining on FFPE material. Similar analyses will be performed on surgically removed lymph nodes, as well as additionally obtained biopsies in case of heterogeneous result on baseline PET imaging). Additionally we will monitor changes in systemic immune responses by profiling peripheral blood at several time points during the treatment (as indicated), analysing frequencies and activation status of T cell subsets, peripheral blood dendritic cells and myeloid derived suppressor cells. We anticipate that we will find either pre-treatment immune traits or alterations in immune activation and immune infiltrate composition upon neoadjuvant nivolumab therapy.

### Genomic profiling

HNSCC is caused by smoking and excessive alcohol consumption. However, last decade it has been shown that also HPV infections cause HNSCC, particularly in the oropharynx. HPV-positive and HPV-negative tumors develop at the molecular level and are considered distinguished disease entities. In the oral cavity tumors are generally HPV-negative, and we recently showed that the HPV attributable fraction is below 3%. In fact it can be neglected in the current study, but will be determined during sequence analysis just in case.

HPV-negative HNSCC are characterized by many genetic changes including copy number alterations and mutations [28, 51, 52]. Also splice variants do occur and expression of embryonic genes that might all function as neoantigens. Standard approach at present is whole exome sequencing combined with RNAseq to identify potential neoantigens [53].

The sequencing data can also be used to determine the copy number profiles and HPV status using the off-target reads, but it is safer to perform parallel low coverage whole genome run as this is a cheap, more reliable and well validated analysis method. This is of relevance as there is a subgroup of particularly oral cancers that is characterized by few genetic changes also indicated as ‘copy-number-silent’. How this molecular subgroup relates to immune status and response to anti-checkpoint therapy, remains a major question.

Mutations and RNAseq data will be mined for neoantigens, the copy number profiles, and the data associated with immuneprofiles in the tumor, the peripheral blood and response in the tumor when occurring. With the planned number of patients, strong associations cannot be identified but the added value of these approaches in a larger RCT can be studied.

### Plasma vesicle microRNAs

Most, if not all, tumors shed nucleic acids into the circulation, including fragments of DNA and miRNAs from living and dying cells. Apart from the mutational landscape, disease specific dysregulation of miRNA expression is one of the hallmarks of HNSCC [28, 54, 55]. Due to the abundance of RNA species from numerous sources in the circulation, tumor derived RNA species are difficult to detect and monitor in whole blood samples. Plasma vesicle miRNAs have been shown suitable for disease monitoring in cancer patients [56]. HNSCC-specific plasma vesicle miRNA profiles are hypothesized to be useful for therapy response monitoring and potentially for treatment outcome prediction in patients with HNSCC. Possible associations between miRNA findings and immune parameters and changes therein will also be investigated in an integrated analysis.

### Rationale for dose selection

Patients will be treated with a single flat dose of 480mg nivolumab IV which has been recently approved as a 4-weekly dose [57]. This single dose allows for maximal treatment before surgery, as the timelines from diagnosis to surgery are strictly defined in the current guidelines.

### Rationale for endpoints

This is a feasibility study of neoadjuvant nivolumab in locally advanced oral cancer combined with [^18^F]BMS-986192 and [^18^F]-FDG PET as well as immuno-monitoring and DNA profiling on tissue and blood in order to identify biomarkers for response and investigate safety.

The primary endpoint of this study is [^18^F]BMS-986192 uptake in tumor lesions and safety and tolerability of neoadjuvant nivolumab. [^18^F]BMS-986192 tumor uptake will be quantified, tumor uptake heterogeneity between patients and within and between tumor lesions of the same patient will be assessed. These results will be correlated with [^18^F]-FDG uptake, as effective treatment with immune therapy may lead to increase in early [^18^F]-FDG uptake, as well as to (pathological) response.

The secondary endpoints include the correlation of the imaging and response data to measured blood and tissue parameters. The immune status will be assessed in the tumor by multiplex immunostaining (see section 3.10.1) and in peripheral blood and TDLN by flowcytometry for the frequency and activation status of immune subsets (see section 3.8). Cytokine profiling may be performed in plasma or from TDLN in ex vivo O/N cultures.

In addition, the presence of neoantigens and the molecular background of the tumor as copy-number-high or copy-number-silent will be determined. Neoantigen analysis will be performed by DNA sequencing using a combined approach of low coverage whole genome sequencing for copy numbers, and exome enrichment sequencing for mutations. Normal DNA from blood will be analyzed in parallel to filter SNPs. In addition RNA sequencing will be performed and the data mined as previously described [53].

# OBJECTIVES AND HYPOTHESES

## Primary objectives & hypotheses

1. To investigate heterogeneity in tumor uptake of [^18^F]BMS-986192 between patients and within tumor lesions of the same patient (primary tumor and TDLN/lymph node metastases) before treatment, in relation to changes in [^18^F]-FDG uptake before and on treatment

*Hypothesis: interpatient and intrapatient heterogeneity in tumor accumulation of [^18^F]BMS-986192 is expected and can be visualized. [^18^F]-FDG allows identification of viable tumor.*

2. To investigate the feasibility and safety of neoadjuvant nivolumab immunotherapy prior to surgery for locally advanced oral cancer.

*Hypothesis: neoadjuvant immunotherapy with nivolumab is feasible and safe, and will lead to immune activation and clinical response in a subset of patients.*

## Secondary objectives & hypotheses

3. To investigate effects of nivolumab treatment on PD-L1 expression and availability for tracer binding in the patient and the relation to (changes in) [^18^F]-FDG uptake.

*Hypothesis: changes in tumor accumulation of [^18^F]BMS-986192* *during treatment will occur and can be visualized.*

4. To investigate the relationship between [^18^F]BMS-986192 tumor uptake and tumor cell- and tumor infiltrating lymphocyte (TIL) PD-1 and PD-L1 expression as well as other immune parameters.

*Hypothesis: changes in tumor accumulation of [^18^F]BMS-986192* *during treatment will occur and can be visualized.*

5. To investigate changes in [^18^F]-FDG uptake during treatment.

*Hypothesis: Early changes on [^18^F]-FDG imaging on treatment will allow early response prediction.*

6. To investigate the genomic profile of the tumor (neoantigens, mutational load, copy number changes and splice variants), in relation to [^18^F]BMS-986192 uptake, immune activation parameters and clinical response.

*Hypothesis: immunotherapy with checkpoint inhibitors is most effective in tumors with high mutational load and head and neck cancers carry a large number of copy number changes, mutations and splice variants. We expect that when quantified, this relates to immune activation assuming presence of PD-L1 expression/uptake.*

7. To investigate blood based analyses of the immuneprofile and plasma vesicle miRNAs on treatment and after treatment, in relation to immune activation parameters and clinical outcome.

*Hypotheses: changes in the immune profile are a potential biomarker for immune activation in the tumor as detected in the surgical resection material. Changes in the plasma vesicle miRNA profile are a potential predictive and prognostic biomarker for clinical outcome.*

# STUDY DESIGN

In this pilot study we will study potential biomarkers and safety of nivolumab in a neoadjuvant setting combined with the routine treatment protocol applied for locally advanced oral cancer patients.

The routine diagnostic workup with MRI and [^18^F]-FDG imaging, and clinical examination using panendoscopy followed by surgical excision allows for 1) collection of tumor and TDLN/pathological lymph node specimens as well as blood samples before and after induction treatment with nivolumab and 2) pretreatment and on-treatment imaging with [^18^F]BMS-986192 and [^18^F]-FDG, respectively. Whole body tracer uptake analysis enables visualization of spatial and temporal heterogeneity of PD-L1 *in vivo* in the tumor and lymph nodes and the change of expression during treatment, and can be linked to routine immunostaining procedures. [^18^F]-FDG imaging allows the detection of viable tumor at baseline and an early readout for response during treatment. In order to investigate feasibility of the addition of nivolumab treatment to standard surgery, patients will be monitored closely for adverse events and outcome data will be collected.

## Diagnostic work up

At the first visit of the patient at the outpatient clinic the study is discussed and information is handed out (day -5, see figure 1). During the initial oral inspection a diagnostic biopsy of the tumor is taken, in case no pathological conformation has been obtained already previously. During this phase patient is informed and signed Informed Consent obtained. A “diagnostic day” is scheduled in the standard workup, which routinely includes standard of care MRI and [^18^F]-FDG PET and, diagnostic CT neck, thorax and abdomen. [^18^F]BMS-986192 imaging is performed >24 h after or before [^18^F]-FDG PET (see below 3.3). Endoscopic examination with biopsies under general anesthesia (or if feasible in the outpatient clinic under local anesthesia) will be done after PET imaging (day 3±3, see figure 1) and additional biopsies of the tumor will be obtained. In case of discrepancies based on the [^18^F]-FDG and [^18^F]BMS-986192 uptake the patient will be asked for consent to additional biopsies.

## [^18^F]BMS-986192 and [^18^F]-FDG PET imaging acquisition

After inclusion and before panendoscopy, a [^18^F]BMS-986192 PET scan will be obtained for all patients. At VUmc, previous dynamic imaging with [^18^F]BMS-986192 has been performed to obtain data for kinetic modeling describing uptake, retention, and clearance of the tracer in tissue [58]. The steady state imaging timepoint for static whole body scanning has been set at 60-75 min after tracer injection. [^18^F]-FDG PET will be performed according to EANM guidelines [59]. To allow for ^18^F decay, [^18^F]-FDG PET and [^18^F]BMS-986192 PET will be performed with a minimum interval of 24 h time. PET-imaging will be done before panendoscopy (day 1 ±3, figure 1) and 21 days after nivolumab treatment (day 24±3, figure 1).

## Treatment with nivolumab

On day 3 ±3, patients will be treated with a single flat dose of 480mg IV. Details on preparation, handling and dispensing info are provided in section 5.6. Product description information is provided separately [32, 57].

## PET guided additional biopsies

Up to two additional biopsies will be taken when possible with acceptable additional risk in case the PET scan shows heterogeneous and/or discrepant uptake between tumor lesions of the same patient. In this way imaging [^18^F]BMS-986192 PET will aid further validation of PD-L1 IHC.

## Response evaluation

Patient will have a standard MRI of head and neck at screening (day -5-0, figure 1) and just before surgery (day 24 ±3-27, figure 1) to evaluate the response to nivolumab.

## Surgery

Primary tumor resection and (bilateral) cervical lymph node dissection with flap reconstruction will be performed after the immunotherapy. In general Dutch guidelines dictate that 80% of patients should start (surgical) treatment no later than 30 days after initial visit, depending on needed additional workup on the basis of e.g. PET-CT or to optimally prepare the patient for surgery. In preparation for surgery, special consideration must be taken when determining the resection margins- as nivolumab treatment may result in changes in tumor structure or volume. If deemed necessary by the operating surgeon, the tumor will be pre-operatively tattooed. The tattooed area will be removed during surgery. Furthermore, imaging with MRI is scheduled (see above) within 7 days before surgery to assess any changes. The immediate post-operative course lasts usually approximately 2 weeks during which hospital stay the patients will be monitored very closely for adverse events, including deviations from the usual recovery. Observations will be listed in the patient files. The tracheotomy cannula is normally removed on 5-7 days post-surgery.

## Post-surgery follow-up

Patients will be followed according to standard care during 12 months. The follow-up includes standard oral inspection and symptom directed physical examination. This will be combined with laboratory assessment to check for immune-related adverse events. In case the pathology report identifies tumor in the surgical margins and/or significant additional risk factors for recurrence, patients will continue treatment with (chemo)radiotherapy according to the Dutch Guidelines. Decision for additional treatment will be taken in the multidisciplinary tumor board. Additional treatments will be registered.

## Blood measurements: immunomonitoring and cytokine analysis

To ascertain if any relationship exists between observed PET image patterns and pre- and on-treatment immune status, polychromatic flowcytometric analyses of lymphoid and myeloid subsets in peripheral blood will be performed (for timepoints see section 7.11). Heparinized blood (40 ml) will be taken and PBMC isolated. The following 8-12 marker panels will be run on a BD LSRFortessa (with appropriate FMO controls): 1) Treg/effector T cell activation panel with among others: CD3, CD4, CD8, CD25, CD127, CD45RA, Ki67, FoxP3, CTLA-4, PD-1, CD27; 2) Dendritic cell panel: CD1c/BDCA-1, CD202/BDCA-2, CD141/BDCA-3, CD14, CD11c, CD16, CD19, CD40, CD80, PD-L1; 3) Myeloid-derived suppressor cell panel: CD11b, CD14, CD33, CD3/19/56 (Lin), HLA-DR, CD15, CD16, PD-L1. By performing these analyses at the indicated time points, we will gain unique insight in the kinetics of T cell activation following PD-1 blockade and pre- and on-treatment immune effector/suppressor subset profiles that may serve as biomarkers for clinical response and/or outcome, as we previously showed in a trial of combined ipilimumab and Prostate GVAX immunotherapy [60]. Plasma will also be stored and tested for cytokine content or seroreactivity at a later date.

## Plasma vesicle miRNAs

Exploratory blood-based biomarkers (plasma vesicle miRNAs) analysis will be performed. (for timepoints see section 7.11). Samples are immediately processed and plasma sample aliquots will be stored at -80°C until further exosome isolation and miRNA profiling.

## Tumor biopsy analysis

### Immunomonitoring: IHC

Tumor tissue from newly obtained FFPE material will be analyzed for PD-L1 (immunohistochemistry using the PDL-1 22C3 monoclonal antibody) and PD-1. Additional (in-house performed) investigational IHC analyses will include tumor and TIL PD-1 and PD-L2 expression, as well as explorative markers; CD3, CD4, CD8, FoxP3, IL10, MHC class I and II, and Ki67 double staining to ascertain effector T cell activation and proliferation in the tumor microenvironment as reported by [61].

### Genomic profiling

The DNA of biopsies of the surgical specimen will be analyzed for mutations and other genetic alterations by DNAseq and the number of neoantigens. DNA will be sheared by Covaris and we will use a SeqCap EZ HyperCap Workflow for library preparation. Paired end sequencing will be performed on an Illumina platform. Reads will be groomed and mapped to the human genome. Copy number profiles will be determined by QDNAseq [62] and somatic mutations will be called by a pipeline of four calling algorithms. RNAsequencing will be performed using the TruSeq RNA prepkit vs2 according to protocol and data analysed by routine pipelines at VUmc. Analysis of neoantigens will be performed with the neoantigen R package [63] and other algorithms using predefined cut-offs and in relation to the patient HLA subtype [64].


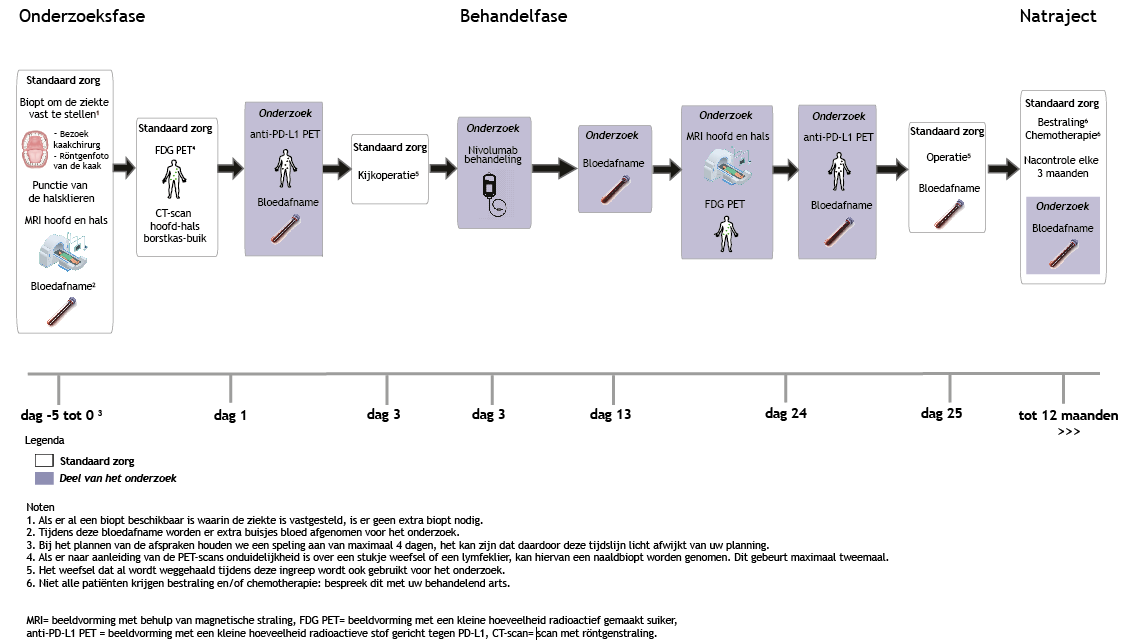
 Figure 1 Study Diagram for Patient

#

# STUDY POPULATION

Male/female subjects with locally advanced stage III/IV oral cancer scheduled for resection of the primary tumor and cervical lymph nodes, followed by postoperative radiotherapy or chemoradiation with curative intent, will be enrolled.

## Inclusion criteria

In order to be eligible to participate in this study, a subject must meet all of the following criteria:

1. Have a histologically confirmed diagnosis of locally advanced oral cancer (stage III/IV) which is planned for treatment with curative intent including surgical resection.
2. Be willing and able to provide written informed consent/assent for the trial.
3. Be ≥ 18 years of age on day of signing informed consent.
4. Must agree to provide tissue from fresh tumor biopsy pretreatment and from the surgical resection material to determine the actual PD-L1 status and perform immunomonitoring/DNA/RNA profiling.
5. Willing to allow up to two additional biopsies when baseline [^18^F]BMS-986192 PET /[^18^F]-FDG PET scans show heterogeneous and/or discrepant uptake.
6. Have a performance status of 0-1 on the ECOG Performance Scale ([Appendix 2](#_APPENDIX_2:_Eastern)).
7. Demonstrate adequate organ function as defined in table 1. All screening labs should be performed within 2 weeks before any study imaging procedures are performed.

Table 1 Adequate Organ Function Laboratory Values

| **System** | **Laboratory Value** |
| --- | --- |
| **Hematological** |  |
| White blood cell count (WBC) | ≥2,000 /mcL |
| Absolute neutrophil count (ANC) | ≥1,500 /mcL |
| Platelets | ≥100,000 / mcL |
| Hemoglobin | ≥9 g/dL or ≥5.6 mmol/L |
| **Renal** |  |
| Serum creatinine **OR**  Measured or calculated creatinine clearance  (GFR can also be used in place of creatinine or CrCl) | ≤1.5 X upper limit of normal (ULN) **OR**  ≥30 mL/min for subject with creatinine levels > 1.5 X institutional ULN |
| **Hepatic** |  |
| Serum total bilirubin | Total Bilirubin ≤ 1.5 x ULN (except subjects with Gilbert Syndrome, who can have total bilirubin < 3.0 mg/dL) |
| AST (SGOT) and ALT (SGPT) | ≤ 3 X ULN |

1. Women of childbearing potential (WOCBP) must use appropriate method(s) of contraception during the study and for 23 weeks after the last dose of nivolumab. Women who are not of childbearing potential (i.e. who are postmenopausal or surgically sterile) as well as azoospermic men do not require contraception ([Appendix 4](#Appendix4))
2. Men who are sexually active with WOCBP must use any contraceptive method with a failure rate of less than 1% per year. Men receiving nivolumab and who are sexually active with WOCBP will be instructed to adhere to contraception during the study and for 31 weeks after the last dose of nivolumab ([Appendix 4](#Appendix4)).

## Exclusion criteria

A subject who meets any of the following criteria will be excluded from participation in this study:

1. Is currently participating in or has participated in a study of an investigational agent within 4 weeks of the first dose of treatment or has not recovered (i.e., ≤ Grade 1 or at baseline) from adverse events due to agents administered more than 4 weeks earlier.
2. Has a known current additional malignancy that is progressing or requires active treatment. Exceptions include basal cell carcinoma of the skin, squamous cell carcinoma of the skin, or in situ cervical cancer that has undergone potentially curative therapy or synchronous head and neck squamous cell carcinoma.
3. If subject received major surgery for any other reason, they must have recovered adequately from the toxicity and/or complications from the intervention prior to starting therapy.
4. Subjects with a condition requiring systemic treatment with either corticosteroids (> 10 mg daily prednisone equivalent) or other immunosuppressive medications within 14 days of day -5. Inhaled or topical steroids, and adrenal replacement steroid > 10 mg daily prednisone equivalent, are permitted in the absence of active autoimmune disease.
5. Has an active autoimmune disease requiring systemic steroid treatment within the past 3 months or a documented history of clinically severe autoimmune disease, or a syndrome that requires systemic steroids.
6. Has evidence of interstitial lung disease or active, non-infectious pneumonitis.
7. Has an active infection requiring systemic therapy.
8. Has a history or current evidence of any condition, therapy, or laboratory abnormality that might confound the results of the trial, interfere with the subject’s participation for the full duration of the trial, or is not in the best interest of the subject to participate, in the opinion of the treating investigator.
9. Has known psychiatric or substance abuse disorders that would interfere with cooperation with the requirements of the trial.
10. Is pregnant or breastfeeding, or expecting to conceive or father children within the projected duration of the trial, starting with the pre-screening or screening visit through 23 weeks after the last dose of trial treatment.
11. Has received prior therapy with an anti-PD-1, anti-PD-L1, anti-PD-L2, anti-CTLA-4 antibody, or any other antibody or drug specifically targeting T-cell costimulation or immune checkpoint pathways.
12. Has a known history of Human Immunodeficiency Virus infection with a detectable viral load. Patients with an undetectable load (<50 copies/ml) receiving adequate anti-retroviral therapy, are allowed to participate.
13. Has known active Hepatitis B or C.

## Sample size calculation

Since this is a pilot feasibility study to investigate safety and tolerability, [^18^F]BMS-986192 and [^18^F]-FDG PET imaging before and on treatment with nivolumab, a sample size of 15 evaluable patients has been chosen. The objective of this study is descriptive and hypothesis generating, so no formal statistical justification for the sample size is provided.

# PET IMAGING WITH [18F]BMS-986192 and [18F]-FDG AND NIVOLUMAB TREATMENT

## Radiolabeling of [18F]BMS-986192

[^18^F]BMS-986192 will be radiolabeled according to radiochemical procedures that have been developed at VUmc and BMS. A Good Manufacturing Practice (GMP) compliant method has been set up and [^18^F]BMS-986192 can be produced according to GMP guidelines under the GMP manufacturing license for radiopharmaceuticals of the department of Radiology and Nuclear Medicine. Please refer to the Investigational Medicinal Product Dossier (IMPD) for further details.

## [^18^F]BMS-986192 injection procedure

The injections will take place at the department of Nuclear Medicine & PET research. Before treatment with nivolumab, a tracer dose of 225 MBq ±10% of [^18^F]BMS-986192 will be injected as a bolus of up to 10 ml through an injector (Medrad International, Maastricht, The Netherlands) at 0.8 ml/s, after which the line will be flushed with 10 mL physiologic saline. A second injection of [^18^F]BMS-986192 will be given 21 ±3 days after treatment with nivolumab to the same schedule (see [Schedule of assessments 7.19](#_Schedule_of_Assessments)).

## [^18^F]BMS-986192/[^18^F]-FDG PET procedure

All PET scans will be performed on a Philips Ingenuity TF PET/CT scanner. Each acquisition will start with a 30 mAs low-dose CT scan to correct for attenuation of the subsequent emission scan. A static total body PET scan after injection of 225 MBq ±10% [^18^F]BMS-986192 will be acquired at 60-75 min post injection. Static acquisition consists of 10-12 bed positions, depending on the length of the patient, of 3 minutes each.

[^18^F]-FDG PET will be performed in compliance with European Association of Nuclear Medicine (EANM) 2.0 guidelines [59], before and 21 ±3 days after start of treatment with nivolumab. Briefly, patients will fast 6 hours prior to the radiotracer injection. Patients will be injected with 3 MBq/kg (± 10%) [^18^F]-FDG. After 60 min (± 5 min) a PET scan will be performed from skull base to mid-thigh. The dose of [^18^F]-FDG will be about 185 MBq for PET (adapted by weight). Residual activity in the syringe will be measured and subtracted from the injected dose before calculations are done.

[^18^F]-FDG-PET and [^18^F]BMS-986192 PET will be performed with a minimum interval of 24 h to allow sufficient decay of the remaining tracer.

## Radiation exposure

For the patient the radiation dose of administering 225 ±10% MBq of [^18^F]BMS-986192 or [^18^F]-FDG is expected to be around 4.5 mSv each. Low dose CT scans used for attenuation correction will give an additional dose of 3 mSv per scan. The dose attributed to study procedures is therefore expected to be 22-23 mSv. The total radiation exposure (including standard of care) is expected to be 39-41 mSv. After injection no shielding is required and the patient can go home immediately.

## Analysis of PET data

### [18F]BMS-986192 PET

All PET data will be normalized and corrected for randoms, tissue attenuation, decay, scatter and dead time. PET-CT data will be reconstructed with TF-OSEM, resulting in a transaxial spatial resolution of ~7 mm in the centre of the field of view. Volumes of interest (VOIs) will be defined using the CT images which are co-registered to the PET-CT data and using a threshold technique [59, 65, 66]. The maximum pixel value within the tumor (SUV_max_), the 3D SUV_peak_, a threshold defined volume of interest providing SUV_mean_ and a manually CT defined volume (SUV_CT_) will be derived for any lesion with adequate focal uptake (50-53).

### [^18^F]-FDG PET

Data-analysis will be done using in-house developed software, including SUV_max_, SUV_peak_, 70% VOI, 50% VOI, normalized for serum glucose and body weight. Metabolic tumor volume (MTV is defined with an isocontour VOI of 50% of SUV_peak_ with background correction) and total lesion glycolysis (TLG is defined as SUV_mean corrected for body weight_ times MTV). Early response will be categorized according to PERCIST [67].

## Nivolumab treatment

Patients will be treated with a single flat dose of 480 mg nivolumab IV. The infusion protocol can be found in the investigator brochure [32]. There are no pre-medications recommended for nivolumab. There will be no dose modifications allowed. Subjects will be carefully monitored for infusion reactions during nivolumab administration. If an acute infusion reaction is noted, subjects will be managed according to protocol Section 5.2.6.

### Nivolumab preparation, handling and dispensing

Preparation and administration of Nivolumab

1. Visually inspect the drug product solution for particulate matter and discoloration prior to administration. Discard if solution is cloudy, if there is pronounced discoloration (solution may have a pale-yellow color), or if there is foreign particulate matter other than a few translucent-to-white, amorphous particles.

*Note: Mix by gently inverting several times. Do not shake.*

1. Aseptically withdraw the required volume of nivolumab solution into a syringe, and dispense into an IV bag. If multiple vials are needed for a subject, it is important to use a separate sterile syringe and needle for each vial to prevent problems such as dulling of needle tip, stopper coring, repeated friction of plunger against syringe barrel wall. Do not enter into each vial more than once. Do not administer study drug as an IV push or bolus injection.
2. Add the appropriate volume of 0.9% Sodium Chloride Injection solution or 5% Dextrose Injection solution. It is acceptable to add nivolumab solution from the vials into an appropriate pre-filled bag of diluent.

*Note: Nivolumab infusion concentration must be at or above the minimum allowable concentration of 0.35 mg/mL. For greater detail, see the Investigator Brochure [32].*

*Note: It is not recommended that so-called “channel” or tube systems are used to transport prepared infusions of nivolumab.*

1. Attach the IV bag containing the nivolumab solution to the infusion set and filter.
2. At the end of the infusion period, flush the line with a sufficient quantity of approved diluents.

Handling and dispensing of nivolumab

The study drug should be stored in accordance with the environmental conditions (temperature, light, and humidity) as per product information and the Investigator Brochure and per local regulations [32]. It is the responsibility of the investigator to ensure that investigational product is only dispensed to study subjects. The investigational product must be dispensed only from official study sites by authorized personnel according to local regulations. If concerns regarding the quality or appearance of the study drug arise, the study drug should not be dispensed and contact BMS immediately.

Please see the Investigator Brochure version 13 for additional information on storage, handling, dispensing, and infusion information for nivolumab [32].

### Dose modifications

Dose reductions or dose escalations are not permitted.

### Toxicity management algorithms

Although patients will receive only a single dose of nivolumab, (immune-related) adverse events other than infusion-related reactions may occur. Subjects should receive appropriate supportive care measures as deemed necessary by the treating investigator including but not limited to the items outlined below:

Immuno-oncology (I-O) agents are associated with Adverse Events (AEs) that can differ in severity and duration than AEs caused by other therapeutic classes. Nivolumab is considered an I-O agent in this protocol. Management algorithms have been developed to assist investigators in assessing and managing the following groups of AEs: Gastrointestinal, Renal, Pulmonary, Hepatic, Endocrinopathies, Skin, Neurological. These algorithms concern mainly patients who are continuously treated with nivolumab. Nevertheless, in case immune –related events occur, the management algorithms that have been developed, should be followed. Details can be found in the [Appendix 3](#_APPENDIX_3:_Management).

### Discontinuation criteria

As nivolumab will only be administered once, no discontinuation criteria apply.

### Treatment of nivolumab related infusion reactions

Since nivolumab contains only human immunoglobulin protein sequences, it is unlikely to be immunogenic and induce infusion or hypersensitivity reactions. However, if such a reaction were to occur, it might manifest with fever, chills, rigors, headache, rash, pruritus, arthralgia’s, hypo- or hypertension, bronchospasm, or other symptoms of allergic-like reactions.

All Grade 3 or 4 infusion reactions will be reported as an SAE if criteria are met. Infusion reactions will be graded according to National Cancer Institute (NCI) Common Terminology for Adverse Events (CTCAE) version 5.0 guidelines.

Treatment recommendations are provided below:

For Grade 1 symptoms: (Mild reaction; infusion interruption not indicated; intervention not indicated). Remain at bedside and monitor subject until recovery from symptoms.

For Grade 2 symptoms: (Moderate reaction requires therapy or infusion interruption but responds promptly to symptomatic treatment [e.g., antihistamines, non-steroidal anti-inflammatory drugs, narcotics, corticosteroids, bronchodilators, IV fluids]; prophylactic medications indicated for 24 hours).

Stop the nivolumab infusion, begin an IV infusion of normal saline, and treat the subject with diphenhydramine 50 mg IV (or equivalent) and/or paracetamol 325 to 1000 mg (acetaminophen); remain at bedside and monitor subject until resolution of symptoms. Corticosteroid or bronchodilator therapy may also be administered as appropriate. If the infusion is interrupted, then restart the infusion at 50% of the original infusion rate when symptoms resolve; if no further complications ensue after 30 minutes, the rate may be increased to 100% of the original infusion rate. Monitor subject closely. If symptoms recur then no further nivolumab will be administered at that visit. Administer diphenhydramine 50 mg IV, and remain at bedside and monitor the subject until resolution of symptoms. The amount of study drug infused must be recorded on the electronic case report form (eCRF). The following prophylactic premedications are recommended in case a second infusion the next day seems appropriate (in case < 25% of nivolumab has been administered): diphenhydramine 50 mg (or equivalent) and/or paracetamol 325 to 1000 mg (acetaminophen) should be administered at least 30 minutes before additional nivolumab administrations. If necessary, corticosteroids (recommended dose: up to 25 mg of IV hydrocortisone or equivalent) may be used.

For Grade 3 or Grade 4 symptoms:
Severe reaction, Grade 3: prolonged [ie, not rapidly responsive to symptomatic medication and/or brief interruption of infusion]; recurrence of symptoms following initial improvement; hospitalization indicated for other clinical sequelae [eg, renal impairment, pulmonary infiltrates]).
Grade 4: (life threatening; pressor or ventilatory support indicated). Immediately discontinue infusion of nivolumab. Begin an IV infusion of normal saline, and treat the subject as follows. Recommend bronchodilators, epinephrine 0.2 to 1 mg of a 1:1,000 solution for subcutaneous administration or 0.1 to 0.25 mg of a 1:10,000 solution injected slowly for IV administration, and/or diphenhydramine 50 mg IV with methylprednisolone 100 mg IV (or equivalent), as needed. Subject should be monitored until the investigator is comfortable that the symptoms will not recur. Nivolumab will be permanently discontinued. Investigators should follow their institutional guidelines for the treatment of anaphylaxis. Remain at bedside and monitor subject until recovery from symptoms.

In the case of late-occurring hypersensitivity symptoms (eg, appearance of a localized or generalized pruritus within 1 week after treatment), symptomatic treatment may be given (eg, oral antihistamine, or corticosteroids).

# ENDPOINTS

## Main study parameters/endpoints

PET Imaging

[^18^F]BMS-986192 and [^18^F]-FDG SUV_max_, SUV_mean,_ SUV_peak_ will be measured in all tumor lesions and (enlarged) lymph nodes using manually drawn volumes of interest (VOI) before and on treatment. For [^18^F]BMS-986192 manually drawn VOIs will also be calculated for liver, kidneys, lungs, spleen and left ventricle of the heart.

(Serious) adverse events

We will pay special interest to all adverse events during the study, in order to obtain information on safety and tolerability of neoadjuvant nivolumab in patients with locally advanced oral cancer who are planned for treatment with curative intent including surgery.

## Secondary study parameters/endpoints

#### Heterogeneity analysis

#### Interpatient, intrapatient and intratumor SUV heterogeneity.

Correlation between both PET scans, and between imaging and response

The correlation between SUV for [^18^F]BMS-986192 and [^18^F]-FDG baseline and on treatment and early response evaluation with [^18^F]-FDG.

Correlation between PET data and Blood/Tissue markers

The correlation between continuous values as SUV and the categorical variables tissue PD-1 and PD-L1 IHC will be assessed for each tumor lesion. Correlation with immuno-monitoring analysis in tumor tissue/lymph nodes and blood, plasma vesicle miRNAs from blood, as well as DNA/RNA profiling in tumor tissue at baseline and at surgery will be assessed.

# STUDY PROCEDURES

The Schedule of assessments (see section [7.19](#_7.19_Schedule_of)) summarizes the study procedures to be performed at each visit. Individual study procedures are described in detail below. It may be necessary to perform these procedures at unscheduled time points if deemed clinically necessary by the investigator. Furthermore, additional evaluations/testing may be deemed necessary for reasons related to subject safety. In some cases, such evaluation/testing may be potentially sensitive in nature (e.g., HIV, Hepatitis C, etc.), and thus local regulations may require that additional informed consent be obtained from the subject. In these cases, such evaluations/testing will be performed in accordance with those regulations.

## Informed Consent

The investigator must obtain documented consent from each potential subject prior to participating in a clinical trial. Consent must be documented by the subject’s dated signature or by the subject’s legally acceptable representative’s dated signature on a consent form along with the dated signature of the person conducting the consent discussion. A copy of the signed and dated consent form should be given to the subject. The initial informed consent form, any subsequent revised written informed consent form and any written information provided to the subject must receive the IRB/ERC’s approval/favorable opinion in advance of use. The subject or his/her legally acceptable representative should be informed in a timely manner if new information becomes available that may be relevant to the subject’s willingness to continue participation in the trial. The communication of this information will be provided and documented via a revised consent form or addendum to the original consent form that captures the subject’s dated signature or by the subject’s legally acceptable representative’s dated signature.

Specifics about a trial and the trial population will be added to the consent form template at the protocol level. The informed consent will adhere to IRB/ERC requirements and applicable laws and regulations.

## Inclusion/exclusion criteria

All inclusion and exclusion criteria will be reviewed by the investigator or qualified designee to ensure that the subject qualifies for the trial.

## Medical history

A medical history will be obtained by the investigator or qualified designee. Medical history will include all active conditions, and any condition diagnosed within the prior 10 years that are considered to be clinically significant by the Investigator.

## Prior medications

The investigator or qualified designee will review prior medication use, including any protocol-specified washout requirement, and record prior medication taken by the subject within 28 days before starting the trial.

## Concomitant medications

The investigator or qualified designee will record medication, if any, taken by the subject during the trial. All medications related to reportable Serious Adverse Events (SAEs) should be recorded as defined in section [8.1](#_AEs,_SAEs_and).

## Disease details

The investigator or qualified designee will obtain prior and current details regarding disease status.

## Assignment of screening number

All consented subjects will be given a unique screening number that will be used to identify the subject for all procedures that occur prior to the PET scans and nivolumab treatment. The screening assignment will start with the term NeoNivo followed by the number of inclusion (e.g. NeoNivo001).

## Adverse event monitoring

The investigator or qualified designee will assess each subject to evaluate for potential new or worsening AEs as specified in the Trial Flow Chart and more frequently if clinically indicated. Adverse experiences will be graded and recorded throughout the study according to NCI CTCAE Version 5.0 Toxicities will be characterized in terms regarding seriousness, causality, toxicity grading, and action taken with regard to trial treatment.

## (Full) physical exam

The investigator or qualified designee will perform a complete physical exam during the screening period and during follow up. Clinically significant abnormal findings should be recorded as medical history. Between screening and surgery, a directed physical exam as clinically indicated will be performed.

## Vital signs

The investigator or qualified designee will take vital signs at screening, prior to the administration of nivolumab and at first follow up visit as specified in the Trial Flow Chart. Vital signs should include temperature, pulse, respiratory rate, weight and blood pressure. Height will be measured at screening only.

## Laboratory procedures/assessments and blood collection

Details regarding specific laboratory procedures/assessments to be performed in this trial are provided below. The total amount of blood to be collected over the course of the trial (from pre-trial to post-trial visits), including approximate blood volumes collected per visit and by sample type per subject will be 328 ml.

For laboratory tests for hematology, chemistry and others, specified in Table 2, 1x7 ml EDTA tube and 1x7 ml Heparin Gel tube will be taken at screening and at follow-up. For WOCBP an additional 1x7 ml serum gel tube will be taken at screening for β-HCG measurement. Laboratory tests for screening should be performed within 14 days prior to the first study procedure.

**Table 2** Laboratory Tests

| **Hematology** | **Chemistry** | **Other** |
| --- | --- | --- |
| Hematocrit | Albumin | Free thyroxine (T4) |
| Hemoglobin | Alkaline phosphatase | Thyroid stimulating hormone (TSH) |
| Platelet count | Alanine aminotransferase (ALT) |  |
| Total WBC | Aspartate aminotransferase (AST) | Blood for correlative studies |
|  | Lactate dehydrogenase (LDH) |  |
|  | Amylase |  |
|  | Lypase |  |
|  | Creatinine |  |
|  | Calcium |  |
|  | Glucose |  |
|  | Phosphorus |  |
|  | Potassium |  |
|  | Sodium |  |
|  | Chloride |  |
|  | Magnesium |  |
|  | Total Bilirubin |  |

Peripheral blood samples for the purpose of immunomonitoring, genomic profiling and plasma vesicle miRNAs will be collected at the following timepoints:

T1 = baseline, before first [^18^F]BMS-986192 PET scan

T2 = 10 days after nivolumab, same day as check-up visit

T3 = 21 days after nivolumab, before second [^18^F]BMS-986192 PET scan

T4 = at follow-up (specified per analysis below).

For immuno-monitoring heparinized blood (4x10ml) will be taken at each of these time points and PBMCs isolated. Follow-up samples will be collected at 3 months after surgery.

For plasma vesicle miRNA analysis EDTA blood (12 ml) blood will be drawn at the same timepoints and at follow-up at 1 and 3 months post-surgery and at progression of disease (in case this occurs after the T4 (6 months) time point).

For genomic analysis, 1x7ml EDTA will be taken at screening for germ line DNA analysis

Total blood collection

Standard of care blood collection: 70ml

Study blood collection: = 258 ml

## Tumor imaging and assessment of disease

Besides the [^18^F]BMS-986192 and [^18^F]-FDG PET scans, a baseline MRI of head and neck will be made for baseline tumor measurements. Early response evaluation with MRI will be performed just before surgery (day 24 ±3, [Figure 1](#Figure1)). As included in standard of care, a diagnostic CT of neck, thorax and abdomen will be performed before treatment.

## Surgery

As described in section [3.6](#_Surgery).

## Adjuvant treatment

Adjuvant treatment (radiotherapy alone or in combination with chemotherapy or biotherapy with cetuximab) will be decided upon during the multidisciplinary board meetings, as indicated according to current guidelines. Treatment details will be registered and adverse events will be monitored.

## Follow-up visits

Visit requirements are outlined in section [7.19: Schedule of assessments](#_7.19_Schedule_of). Specific procedure-related details are provided above in [section 7: Study Procedures](#_STUDY_PROCEDURES). Subjects will have follow-up every 3 months until 12 months post-surgery, withdrawing consent, becoming lost to follow-up or the start of new anti-neoplastic therapy.

## Tissue sampling

Baseline tissue sampling will be obtained at panendoscopy, and a second tissue sampling will be obtained at surgey (after nivolumab treatment). Tumor tissue biopsies from the primary tumor and (affected) lymph nodes obtained at panendoscopy or surgical resection will be formalin fixed and fresh frozen .

When the [^18^F]BMS-986192 PET scan show heterogeneous uptake between tumor lesions in individual patients, or [^18^F]BMS-986192 PET and [^18^F]-FDG show discrepant results, up to two additional biopsies are allowed, if the area can be safely reached. If these discrepant results pertain to an area of interest that is already determined for surgical resection, a titanium marker is allowed to be placed before surgery under ultrasound guidance, if the area can be safely reached. The procedure for marker placement resembles that of taking a biopsy and holds similar risk.

## Screening period

During the short screening period (day -5 – 0) potential subjects will be evaluated to determine that they fulfill the entry requirements as set forth in [section 4](#_STUDY_POPULATION).

Written consent must be obtained prior to performing any protocol specific procedure. Results of a test performed prior to the subject signing consent as part of routine clinical management are acceptable in lieu of a screening test if performed within 30 days of study start, with the exception of laboratory test which should be performed within 14 days before study start. Screening procedures are to be completed prior to the first study procedure. Subjects may be rescreened after initially failing to meet the inclusion/exclusion criteria. Results from assessments performed during the initial screening period are acceptable in lieu of repeating a screening test if performed within the specified time frame and the results meet the inclusion/exclusion criteria. The screening period is estimated at 5 days, but may be elongated, only if standard of care procedures are delayed. Study procedures will not delay standard of care planning.

## Withdrawal/discontinuation

When a subject discontinues / withdraws prior to trial completion, all applicable activities scheduled for the final trial visit should be performed at the time of discontinuation. Any adverse events which are present at the time of discontinuation / withdrawal should be followed and assessed as described in [section 8](#_SAFETY_REPORTING).

### Withdrawal of individual subjects

Subjects may withdraw consent at any time for any reason or be dropped from the trial at the discretion of the investigator should any untoward effect occur. In addition, a subject may be withdrawn by the investigator if enrollment into the trial is inappropriate, the trial plan is violated, or for administrative and/or other safety reasons.

### Replacement of individual subjects after withdrawal

Subjects that fail to successfully undergo the PET scans will be replaced. In addition, in case baseline [^18^F]-FDG PET or diagnostic CT of neck, thorax and abdomen is suspicious for distant metastases the suspicious lesion will be biopsied. In case of proven metastases before the start of treatment with nivolumab, the patient will fall out of the study and will be replaced.

### Premature termination of the study

Early trial termination will be the result of the criteria specified below:

1. Incidence or severity of adverse drug reaction in this or other studies indicates a potential health hazard to subjects. Specifically, adverse events during treatment with nivolumab, during surgery and during standard adjuvant treatment (if indicated) will be monitored. In case of (prolonged) grade 4 or grade 5 toxicity, the investigators can temporarily halt the study for further analyses.

2. Quality or quantity of data recording is inaccurate or incomplete.

3. Poor adherence to the protocol and regulatory requirements.

## Schedule of assessments

|  |  | **Screening** | **Imaging I / Work-up** | | | | | | | | | **Panendoscopy** | | | **Nivolumab** | | **Check-up** | **Imaging II** | | **Surgery** | **3-monthly FU^1^** |
| --- | --- | --- | --- | --- | --- | --- | --- | --- | --- | --- | --- | --- | --- | --- | --- | --- | --- | --- | --- | --- | --- |
|  | Scheduling Window (days) | -5 – 0^11^ | 1 ±3 | | | | | | | | | 3 ±3 | | | 3±3 | | 13±3 | 24 ±3 | | 25 ±4 | >> |
|  | **Administrative Procedures** |  |  |  |  |  |  |  |  |  |  |  |  |  |  |  |  |  |  |  |  |
|  | Pre-screening Consent | X |  |  |  |  |  |  |  |  |  |  |  |  |  |  |  |  |  |  |  |
|  | Informed Consent | X |  |  |  |  |  |  |  |  |  |  |  |  |  |  |  |  |  |  |  |
|  | Elegibility Criteria | X |  |  |  |  |  |  |  |  |  |  |  |  |  |  |  |  |  |  |  |
|  | Demographics and Medical History | X |  |  |  |  |  |  |  |  |  |  |  |  |  |  |  |  |  |  | X |
|  | Prior and Concomitant Medication Review | X |  |  |  |  |  |  |  |  |  |  |  |  |  |  |  |  |  | X | X |
|  | **Imaging** |  |  |  |  |  |  |  |  |  |  |  |  |  |  |  |  |  |  |  |  |
|  | PET - [18F]-BMS-986192^2^ |  | X | | | | | | | | |  |  |  |  |  |  | X | |  |  |
|  | PET - [18F]-FDG^2^ | X | | | | | | | | | |  |  |  |  |  |  | X | |  |  |
|  | MRI Head-Neck | X |  | | | | | | | | |  |  |  |  |  |  | X | |  |  |
|  | Diagnostic CT-Neck-Thorax-Abdomen | X | | | | | | | | | |  |  |  |  |  |  |  |  |  |  |
|  | **Clinical Procedures/Assessments** |  |  |  |  |  |  |  |  |  |  |  |  |  |  |  |  |  |  |  |  |
|  | Directed Physical Examination | X |  |  |  |  |  |  |  |  |  |  |  |  | X | | X |  |  | X | X |
|  | Diagnostic Biopsy^3^ | X |  |  |  |  |  |  |  |  |  |  | | |  |  |  |  |  |  |  |
|  | Panendoscopy including biopsies |  |  |  |  |  |  |  |  |  |  | X | | |  |  |  |  |  |  |  |
|  | Review Adverse Events^4^ |  | X | | | | | | | | | X | | | X | | X | X | | X | X^5^ |
|  | Full Physical Examination | X |  |  |  |  |  |  |  |  |  |  |  |  |  |  |  |  |  |  | X |
|  | Vital Signs and Weight | X |  |  |  |  |  |  |  |  |  |  |  |  | X | |  |  |  | X | X |
|  | ECOG Performance Status | X |  |  |  |  |  |  |  |  |  |  |  |  | X | |  |  |  | X | X |
|  | MKA-OPT | X | | | | | | | | | |  |  |  |  |  |  |  |  |  |  |
|  | MKA extractions |  |  |  |  |  |  |  |  |  |  | X | | |  |  |  |  |  |  |  |
|  | Echo-FNAC | X | | | | | | | | | |  |  |  |  |  |  |  |  |  |  |
|  | Nivolumab treatment |  |  |  |  |  |  |  |  |  |  |  |  |  | X | |  |  |  |  |  |
|  | **Laboratory Procedures/Assessments** |  |  |  |  |  |  |  |  |  |  |  |  |  |  |  |  |  |  |  |  |
|  | Pregnancy Test^6^ | X |  |  |  |  |  |  |  |  |  |  |  |  |  |  |  |  |  |  |  |
|  | Complete Blood Count (CBC) | X |  |  |  |  |  |  |  |  |  |  |  |  |  |  | X |  |  | X | X |
|  | Comprehensive Serum Chemistry Panel^7^ | X |  |  |  |  |  |  |  |  |  |  |  |  |  |  | X |  |  | X | X |
|  | **Tumor Tissue and Blood collection** |  |  |  |  |  |  |  |  |  |  |  |  |  |  |  |  |  |  |  |  |
|  | Tissue collection for genomic profiling |  |  | | | | | | | | |  | | |  |  |  |  |  | X^8^ |  |
|  | Tissue collection for Immunohistochemistry |  |  | | | | | | | | | X^8^ | | |  |  |  |  |  | X^8^ |  |
|  | Tissue collection for flow cytometry |  |  | | | | | | | | |  | | |  |  |  |  |  | X^8^ |  |
|  | Blood for Immunophenotyping and plasma vesicle miRNA’s |  | X^9^ | | | | | | | | |  | | |  | | X | X^9^ | |  | X^10^ |
|  |  |  |  |  |  |  |  |  |  |  |  |  |  |  |  |  |  | |  |  |  |
|  | Standard of Care |  |  |  |  |  |  |  |  |  |  |  |  |  |  |  |  | |  |  |  |
|  | Study procedure |  |  |  |  |  |  |  |  |  |  |  |  |  |  |  |  | |  |  |  |

1. Follow up will end 12 months post-surgery, or earlier when withdrawing consent, becoming lost to follow-up or at the start of new anti-neoplastic therapy.
2. [^18^F]BMS-986192 PET and [^18^F]-FDG PET need to occur with an interval of min. 24h.
3. Only if diagnosis has not been histologically confirmed at presentation
4. This includes AEs, SAEs and SUSARs, as described in section 8.1
5. Adverse events will be monitored until 100 days after nivolumab treatment.
6. Only for women of childbearing potential.
7. This includes Albumin, Akaline phosphatase, Alanine aminotransferase (ALT), Aspartate aminotransferase (AST), Lactate dehydrogenase (LDH), Amylase, Lipase, Creatinine, Calcium, Glucose, Phosphorus, Potassium, Sodium, Chloride, Magnesium, Total Bilirubin, Free Thyroxine (T4), Triiodothyronine (T3), Thyroid Stimulating Hormone (TSH). 1 tube of 6ml EDTA (EK) and one tube of 6ml Heparin Gel (HG) need to be collected.
8. Tissues are collected during standard procedures.
9. Blood is to be withdrawn before [18F]-BMS-986192 administration.
10. For plasma vesicle miRNA analysis: blood collection at 1 and 3 months post-surgery as well as at time of progression of disease. For immunophenotyping 4x10ml has to be collected only once, at 3 months after surgery.
11. The screening period is estimated at 5 days, but may be elongated, only if standard of care procedures are delayed.

# SAFETY REPORTING

Section 10 WMO event

In accordance to section 10, subsection 1, of the Dutch law “Wet Medisch-wetenschappelijk Onderzoek (WMO)”, the investigator will inform the subjects and the reviewing accredited medical ethical committee (medische ethische toetsingscommissie, METC) if anything occurs, on the basis of which it appears that the disadvantages of participation may be significantly greater than was foreseen in the research proposal. The study will be suspended pending further review by the accredited METC, except insofar as suspension would jeopardise the subjects’ health. The investigator will take care that all subjects are kept informed.

## AEs, SAEs and SUSARs

### Adverse events (AEs)

Adverse events are defined as any undesirable experience occurring to a subject during the study, whether or not considered related to radioactive tracers or nivolumab treatment. An investigator who is a qualified physician will evaluate all adverse events according to the NCI Common Terminology for Adverse Events (CTCAE), version 5.0. Any adverse event which changes CTCAE grade over the course of a given episode will have each change of grade recorded on the adverse event case report forms/worksheets.

All adverse events regardless of CTCAE grade must also be evaluated for seriousness.

### Serious adverse events (SAEs)

A serious adverse event is any untoward medical occurrence or effect that at any dose:

- results in death;
- is life threatening (at the time of the event);
- requires hospitalisation or prolongation of existing inpatients’ hospitalisation;
- results in persistent or significant disability or incapacity;
- is a congenital anomaly or birth defect;
- Any other important medical event that may not result in death, be life threatening, or require hospitalization, may be considered a serious adverse experience when, based upon appropriate medical judgement, the event may jeopardize the subject or may require an intervention to prevent one of the outcomes listed above.
- Potential drug induced liver injury (DILI) is also considered an important medical event.
- Complications during or after surgical procedures and during adjuvant treatment are also considered an important medical event.
- Suspected transmission of an infectious agent (e.g., pathogenic or nonpathogenic) via the study drug is an SAE.

Although pregnancy, overdose, and cancer are not always serious by regulatory definition, these events must be handled as SAEs.

### Suspected Unexpected Serious Adverse Reactions (SUSARs)

Adverse reactions are all untoward and unintended responses to an investigational product related to any dose administered.

Unexpected adverse reactions are SUSARs if the following three conditions are met:

1. the event must be serious;
2. there must be a certain degree of probability that the event is a harmful and an undesirable reaction to nivolumab, regardless of the administered dose;
3. the adverse reaction must be unexpected, that is to say, the nature and severity of the adverse reaction are not in agreement with the product information as recorded in the Investigator Brochure [32].

The sponsor will report expedited the following SUSARs through the web portal *ToetsingOnline* to the METC:

- SUSARs that have arisen in the clinical trial that was assessed by the METC;
- SUSARs that have arisen in other clinical trials of the same sponsor and with the same medicinal product, and that could have consequences for the safety of the subjects involved in the clinical trial that was assessed by the METC.

The remaining SUSARs are recorded in an overview list (line-listing) that will be submitted once every half year to the METC. This line-listing provides an overview of all SUSARs from the two investigational products (PET tracer and nivolumab), accompanied by a brief report highlighting the main points of concern.

The expedited reporting of SUSARs through the web portal ToetsingOnline is sufficient as notification to the competent authority.

The sponsor will report expedited all SUSARs to the competent authorities in other Member States, according to the requirements of the Member States.

The expedited reporting will occur not later than 15 days after the sponsor has first knowledge of the adverse reactions. For fatal or life threatening cases the term will be maximal 7 days for a preliminary report with another 8 days for completion of the report.

### Potential Drug Induced Liver Injury (DILI)

Wherever possible, timely confirmation of initial liver-related laboratory abnormalities should occur prior to the reporting of a potential DILI event. All occurrences of potential DILIs, meeting the defined criteria, must be reported as SAEs. Potential drug induced liver injury is defined as:

1. ALT or AST elevation > 3 times upper limit of normal (ULN)

AND

1. Total bilirubin > 2 times ULN, without initial findings of cholestasis (elevated serum alkaline phosphatase)

AND

1. No other immediately apparent possible causes of AST/ALT elevation and hyperbilirubinemia, including, but not limited to, viral hepatitis, pre-existing chronic or acute liver disease, or the administration of other drug(s) known to be hepatotoxic.

### Laboratory Test Abnormalities

All laboratory test results captured as part of the study should be recorded following institutional procedures. Test results that constitute SAEs should be documented and reported as such.

The following laboratory abnormalities should be documented and reported appropriately:

- any laboratory test result that is clinically significant or meets the definition of an SAE
- any laboratory abnormality that required the subject to have study drug discontinued or interrupted
- any laboratory abnormality that required the subject to receive specific corrective therapy.

Progression of the cancer under study is not considered an adverse event unless it results in hospitalization or death.

### Complications during surgery

In recent studies administration of neoadjuvant nivolumab is associated with few side effects during surgery. In a cohort of 22 NSCLC patients, scheduled for surgical resection, patients received two two-weekly doses of 3 mg/kg nivolumab. No previously unreported toxic effects were observed. Treatment-related adverse events occurred in 5 of 22 patients, with only one grade 3 event (pneumonia). Furthermore, neoadjuvant nivolumab did not cause treatment-related surgical delays [30]. A similar study in HNSCC included 29 HNSCC patients eligible for surgical resection. Patients received 240 mg nivolumab on day 1 and on day 15 of the study, and surgery was performed on day 29±7. Preliminary results were presented at EMSO in 2017 [68]. No new safety signatures were defined. Grade 3-4 treatment-related adverse events occurred in 4 patients and consisted of glossodynia and lipase increase.

Based on these studies we do not expect any complications of special interest during surgery or adjuvant treatment.

## Serious Adverse Event Collection and Reporting

Following subject’s written consent to participate in the study, all SAEs, whether related or not related to one of the radioactive tracers, study procedure or nivolumab, must be collected, including those thought to be associated with protocol-specified procedures. All SAEs must be collected that occur within 100 days of discontinuation of dosing or the initiation of new anti-cancer therapy, whichever is earlier.

All SAEs must be collected that occur during the screening period. If applicable, SAEs must be collected that relate to any protocol-specified procedure (eg, additional tumor biopsy, PET scans). The investigator should report any SAE that occurs after these time periods that is believed to be related to nivolumab or protocol-specified procedures.

SAEs, whether related or not related to one of the radioactive tracers, study procedure or nivolumab, and pregnancies must be reported to BMS at the same time when reporting to METC within 24 hours. SAEs will be reported through the web portal *ToetsingOnline* to the accredited METC that approved the protocol.

SAEs must be recorded on a **SAE** form and pregnancies on a Pregnancy Surveillance Form via:

**SAE Email Address:**  Worldwide.Safety@BMS.com

**SAE Facsimile Number: +1** 609-818-3804

If only limited information is initially available, follow-up reports are required. (Note: follow-up SAE reports should include the same investigator term(s) initially reported.)

If an ongoing SAE changes in its intensity or relationship to nivolumab or if new information becomes available, a follow-up SAE report should be sent within 24 hours to BMS (or designee) using the same procedure used for transmitting the initial SAE report.

All SAEs should be followed to resolution or stabilization.

The Sponsor/Investigator (VUmc) will ensure that all SAEs in the clinical database are reported to BMS and any applicable health authority during the conduct of the study including periodic reconciliation.

VUmc shall ensure that all Study Personnel (including Investigator) report to BMS all adverse events and other reportable events in the course of the Study in accordance with Applicable Law and the Protocol. VUmc shall ensure that Study Personnel (including Investigator) perform case level reconciliation to confirm BMS has received all reports reported by Study Personnel. Study Personnel shall use the form of reconciliation report requested by BMS and shall e-mail all reconciliation reports to AEPBUSINESSPROCESS@BMS.com

Reconciliation shall be performed on a quarterly basis, unless otherwise agreed by BMS in writing.

All Study Personnel (including Investigator) shall complete any training reasonably requested by BMS relating to safety reporting, and shall provide evidence of, or certify to, the same, as reasonably requested by BMS.

## Pregnancy

If, following treatment with nivolumab, it is subsequently discovered that a study subject is pregnant or may have been pregnant at the time of investigational product exposure, including during at least 6 half-lives after product administration, the investigational product will be permanently discontinued.

The investigator must immediately notify Worldwide.Safety@BMS.com of this event via the Pregnancy Surveillance Form in accordance with SAE reporting procedures.

Follow-up information regarding the course of the pregnancy, including perinatal and neonatal outcome and, where applicable, offspring information must be reported on the Pregnancy Surveillance Form (provided upon request from BMS)

Any pregnancy that occurs in a female partner of a male study participant should be reported to BMS. Information on this pregnancy will be collected on the Pregnancy Surveillance Form.

## Overdose

An overdose is defined as the accidental or intentional administration of any dose of a product that is considered both excessive and medically important. All occurrences of overdose must be reported as an SAE.

## Other safety considerations

Any significant worsening noted during interim or final physical examinations, electrocardiograms, x‑rays, and any other potential safety assessments, whether or not these procedures are required by the protocol, should also be recorded as a nonserious or serious AE, as appropriate, and reported accordingly.

## Annual safety report

In addition to the expedited reporting of SUSARs, the sponsor will submit, once a year throughout the clinical trial, a safety report to the accredited METC, competent authority, and competent authorities of the concerned Member States.

This safety report consists of:

- a list of all suspected (unexpected or expected) serious adverse reactions, along with an aggregated summary table of all reported serious adverse reactions, ordered by organ system, per study;
- a report concerning the safety of the subjects, consisting of a complete safety analysis and an evaluation of the balance between the efficacy and the harmfulness of the medicine under investigation.

## Follow-up of adverse events

All AEs will be followed until they have abated, or until a stable situation has been reached, up until 100 days after administration of nivolumab. Depending on the event, follow up may require additional tests or medical procedures as indicated, and/or referral to the general physician or a medical specialist.

SAEs need to be reported till end of study within the Netherlands, as defined in the protocol.

# STATISTICAL ANALYSIS

## Analysis of PET data

SUV_max_, SUV_mean_ and SUV_peak_ (both tracers) will be measured for all tumor lesions and enlarged lymph nodes. For ^18^F-FDG also MTV and TLG will be determined.

#### Heterogeneity analysis

### Interpatient heterogeneity

Test-retest variability of PET using modern machines is ~15% (62-64). Therefore, uptake difference between patients (interpatient heterogeneity) is defined as SUV differences of 15% or greater.

### Intrapatient heterogeneity

Tracer uptake differences between lesions of the same patient depend on multiple aspects. These are both biological (e.g. the level of perfusion and target expression), as well as technical (e.g. partial volume and tumor motion effects). True biological differences between lesions that correlate with tumor biology are therefore difficult to define. Because of this, intrapatient uptake differences will be assessed qualitatively.

### Intratumor heterogeneity

Uptake heterogeneity within a tumor also depends on multiple aspects. It can be the result of biological differences, such as perfusion (the tracer is not able to reach some parts of the tumor), true target expression or tumor necrosis. Tumor motion effects also influence intratumor heterogeneity since PET acquisition is not respiratory gated.

Intratumor heterogeneity is defined as the ratio of SUV_max_ : SUV_mean_. The level will be calculated on a linear scale.

## Correlation between PET data and response

The correlation between the continuous variable SUV, the % change in SUV and TLG and the categorical variables response on imaging (according to RECIST 1.1 CR, PR, SD, PD and CMR, PMR, SMD and PMD according to PERCIST) and pathological response (according to the Mandard Classification System, Table 3) will be assessed.

**Table 3** Mandard Classification system for Tumor Regression Grading (TRG) [69, 70]

| **Descriptive** |  |  |
| --- | --- | --- |
| Complete regression | TRG 1 | No residual cancer cells |
| Subtotal | TRG 2 | Rare cancer cells |
| Partial | TRG 3 | Fibrosis outgrowing residual cancer |
| No regression | TRG 4 | Residual cancer outgrowing fibrosis |
|  | TRG 5 | Absence of regressive change |

## Correlation between PET data and Blood and Tissue markers

The correlation between the continuous variable SUV and the categorical variables tissue PD-1 and PD-L1 IHC will be assessed with the ANOVA test. The correlation between the continuous variable SUV and the categorical blood variables will be assessed with the T test.

# ETHICAL CONSIDERATIONS

## Regulation statement

The study will be conducted according to the principles of the Declaration of Helsinki (amended by the 64^th^ WMA General Assembly, Fortaleza, Brazil, October 2013) and in accordance with the Medical Research Involving Human Subjects Act (WMO).

## Recruitment and consent

Eligible patients will be identified at the outpatient department. The treating physician will ask if the patient can be approached for study participation. Each subject will be given a minimum of 24 h time to read the informed consent form and the opportunity to ask questions. Subsequently, subjects will be asked for permission. Patient consent and inclusion will be performed by a trained physician involved in the study.

## Benefits and risks assessment, group relatedness

- PET scans

No toxicity is expected from PET scans with tracer microdoses. The amount of [^18^F]BMS-986192 will be in the pico to nano molar quantity, far below the dose for a pharmacological effect. The additional PET scans implement a radiation burden of 22-23 mSv including the low dose CT for attenuation correction. Patients do not derive benefit from the PET scan results. Since there is a lack of a well performing predictive biomarker of response, the results of this imaging biomarker study can be of high interest for HNSCC patients that are eligible for anti-PD-(L)1 treatment in the future.

- Tumor biopsy

Additional biopsies will be taken in this study (after the baseline PET scans) in case the [^18^F]BMS-986192 or the [^18^F]-FDG PET scan show heterogeneous or discrepant uptake in individual patients. Although this is demanding for patients, tumor biopsies in cancer patients are considered safe with a low and manageable complication rate. These biopsies will be used to explain heterogeneous tracer uptake and relate the imaging results to that of the tissue biomarkers PD-1 IHC (locally) and PD-L1 IHC (BMS Dako assay), as well as immune monitoring outcomes.

- Marker placement

If these discrepant results pertain to an area of interest that is already determined for surgical resection, a titanium marker is placed before surgery under ultrasound guidance, if the area can be safely reached. This standard radiological procedure for marker placement resembles that of taking a biopsy and holds similar low risks.

- Blood withdrawal

By performing immunophenotyping on PBMC’s at the indicated time points unique insight will be gained in the kinetics of T cell activation following PD-1 blockade and pre- and on-treatment immune effector/suppressor subset profiles that may serve as biomarkers for clinical response and/or outcome, as we previously showed in a trial of combined ipilimumab and Prostate GVAX immunotherapy [60, 71, 72].

- Nivolumab treatment

See Section 5.6. The overall cure rate for locally advanced oral cancer is 50-60% with surgery and if indicated adjuvant (chemo)radiotherapy. Preliminary data have shown promising responses with neoadjuvant treatment with nivolumab or pembrolizumab. It is therefore not unlikely that patients derive benefit from this study. As can be found in the Investigator Brochure, the toxicity is manageable and the safety profile acceptable [32].

## Compensation for injury

The sponsor/investigator has a liability insurance which is in accordance with article 7 of the WMO.

The sponsor (also) has an insurance which is in accordance with the legal requirements in the Netherlands (Article 7 WMO). This insurance provides cover for damage to research subjects through injury or death caused by the study.

The insurance applies to the damage that becomes apparent during the study or within 4 years after the end of the study.

## Incentives

Patients will be compensated for travel costs and parking cost for study visits.

# ADMINISTRATIVE ASPECTS, MONITORING AND PUBLICATION

## Handling and storage of data and documents

The investigators will maintain adequate records, including signed patients informed consent forms and information on adverse events. These documents will be kept in a secured area with limited access. All records will be signed and dated by the investigators. All records will be retained for a period of 15 years following the date the entire clinical investigation is completed, terminated or discontinued. The confidentiality will be guaranteed and patients’ identification will be coded.

The code will start with the term NeoNivo followed by the number of inclusion (e.g. NeoNivo001). Patient data will be centralized by the coordinating investigator and kept under strict confidentiality.

## Handling and storage of body materials

Body materials that remain after the analyses described in this protocol will be stored in the biobank “Neoadjuvant nivolumab mondholtecarcinoom”, with reference number 2018.640. This biobank has been registered and approved by the review committee biobank (in Dutch: toetsingscommissie biobank) of the VUmc.

## Monitoring and Quality Assurance

This study will be monitored by appropriate staff from the Clinical Research Bureau (CRB), and may also be audited/inspected by an independent body and/or authorities. By agreeing to this protocol, the investigator agrees to co-operate fully with compliance checks by allowing access for authorised individuals to all relevant study documents.

## Amendments

Amendments are changes made to the research after a favourable opinion by the accredited METC has been given. All amendments will be notified to the METC.

## Start of study report

The sponsor/investigator will notify the accredited METC and the competent authority of the start of the study within a period of 15 days.

## Annual progress report

The sponsor/investigator will submit a summary of the progress of the trial to the accredited METC once a year. Information will be provided on the date of inclusion of the first subject, numbers of subjects included and numbers of subjects that have completed the trial, serious adverse events/ serious adverse reactions, other problems, and amendments.

## End of study report

The sponsor/investigator will notify the accredited METC and the competent authority of the end of the study within a period of 90 days. The end of the study is defined as the last patient’s last visit.

In case the study is ended prematurely, the sponsor will notify the accredited METC and the competent authority within 15 days, including the reasons for the premature termination.
Within one year after the end of the study, the investigator/sponsor will submit a final study report with the results of the study, including any publications/abstracts of the study, to the accredited METC and the Competent Authority.

## Public disclosure and publication policy

After the last patient entered the study and the results have been obtained and analysed, a study report will be written. The aim is to report the study findings in international peer-reviewed journals and to present the data at international meetings.

# REFERENCES

1. Disis, M.L., *Immune regulation of cancer.* J Clin Oncol, 2010. **28**(29): p. 4531-8.

2. Dong, H., et al., *Tumor-associated B7-H1 promotes T-cell apoptosis: a potential mechanism of immune evasion.* Nat Med, 2002. **8**(8): p. 793-800.

3. Sharpe, A.H. and G.J. Freeman, *The B7-CD28 superfamily.* Nat Rev Immunol, 2002. **2**(2): p. 116-26.

4. Brown, J.A., et al., *Blockade of programmed death-1 ligands on dendritic cells enhances T cell activation and cytokine production.* J Immunol, 2003. **170**(3): p. 1257-66.

5. Francisco, L.M., P.T. Sage, and A.H. Sharpe, *The PD-1 pathway in tolerance and autoimmunity.* Immunol Rev, 2010. **236**: p. 219-42.

6. Thompson, R.H., et al., *PD-1 is expressed by tumor-infiltrating immune cells and is associated with poor outcome for patients with renal cell carcinoma.* Clin Cancer Res, 2007. **13**(6): p. 1757-61.

7. Talmadge, J.E., M. Donkor, and E. Scholar, *Inflammatory cell infiltration of tumors: Jekyll or Hyde.* Cancer Metastasis Rev, 2007. **26**(3-4): p. 373-400.

8. Usubutun, A., et al., *Prognostic factors in renal cell carcinoma.* J Exp Clin Cancer Res, 1998. **17**(1): p. 77-81.

9. Deschoolmeester, V., et al., *Tumor infiltrating lymphocytes: an intriguing player in the survival of colorectal cancer patients.* BMC Immunol, 2010. **11**: p. 19.

10. Diez, M., et al., *Histopathologic prognostic score in colorectal adenocarcinomas.* Anticancer Res, 1998. **18**(1B): p. 689-94.

11. Galon, J., et al., *Type, density, and location of immune cells within human colorectal tumors predict clinical outcome.* Science, 2006. **313**(5795): p. 1960-4.

12. Hiraoka, N., *Tumor-infiltrating lymphocytes and hepatocellular carcinoma: molecular biology.* Int J Clin Oncol, 2010. **15**(6): p. 544-51.

13. Nobili, C., et al., *Prolonged survival of a patient affected by pancreatic adenocarcinoma with massive lymphocyte and dendritic cell infiltration after interleukin-2 immunotherapy. Report of a case.* Tumori, 2008. **94**(3): p. 426-30.

14. Hodi, F.S. and G. Dranoff, *The biologic importance of tumor-infiltrating lymphocytes.* J Cutan Pathol, 2010. **37 Suppl 1**: p. 48-53.

15. Kloor, M., *Lymphocyte infiltration and prognosis in colorectal cancer.* Lancet Oncol, 2009. **10**(9): p. 840-1.

16. Hillen, F., et al., *Leukocyte infiltration and tumor cell plasticity are parameters of aggressiveness in primary cutaneous melanoma.* Cancer Immunol Immunother, 2008. **57**(1): p. 97-106.

17. Lee, H.E., et al., *Prognostic implications of type and density of tumour-infiltrating lymphocytes in gastric cancer.* Br J Cancer, 2008. **99**(10): p. 1704-11.

18. Leffers, N., et al., *Prognostic significance of tumor-infiltrating T-lymphocytes in primary and metastatic lesions of advanced stage ovarian cancer.* Cancer Immunol Immunother, 2009. **58**(3): p. 449-59.

19. Nishimura, H., T. Honjo, and N. Minato, *Facilitation of beta selection and modification of positive selection in the thymus of PD-1-deficient mice.* J Exp Med, 2000. **191**(5): p. 891-8.

20. Yearley, J.H., et al., *PD-L2 Expression in Human Tumors: Relevance to Anti-PD-1 Therapy in Cancer.* Clin Cancer Res, 2017. **23**(12): p. 3158-3167.

21. Muller, T., et al., *PD-L1: a novel prognostic biomarker in head and neck squamous cell carcinoma.* Oncotarget, 2017. **8**(32): p. 52889-52900.

22. Liotta, F., et al., *Frequency of regulatory T cells in peripheral blood and in tumour-infiltrating lymphocytes correlates with poor prognosis in renal cell carcinoma.* BJU Int, 2011. **107**(9): p. 1500-6.

23. Sharpe, A.H., et al., *The function of programmed cell death 1 and its ligands in regulating autoimmunity and infection.* Nat Immunol, 2007. **8**(3): p. 239-45.

24. Seiwert, T.Y., et al., *Safety and clinical activity of pembrolizumab for treatment of recurrent or metastatic squamous cell carcinoma of the head and neck (KEYNOTE-012): an open-label, multicentre, phase 1b trial.* Lancet Oncol, 2016. **17**(7): p. 956-965.

25. Ferris, R.L., et al., *Nivolumab for Recurrent Squamous-Cell Carcinoma of the Head and Neck.* N Engl J Med, 2016. **375**(19): p. 1856-1867.

26. Bauml, J., et al., *Pembrolizumab for Platinum- and Cetuximab-Refractory Head and Neck Cancer: Results From a Single-Arm, Phase II Study.* J Clin Oncol, 2017. **35**(14): p. 1542-1549.

27. Chow, L.Q.M., et al., *Antitumor Activity of Pembrolizumab in Biomarker-Unselected Patients With Recurrent and/or Metastatic Head and Neck Squamous Cell Carcinoma: Results From the Phase Ib KEYNOTE-012 Expansion Cohort.* J Clin Oncol, 2016. **34**(32): p. 3838-3845.

28. Leemans, C.R., B.J. Braakhuis, and R.H. Brakenhoff, *The molecular biology of head and neck cancer.* Nat Rev Cancer, 2011. **11**(1): p. 9-22.

29. Ravindra Uppaluri, P.Z., Tianxiang Lin, Brian Nussenbaum, Ryan S Jackson, Jason Rich, Patrik Pipkorn, Randal Paniello, Wade Thorstad, Loren Michel, Tenny Mudianto, Peter John Oppelt, Tanya Marya Wildes, Gavin P Dunn, Jay F Piccirillo, Dorina Kallogjeri, Scott Rodig, Ian S. Hagemann, Rebecca Chernock, Douglas Adkins, *Neoadjuvant pembrolizumab in surgically resectable, locally advanced HPV negative head and neck squamous cell carcinoma (HNSCC)*, in *ASCO Abstract*. 2017.

30. Forde, P.M., et al., *Neoadjuvant PD-1 Blockade in Resectable Lung Cancer.* N Engl J Med, 2018. **378**(21): p. 1976-1986.

31. Ferris R.L., e.a., *An open-label, multicohort, phase 1/2 study in patients with virus-associated cancers (CheckMate 358): Safety and efficacy of neoadjuvant nivolumab in squamous cell carcinoma of the head and neck (SCCHN).* 2017. **28**(5).

32. *Nivolumab Investigator Brochure*.

33. Outh-Gauer, S., et al., *Immunotherapy in head and neck cancers: A new challenge for immunologists, pathologists and clinicians.* Cancer Treatment Reviews, 2018. **65**: p. 54-64.

34. Ferris, R.L., et al., *Nivolumab vs investigator's choice in recurrent or metastatic squamous cell carcinoma of the head and neck: 2-year long-term survival update of CheckMate 141 with analyses by tumor PD-L1 expression.* Oral Oncol, 2018. **81**: p. 45-51.

35. Hirsch, F.R., et al., *PD-L1 Immunohistochemistry Assays for Lung Cancer: Results from Phase 1 of the Blueprint PD-L1 IHC Assay Comparison Project.* Journal of Thoracic Oncology, 2017. **12**(2): p. 208-222.

36. Ock, C.Y., et al., *Changes in programmed death-ligand 1 expression during cisplatin treatment in patients with head and neck squamous cell carcinoma.* Oncotarget, 2017. **8**(58): p. 97920-97927.

37. Mammatas, L.H., et al., *Molecular imaging of targeted therapies with positron emission tomography: the visualization of personalized cancer care.* Cellular Oncology, 2015. **38**(1): p. 49-64.

38. Niemeijer, A.L., et al., *Whole body PD-1 and PD-L1 PET in pts with NSCLC.* Annals of Oncology, 2017. **28**.

39. Wong, K.H., et al., *The Predictive Value of Early Assessment After 1 Cycle of Induction Chemotherapy with F-18-FDG PET/CT and Diffusion-Weighted MRI for Response to Radical Chemoradiotherapy in Head and Neck Squamous Cell Carcinoma.* Journal of Nuclear Medicine, 2016. **57**(12): p. 1843-1850.

40. dos Anjos, R.F., et al., *Effectiveness of FDG-PET/CT for evaluating early response to induction chemotherapy in head and neck squamous cell carcinoma A systematic review.* Medicine, 2016. **95**(32).

41. Cho, S.Y., et al., *Prediction of Response to Immune Checkpoint Inhibitor Therapy Using Early-Time-Point F-18-FDG PET/CT Imaging in Patients with Advanced Melanoma.* Journal of Nuclear Medicine, 2017. **58**(9): p. 1421-1428.

42. Moy, J.D., J.M. Moskovitz, and R.L. Ferris, *Biological mechanisms of immune escape and implications for immunotherapy in head and neck squamous cell carcinoma.* Eur J Cancer, 2017. **76**: p. 152-166.

43. Freiser, M.E., P. Serafini, and D.T. Weed, *The immune system and head and neck squamous cell carcinoma: from carcinogenesis to new therapeutic opportunities.* Immunol Res, 2013. **57**(1-3): p. 52-69.

44. Solomon, B., R.J. Young, and D. Rischin, *Head and neck squamous cell carcinoma: Genomics and emerging biomarkers for immunomodulatory cancer treatments.* Semin Cancer Biol, 2018.

45. Teng, M.W., et al., *Classifying Cancers Based on T-cell Infiltration and PD-L1.* Cancer Res, 2015. **75**(11): p. 2139-45.

46. Balermpas, P., et al., *Tumour-infiltrating lymphocytes predict response to definitive chemoradiotherapy in head and neck cancer.* Br J Cancer, 2014. **110**(2): p. 501-9.

47. Fridman, W.H., et al., *The immune contexture in human tumours: impact on clinical outcome.* Nat Rev Cancer, 2012. **12**(4): p. 298-306.

48. Tsujikawa, T., et al., *Quantitative Multiplex Immunohistochemistry Reveals Myeloid-Inflamed Tumor-Immune Complexity Associated with Poor Prognosis.* Cell Rep, 2017. **19**(1): p. 203-217.

49. Schneiders, F.L., et al., *Circulating invariant natural killer T-cell numbers predict outcome in head and neck squamous cell carcinoma: updated analysis with 10-year follow-up.* J Clin Oncol, 2012. **30**(5): p. 567-70.

50. Braakhuis, B.J., et al., *Expression signature in peripheral blood cells for molecular diagnosis of head and neck squamous cell carcinoma.* Oral Dis, 2013. **19**(5): p. 452-5.

51. Lawrence, M.S., et al., *Discovery and saturation analysis of cancer genes across 21 tumour types.* Nature, 2014. **505**(7484): p. 495-501.

52. Leemans, C.R., P.J.F. Snijders, and R.H. Brakenhoff, *The molecular landscape of head and neck cancer.* Nat Rev Cancer, 2018. **18**(5): p. 269-282.

53. Karasaki, T., et al., *Prediction and prioritization of neoantigens: integration of RNA sequencing data with whole-exome sequencing.* Cancer Sci, 2017. **108**(2): p. 170-177.

54. Koshizuka, K., et al., *The microRNA signatures: aberrantly expressed microRNAs in head and neck squamous cell carcinoma.* J Hum Genet, 2017. **62**(1): p. 3-13.

55. Lindenbergh-van der Plas, M., et al., *Identification of lethal microRNAs specific for head and neck cancer.* Clin Cancer Res, 2013. **19**(20): p. 5647-57.

56. van Eijndhoven, M.A., et al., *Plasma vesicle miRNAs for therapy response monitoring in Hodgkin lymphoma patients.* JCI Insight, 2016. **1**(19): p. e89631.

57. Company, O.U.S.B.-M.S., *Opdivo Prescribing Information.* Product Information.

58. Niemeijer, A.L.N., et al., *Whole body PD-1 and PD-L1 PET with Zr-89-nivolumab and F-18-BMS-986192 in pts with NSCLC.* Journal of Clinical Oncology, 2017. **35**.

59. Boellaard, R., et al., *FDG PET/CT: EANM procedure guidelines for tumour imaging: version 2.0.* Eur J Nucl Med Mol Imaging, 2015. **42**(2): p. 328-54.

60. Santegoets, S.J., et al., *Myeloid derived suppressor and dendritic cell subsets are related to clinical outcome in prostate cancer patients treated with prostate GVAX and ipilimumab.* J Immunother Cancer, 2014. **2**: p. 31.

61. Tumeh, P.C., et al., *PD-1 blockade induces responses by inhibiting adaptive immune resistance.* Nature, 2014. **515**(7528): p. 568-71.

62. Scheinin, I., et al., *DNA copy number analysis of fresh and formalin-fixed specimens by shallow whole-genome sequencing with identification and exclusion of problematic regions in the genome assembly.* Genome Res, 2014. **24**(12): p. 2022-32.

63. Tang S., M.S. *neoantigenR: An annotation based pipeline for tumor neoantigen identification from sequencing data*. 2017 25-06-2018].

64. Snyder, A., et al., *Genetic basis for clinical response to CTLA-4 blockade in melanoma.* N Engl J Med, 2014. **371**(23): p. 2189-2199.

65. Boellaard, R., et al., *The Netherlands protocol for standardisation and quantification of FDG whole body PET studies in multi-centre trials.* Eur J Nucl Med Mol Imaging, 2008. **35**(12): p. 2320-33.

66. Boellaard, R., *Standards for PET image acquisition and quantitative data analysis.* J Nucl Med, 2009. **50 Suppl 1**: p. 11S-20S.

67. Wahl, R.L., et al., *From RECIST to PERCIST: Evolving Considerations for PET response criteria in solid tumors.* J Nucl Med, 2009. **50 Suppl 1**: p. 122S-50S.

68. Ferris, R.L., et al., *LBA46An open-label, multicohort, phase 1/2 study in patients with virus-associated cancers (CheckMate 358): Safety and efficacy of neoadjuvant nivolumab in squamous cell carcinoma of the head and neck (SCCHN).* Annals of Oncology, 2017. **28**(suppl_5): p. mdx440.041-mdx440.041.

69. Mandard, A.M., et al., *Pathologic assessment of tumor regression after preoperative chemoradiotherapy of esophageal carcinoma. Clinicopathologic correlations.* Cancer, 1994. **73**(11): p. 2680-6.

70. Langer, R. and K. Becker, *Tumor regression grading of gastrointestinal cancers after neoadjuvant therapy.* Virchows Arch, 2018. **472**(2): p. 175-186.

71. van den Eertwegh, A.J., et al., *Combined immunotherapy with granulocyte-macrophage colony-stimulating factor-transduced allogeneic prostate cancer cells and ipilimumab in patients with metastatic castration-resistant prostate cancer: a phase 1 dose-escalation trial.* Lancet Oncol, 2012. **13**(5): p. 509-17.

72. Santegoets, S.J., et al., *T cell profiling reveals high CD4+CTLA-4 + T cell frequency as dominant predictor for survival after prostate GVAX/ipilimumab treatment.* Cancer Immunol Immunother, 2013. **62**(2): p. 245-56.

**APPENDICES**

# APPENDIX 1: Response Evaluation Criteria in Solid Tumors (RECIST) 1.1 Criteria for evaluating Response in Solid Tumors

RECIST version 1.1* will be used in this study for assessment of tumor response with CT as the preferred imaging technique in this study.

* As published in the European Journal of Cancer:

E.A. Eisenhauer, P. Therasse, J. Bogaerts, L.H. Schwartz, D. Sargent, R. Ford, J. Dancey, S. Arbuck, S. Gwyther, M. Mooney, L. Rubinstein, L. Shankar, L. Dodd, R. Kaplan, D. Lacombe, J. Verweij. New response evaluation criteria in solid tumors: Revised RECIST guideline (version 1.1). Eur J Cancer. 2009 Jan;45(2):228-47.

# APPENDIX 2: Eastern Cooperative Oncology Group (ECOG) Performance Scale.

0 – Asymptomatic (Fully active, able to carry on all predisease activities without restriction)

1 – Symptomatic but completely ambulatory (Restricted in physically strenuous activity but ambulatory and able to carry out work of a light or sedentary nature. For example, light housework, office work)

2 – Symptomatic, <50% in bed during the day (Ambulatory and capable of all self care but unable to carry out any work activities. Up and about more than 50% of waking hours)

3 – Symptomatic, >50% in bed, but not bedbound (Capable of only limited self-care, confined to bed or chair 50% or more of waking hours)

4 – Bedbound (Completely disabled. Cannot carry on any self-care. Totally confined to bed or chair)

5 – Death

# APPENDIX 3: Management of toxicity due to nivolumab

Although toxicity due to a single administration of nivolumab is expected to be limited to acute toxicity, in exceptional cases immune related events may occur.

For subjects expected to require more than 4 weeks of corticosteroids or other immunosuppressants to manage an AE, consider recommendations provided in the algorithms. These algorithms can be found below.

*GI Adverse Event Management Algorithm*

Rule out non-inflammatory causes. If non-inflammatory cause is identified, treat accordingly and continue I-O therapy. Opiates/narcotics may mask symptoms of perforation. Infliximab should not be used in cases of perforation or sepsis.

Patients on IV steroids may be switched to an equivalent dose of oral corticosteroids (e.g. prednisone) at start of tapering or earlier, once sustained clinical improvement is observed. Lower bioavailability of oral corticosteroids should be taken into account when switching to the equivalent dose of oral corticosteroids.

*Renal Adverse Event Management Algorithm*

Rule out non-inflammatory causes. If non-inflammatory cause, treat accordingly and continue I-O therapy

Patients on IV steroids may be switched to an equivalent dose of oral corticosteroids (e.g. prednisone) at start of tapering or earlier, once sustained clinical improvement is observed. Lower bioavailability of oral corticosteroids should be taken into account when switching to the equivalent dose of oral corticosteroids.

*Pulmonary Adverse Event Management Algorithm*

Rule out non-inflammatory causes. If non-inflammatory cause, treat accordingly and continue I-O therapy. Evaluate with imaging and pulmonary consultation.

Patients on IV steroids may be switched to an equivalent dose of oral corticosteroids (e.g. prednisone) at start of tapering or earlier, once sustained clinical improvement is observed. Lower bioavailability of oral corticosteroids should be taken into account when switching to the equivalent dose of oral corticosteroids.

*Hepatic Adverse Event Management Algorithm*

Rule out non-inflammatory causes. If non-inflammatory cause, treat accordingly and continue I-O therapy. Consider imaging for obstruction.

Patients on IV steroids may be switched to an equivalent dose of oral corticosteroids (e.g. prednisone) at start of tapering or earlier, once sustained clinical improvement is observed. Lower bioavailability of oral corticosteroids should be taken into account when switching to the equivalent dose of oral corticosteroids.

*I-O therapy may be delayed rather than discontinued if AST/ALT ≤ 8 x ULN and T.bili ≤ 5 x ULN.

**The recommended starting dose for grade 4 hepatitis is 2 mg/kg/day methylprednisolone IV.

*Endocrinopathy Management Algorithm*

Rule out non-inflammatory causes. If non-inflammatory cause, treat accordingly and continue I-O therapy. Consider visual field testing, endocrinology consultation, and imaging.

Patients on IV steroids may be switched to an equivalent dose of oral corticosteroids (e.g. prednisone) at start of tapering or earlier, once sustained clinical improvement is observed. Lower bioavailability of oral corticosteroids should be taken into account when switching to the equivalent dose of oral corticosteroids.

*Skin Adverse Event Management Algorithm*

Rule out non-inflammatory causes. If non-inflammatory cause, treat accordingly and continue I-O therapy.

Patients on IV steroids may be switched to an equivalent dose of oral corticosteroids (e.g. prednisone) at start of tapering or earlier, once sustained clinical improvement is observed. Lower bioavailability of oral corticosteroids should be taken into account when switching to the equivalent dose of oral corticosteroids.

*Refer to NCI CTCAE v4 for term-specific grading criteria.

*Neurological Adverse Event Management Algorithm*

Rule out non-inflammatory causes. If non-inflammatory cause, treat accordingly and continue I-O therapy.

Patients on IV steroids may be switched to an equivalent dose of oral corticosteroids (e.g. prednisone) at start of tapering or earlier, once sustained clinical improvement is observed. Lower bioavailability of oral corticosteroids should be taken into account when switching to the equivalent dose of oral corticosteroids.

1. Women of childbearing potential

DEFINITIONS

**Woman of Childbearing Potential (WOCBP)**

A woman is considered fertile following menarche and until becoming post-menopausal unless permanently sterile. Permanent sterilization methods include hysterectomy, bilateral salpingectomy, and bilateral oophorectomy.

**Women in the following categories are not considered WOCBP**

- Premenarchal
- Premenopausal female with 1 of the following:
  - Documented hysterectomy
  - Documented bilateral salpingectomy
  - Documented bilateral oophorectomy

Note: Documentation can come from the site personnel’s review of the participant’s medical records, medical examination, or medical history interview.

- Postmenopausal female
  - A postmenopausal state is defined as 12 months of amenorrhea in a woman over age 45 years in the absence of other biological or physiological causes. In addition, females under the age of 55 years must have a serum follicle stimulating hormone, (FSH) level > 40 mIU/mL to confirm menopause.

CONTRACEPTION GUIDANCE FOR FEMALE PARTICIPANTS OF CHILD BEARING POTENTIAL

One of the highly effective methods of contraception listed below is required during study duration and until the end of relevant systemic exposure, defined as 5 months after the end of study treatment.*****

| **Highly Effective Contraceptive Methods That Are User Dependent**  *Failure rate of <1% per year when used consistently and correctly.*a |
| --- |
| - Combined (estrogen- and progestogen-containing) hormonal contraception associated with inhibition of ovulationb   - oral   - intravaginal   - transdermal |
| - Progestogen-only hormonal contraception associated with inhibition of ovulationb   - oral   - injectable |
| **Highly Effective Methods That Are User Independent** |
| - Implantable progestogen-only hormonal contraception associated with inhibition of ovulation b - Hormonal methods of contraception including oral contraceptive pills containing a combination of estrogen and progesterone, vaginal ring, injectables, implants and intrauterine hormone-releasing system (IUS)c - Intrauterine device (IUD)c - Bilateral tubal occlusion |
| - Vasectomized partner   *A vasectomized partner is a highly effective contraception method provided that the partner is the sole male sexual partner of the WOCBP and the absence of sperm has been confirmed. If not, an additional highly effective method of contraception should be used.* |
| - Sexual abstinence   *Sexual abstinence is considered a highly effective method only if defined as refraining from heterosexual intercourse during the entire period of risk associated with the study drug. The reliability of sexual abstinence needs to be evaluated in relation to the duration of the study and the preferred and usual lifestyle of the participant.*   - It is not necessary to use any other method of contraception when complete abstinence is elected. - WOCBP participants who choose complete abstinence must continue to have pregnancy tests, as specified in Section 2. - Acceptable alternate methods of highly effective contraception must be discussed in the event that the WOCBP participants chooses to forego complete abstinence |
| NOTES:  a Typical use failure rates may differ from those when used consistently and correctly. Use should be consistent with local regulations regarding the use of contraceptive methods for participants participating in clinical studies.  b Hormonal contraception may be susceptible to interaction with the study drug, which may reduce the efficacy of the contraceptive method. Hormonal contraception is permissible only when there is sufficient evidence that the IMP and other study medications will not alter hormonal exposures such that contraception would be ineffective or result in increased exposures that could be potentially hazardous. In this case, alternative methods of contraception should be utilized.  c Intrauterine devices and intrauterine hormone releasing systems are acceptable methods of contraception in the absence of definitive drug interaction studies when hormone exposures from intrauterine devices do not alter contraception effectiveness |

| **Unacceptable Methods of Contraception*** |
| --- |
| - Male or female condom with or without spermicide. Male and female condoms cannot be used simultaneously - Diaphragm with spermicide - Cervical cap with spermicide - Vaginal Sponge with spermicide - Progestogen-only oral hormonal contraception, where inhibition of ovulation is not the primary mechanism of action - Periodic abstinence (calendar, symptothermal, post-ovulation methods) - Withdrawal (coitus interruptus). - Spermicide only - Lactation amenorrhea method (LAM) |

*** Local laws and regulations may require use of alternative and/or additional contraception methods.**

CONTRACEPTION GUIDANCE FOR MALE PARTICIPANTS WITH PARTNER(S) OF CHILD BEARING POTENTIAL.

Male participants with female partners of childbearing potential are eligible to participate if they agree to the following during the treatment and until the end of relevant systemic exposure.

- Inform any and all partner(s) of their participation in a clinical drug study and the need to comply with contraception instructions as directed by the investigator.
- Male participants are required to use a condom for study duration and until end of relevant systemic exposure defined as 7 months after the end of study treatment.
- Female partners of males participating in the study to consider use of effective methods of contraception until the end of relevant systemic exposure, defined as 7 months after the end of treatment in the male participant.
- Male participants with a pregnant or breastfeeding partner must agree to remain abstinent from penile vaginal intercourse or use a male condom during each episode of penile penetration during the treatment and until 7 months after the end of study treatment.
- Refrain from donating sperm for the duration of the study treatment and until 7 months after the end of study treatment.
